# Dicoordinate Au(I)–Ethylene Complexes as Hydroamination Catalysts

**DOI:** 10.1021/acscatal.1c05823

**Published:** 2022-03-23

**Authors:** Miquel Navarro, Macarena G. Alférez, Morgane de Sousa, Juan Miranda-Pizarro, Jesús Campos

**Affiliations:** † Departamento de Química Inorgánica and Centro de Innovación en Química Avanzada (ORFEO-CINQA), Instituto de Investigaciones Químicas (IIQ), Consejo Superior de Investigaciones Científicas (CSIC) and University of Sevilla, Sevilla 41092, Spain

**Keywords:** gold catalysis, π-complexes, ethylene functionalization, hydroamination, bulky phosphines

## Abstract

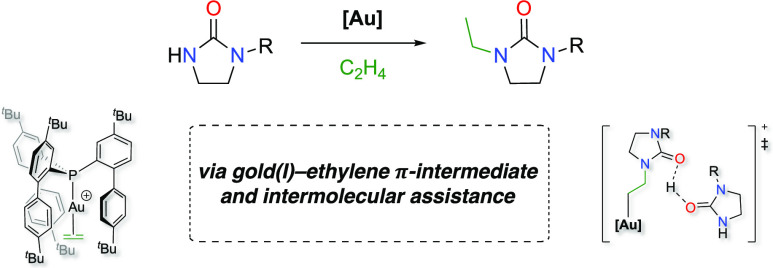

A series of gold(I)–ethylene π-complexes containing a family of bulky phosphine ligands has been prepared. The use of these sterically congested ligands is crucial to stabilize the gold(I)–ethylene bond and prevent decomposition, boosting up their catalytic performance in the highly underexplored hydroamination of ethylene. The precatalysts bearing the most sterically demanding phosphines showed the best results reaching full conversion to the hydroaminated products under notably mild conditions (1 bar of ethylene pressure at 60 °C). Kinetic analysis together with density functional theory calculations revealed that the assistance of a second molecule of the nucleophile as a proton shuttle is preferred even when using an extremely congested cavity-shaped Au(I) complex. In addition, we have measured a strong primary kinetic isotopic effect that is consistent with the involvement of X–H bond-breaking events in the protodeauration turnover-limiting step.

## Introduction

For decades, gold has been considered too chemically inert to be used in catalysis.^1^ However, since the discovery of its ability to activate π-bonds toward nucleophilic addition, molecular gold complexes have played a prominent role in the catalytic transformation of unsaturated hydrocarbons.^2^ The number of reactions mediated by π-acid gold catalysis is extensive and includes hydrogenation, oxidation, diarylation, heteroarylation, or cycloadditions, among many others.^3^ A type of transformation that has been extensively studied as a versatile route to prepare nitrogen-containing compounds with optimal atom economy is hydroamination, that is, the addition of an N–H unit of nucleophilic amines (or related substrates) across a carbon–carbon multiple bond.^4^ Although these processes can be catalyzed by other transition metals^5^ and even through metal-free protocols,^6^ gold(I) complexes remain one of the most powerful hydroamination catalysts.^3j,7,8^ In fact, they can accomplish the intermolecular hydroamination of C≡C triple bonds^9^ and even the more challenging C=C double bonds,^10,11^ in some cases even for inactivated alkenes. For the latter, the Au(I)-catalyzed hydroamination of ethylene, the simplest alkene, has only been reported once.^12^

Coordination of a C–C multiple bond to form a gold π-complex is usually proposed as the initial step during π-acid-catalyzed reactions, including hydroamination. Thus, the isolation of gold π-complexes has gathered considerable interest associated with their catalytic relevance, because they serve as models for the transient gold π-complexes.^13^ Among those, cationic dicoordinate gold(I) π-complexes of substituted alkenes and alkynes have been isolated and characterized over the last decade using phosphine or N-heterocyclic carbene ligands.^14^ Chelating N- and P-based ligands have also proved useful to form tricoordinate gold π-complexes.^15^ However, despite the interest in developing efficient methods for ethylene functionalization, gold(I)–ethylene complexes are quite rare; only 10 examples can be found in the literature and mainly using chelating ligands.^16,17^ In fact, we have recently authenticated the first dicoordinate gold(I)–ethylene adduct using the extremely bulky tris-2-(4,4′-di-*tert*-butylbiphenylyl)phosphine (**L1**), previously reported by Straub,^18^ that kinetically stabilizes the coordination of ethylene.^19^ In contrast to related tricoordinate complexes, the bonding interactions are mainly electrostatic (i.e., ionic) with minimal Au → ethylene π-backdonation.

**Figure 1:**
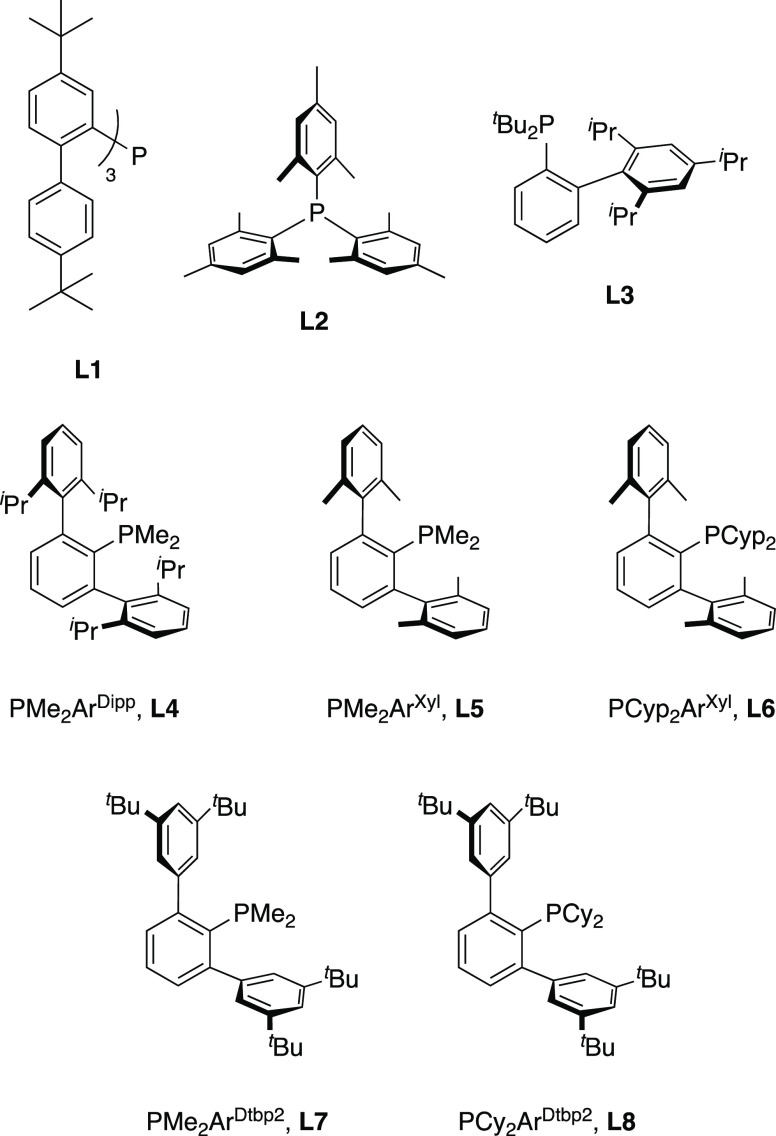
Selected bulky phosphine ligands used in this study.

This strategy of kinetic stabilization using sterically demanding ligands to detect and isolate transient intermediates of relevance to catalytic processes has proved successful in the past. Our group has also committed to the task, capitalizing on the steric shrouding provided by terphenyl (C_6_H_3_-2,6-Ar_2_) phosphine ligands.^20^ For instance, these have been used to access unusual gold compounds, such as the first methyl-bridged cationic digold complexes^21^ and to study their relevance in C–C coupling processes,^22^ as well as to exploit gold species as frustrated Lewis pair constituents.^23^ In this study, we have selected a family of bulky phosphine ligands in an attempt to access rare Au(I)–ethylene adducts. More precisely, we have used both the commercial ligands trimesityl phosphine (**L2**) and ^*t*^BuXPhos (**L3**), as well as a series of terphenyl phosphines (**L4**–**L8**) prepared by our group (Figure 1).^24^ We compare herein the stability of the resulting ethylene adducts with respect to the first of its class constructed around **L1**(19) and examine their catalytic competence for the underdeveloped Au(I)-catalyzed functionalization of ethylene through a model hydroamination reaction.

## Results and Discussion

### Synthesis of Gold(I)–Ethylene Complexes

The reaction of [AuCl(THT)] (THT = tetrahydrothiophene) with phosphine ligands **L1**–**L8** in dichloromethane forms the air-stable, neutral phosphine chloride complexes **1**–**8**. The steric bulkiness of the phosphine ligands was evaluated calculating the percent buried volume (%*V*_bur_),^25^ which yielded notably large parameters (Table 1 and Figure S86). Nonetheless, there are clear differences in the steric shrouding imparted by the employed phosphines. Terphenyl phosphine ligands containing two small methyl groups bound to the phosphorus atom present lower %*V*_bur_ values, ranging from 38.2 in **L5** to 46.2 in **L4** after substituting the methyl groups on the flanking aryl rings of the terphenyl substituent by isopropyl termini. Similar %*V*_bur_ parameters were measured for **L7** and the widely used trimesityl phosphine (**L2**). Introducing bulkier substituents bound to the phosphorus atom in **L6** and **L8** increased the %*V*_bur_ to around 53, comparable to that of the Buchwald-type phosphine **L3**. Albeit the former are considerably bulky, the tris biaryl tris-2-(4,4′-di-*tert*-butylbiphenylyl)phosphine phosphine (**L1**) clearly presents the highest %*V*_bur_ value of 67.0.^19^ As discussed in the following sections, the steric profile of the ligand seems to be crucial to impart stability to the aimed Au(I)–ethylene compounds, having a direct effect on catalytic performance.

**Table 1:** Selected Spectroscopic and Structural Data of Complexes **1**–**8**·C_2_H_4_

compound	δ^1^H (ppm)	δ^13^C (ppm)	δ^31^P (ppm)^a^	%*V*_bur_^b^	*d*(C=C) (Å)
C_2_H_4_	5.43	116.8			1.313^c^
**1·C_2_H_4_**	3.66; 3.79	110.2	13.1 (9.5)	67.0	1.236(10)^d^
**2·C_2_H_4_**	5.46	111.2	1.5 (−5.4)	45.3	
**3·C_2_H_4_**	4.95	110.9	65.6 (58.6)	55.5	1.353(15)
**4·C_2_H_4_**	4.85	110.3	4,3 (−5.7)	46.2	
**5·C_2_H_4_**	5.00	111.5	4.1 (−3.2)	38.2	
**6·C_2_H_4_**	4.86	111.0	57.6 (53.3)	53.5	
**7·C_2_H_4_**	5.16	111.8	9.4 (0.4)	45.0	
**8·C_2_H_4_**	4.77	109.0	55.4 (48.8)	53.7	1.384(10)

a The corresponding δ^31^P NMR of the gold chloride complexes **1**–**8** is indicated in parentheses.

b %*V*_bur_ is calculated from the corresponding gold(I) chloride complexes **1**–**8** (see the SI for more details).

c Data from ref (26).

d Data from ref (19).

**Scheme 1:**
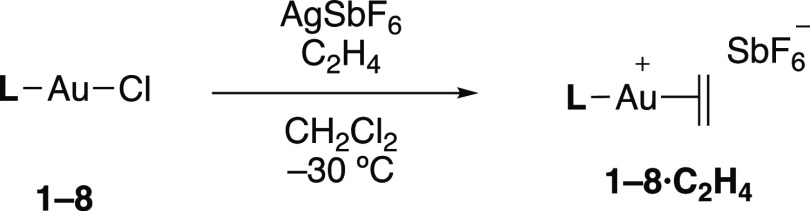
Synthesis of Gold(I)–Ethylene Complexes **1**–**8**·C_2_H_4_ (L = **L1**–**L8** from Figure 1)

Treatment of gold(I) chloride complexes **1**–**8** with AgSbF_6_ under an ethylene atmosphere at −30 °C caused instantaneous precipitation of AgCl and formation of the gold(I)–ethylene complexes **1**–**8**·**C_2_H_4_** (Scheme 1). Filtration of the aforementioned reaction mixtures through short pads of Celite followed by washing with pentane afforded the pure gold(I) π-complexes **1**–**8**·**C_2_H_4_** in good to excellent yields (53–93%). The reactions were conveniently monitored by ^31^P{^1^H} NMR spectroscopy, which revealed a systematic downfield shift in the range from 4.3 to 10.0 ppm compared to the corresponding gold(I) chloride complexes (Table 1). It is worth noting that attempts to prepare the related [(Ph_3_P)Au(C_2_H_4_)]^+^ complex led to immediate decomposition and formation of [(PPh_3_)_2_Au]^+^ and Au(0), likely because of the inability of the relatively small PPh_3_ ligand to kinetically stabilize the corresponding Au(I)–ethylene adduct.

Complexes **2**–**8**·**C_2_H_4_** were spectroscopically characterized in dichloromethane solution under an ethylene atmosphere to prevent decomposition, which accelerates upon removal of the gaseous substrate. In some cases and because of the chemical exchange between coordinated and free ethylene (*vide infra*), the two signals were undistinguishable. To unambiguously identify the resonances belonging to coordinated ethylene, ^1^H NMR spectroscopy and ^13^C{^1^H} NMR spectroscopy were also performed in the absence of ethylene, though in those cases signs of decomposition were evident from NMR spectroscopy results (see the SI for more details). Nonetheless, these studies permitted the unambiguous assignment of the targeted ethylene adducts; resonances associated with the coordinated olefin were found to differ from those of the free molecule (Table 1). Thus, coordination to gold(I) induces a noticeable upfield shift of the ^1^H NMR signals (∼0.5 ppm) with the exception of complex **2**·**C_2_H_4_**, which is only slightly downfield shifted by 0.06 ppm. In turn, ^13^C{^1^H} NMR resonances are shifted in the same direction with upfield shifts about 7 ppm with respect to free ethylene (Table 1). These relatively small changes suggest little backdonation from Au to the ethylene π*(C=C) orbital, as noted earlier for **1**·**C_2_H_4_**,^19^ and in contrast with the related tricoordinate gold(I)–ethylene complexes,^13f^ in which the chemical shift differences can reach up to 3 and 55 ppm in ^1^H and ^13^C NMR spectra, respectively. As for the more sterically hindered complex **1**·**C_2_H_4_**,^19^ the coordinated ethylene presented the largest shift in ^1^H NMR resonances, which appear as an AA′BB′ system at 3.79 and 3.66 ppm, contrasting with the rest of the compounds that led to a single broad peak due to four equivalent protons. We ascribed the shift in **1**·**C_2_H_4_** to ring-current effects due to the surrounding aryl rings, which could also hinder the rotation of bound ethylene giving rise to the observed AA′BB′ system. Chemical exchange between coordinated and free ethylene was observed in CD_2_Cl_2_ within the NMR timescale for all ethylene adducts; however, its rate could not be reliably quantified because of the rapid exchange even at low temperature and the close proximity of the respective NMR signals, which prevented accurate data analysis (see Figures S57–S61 in the Supporting Information).

**Figure 2:**
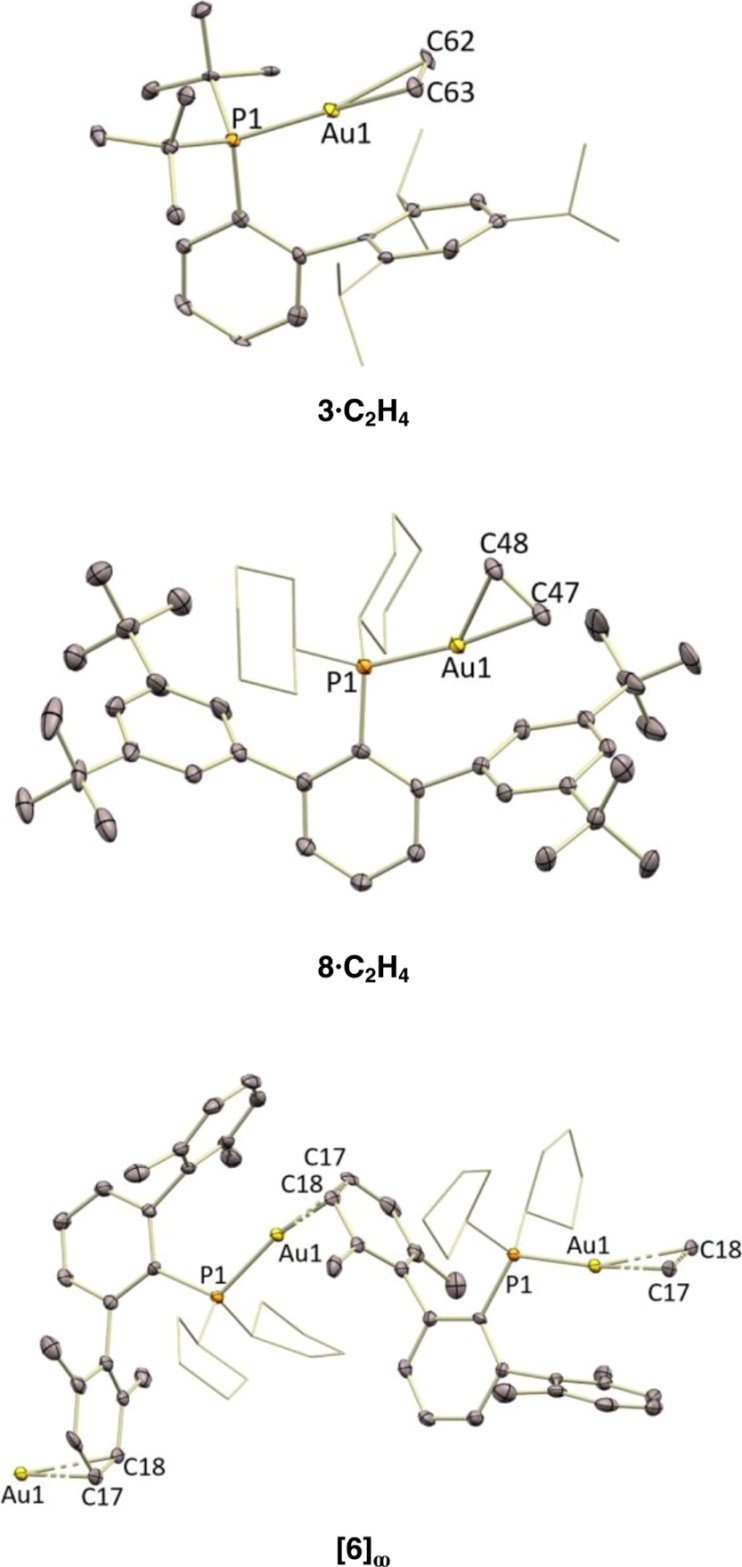
ORTEP representation of complexes **3**·**C_2_H_4_**, **8**·**C_2_H_4_**, and **[6]_∞_**. Thermal ellipsoids are set at 50% probability. Counteranions, solvent molecules, and hydrogen atoms are excluded for clarity, while iso-propyl and cyclohexyl groups are represented in wireframe format. Selected bond length (Å) and angles (°): compound **3**·**C_2_H_4_**, (one of two independent molecules per asymmetric unit; selected parameters from the one not showing disorder in the ethylene ligand), P1–Au1, 2.289(2); Au1–C62, 2.237(9); Au1–C63, 2.261(9); C62–C63, 1.353(15); P1–Au1–C62, 149.2(3); P1–Au1–C63, 164.8(3); compound **8**·**C_2_H_4_**, P1–Au1, 2.2977(16); Au1–C47, 2.210(7); Au1–C48, 2.227(7); C47–C48, 1.384(10); P1–Au1–C47, 173.54(19); P1–Au1–C48, 137.2(2); compound **[6]_∞_**, P1–Au1, 2.2633(15); Au1–C17, 2.301(6); Au1–C18, 2.403(6); C17–C18, 1.385(11); P1–Au1–C17, 169.2(2); P1–Au1–C18, 151.5(2).

Single crystals of complexes **3**·**C_2_H_4_** and **8**·**C_2_H_4_** suitable for X-ray diffraction analysis were obtained by slow diffusion of pentane into saturated dichloromethane solutions of the gold(I)–ethylene complexes at −30 °C. Both species adopt similar structures in the solid state, with the gold center in a linear environment and the ethylene molecule coordinated in an η^2^-fashion (Figure 2). It is worth noting that in contrast to complexes **1**·**C_2_H_4_** and **3**·**C_2_H_4_**, the coordination of ethylene to gold in **8**·**C_2_H_4_** is highly nonsymmetric: the ethylene molecule is notably slipped, that is, whereas it presents similar Au–C bond distances, the P–Au–C angles of 173.55(19)° and 137.2(2)° are remarkably different. In complexes **3**·**C_2_H_4_** and **8**·**C_2_H_4_**, the Au–C bond lengths (2.21–2.26 Å) are noticeably longer than those described for gold(I)–ethylene adducts bearing bidentate ligands (ca. 2.14–2.17 Å),^15,16^ but similar to **1**·**C_2_H_4_** (2.216(6) and 2.235(6) Å) and related cationic dicoordinate gold(I) π-complexes of other alkenes.^14^ The C=C double bond (**3**·**C_2_H_4_**, 1.353(15) Å; **8**·**C_2_H_4_**, 1.384(10) Å) is slightly longer than that of free ethylene (1.313 Å)^26^ and complex **1**·**C_2_H_4_** (1.263(10) Å) and similar to those described for tricoordinate gold(I) ethylene compounds,^16^ despite the expected poor Au → ethylene π-backdonation.

It was mentioned above that gold(I)–ethylene complexes **2**–**8**·**C_2_H_4_** exhibit slow decomposition both in the solid state and in dichloromethane solution upon removal of the ethylene atmosphere, which contrasts with the remarkable stability of **1**·**C_2_H_4_** that we have attributed to the kinetic stabilization imparted by the cavity-shaped phosphine. For all other cases, monitoring the evolution of dichloromethane solutions of the ethylene adducts by ^31^P{^1^H} NMR spectroscopy revealed the presence of the corresponding [P–Au–P]^+^ decomposition products along with Au(0) nanoparticles as the major products.^22,27,28^ Nonetheless, the appearance of other broad ^31^P{^1^H} signals evinces the formation of additional species. For instance, after a few days in solution the decomposition spectrum of complex **6**·**C_2_H_4_** revealed the formation of a relatively broad ^31^P{^1^H} NMR signal at 50.3 ppm distinct to the one corresponding to [(PCyp_2_ArXyl_2_)_2_Au]^+^ (53.4 ppm). X-ray diffraction studies allowed us to ascertain the formation of a new gold(I) cationic species (**[6]_∞_**) with a highly unusual polymeric structure derived from ethylene release and subsequent η^2^-coordination of a side aryl ring of the terphenyl substituent of an adjacent cationic gold fragment (Figure 2). The η^2^-coordination of the xylyl ring is slightly slipped with different Au–C distances of 2.301(6) and 2.401(7) Å and notably different P–Au–C angles of 169.2(2)° and 151.5(2)°, respectively. This structure is reminiscent of π-arene complexes of gold formed in aromatic solvents, which have been reported in several occasions and whose geometric parameters are comparable to **[6]_∞_**.^29^ However, this seems to be the first polymeric structure of this kind in which the building blocks are solely units of [LAu]^+^ connected by π-coordination.

Attempts to prepare other polymeric structures of this type by direct treatment of compounds **1**–**8** with equimolar amounts of AgSbF_6_ in dichloromethane were unsuccessful. In fact, while under an ethylene atmosphere instant precipitation of AgCl upon addition of the silver reagent was visually identified, this did not occur in the absence of the olefin, arguing in favor of the presence of silver within the resulting structure. This was not surprising considering our previous report on the reaction of complex **1** and AgSbF_6_, which resulted in the formation of a gold–silver trimetallic species without chloride abstraction. In the case of compounds **2** and [(Ph_3_P)AuCl], generation of the corresponding homoleptic [P–Au–P]^+^ complexes and Au(0) nanoparticles was exclusively observed. In contrast, complexes **3**–**8** bearing bulky biphenyl and terphenyl phosphine ligands do not lead to their corresponding [P–Au–P]^+^ complexes but form instead other species characterized by broad NMR resonances that we tentatively attribute to gold(I)–silver(I) multimetallic complexes by analogy with our prior studies on compound **1** (see Figures S64 and S65 in the Supporting Information).^30^ This notion is further supported by diffusion-ordered NMR experiments. For instance, ^1^H DOSY experimental data revealed a diffusion coefficient for the in situ equimolar reaction between complex **6** and AgSbF_6_, D equal to 9.13 × 10^–10^ m/s^2^ that accounts for only half of that for pure **6**·**C_2_H_4_** (*D* = 1.75 × 10^–9^ m/s^2^), (see Figures S63 and S64 for more details), indicating a larger structure attributable to a multimetallic species in the former case.

### Catalytic Hydroamination of Ethylene

Having on hand the first examples of stable dicoordinate Au(I)–ethylene compounds, we next examined their catalytic potential in the hydroamination of ethylene. Initially, imidazolidine-2-one (**9**)^12^ was used as a model substrate to gauge the activity of all cationic gold(I)-ethylene species, obtained in situ from its corresponding neutral chloride precursors. Analogous to the conditions reported in Widenhoefer’s seminal investigations on intermolecular olefin hydroamination,^12^ solutions of compound **9** were pressurized with ethylene (4 bar) in the presence of 5 mol % of the gold(I) chloride complex **1**–**8** and 5 mol % of AgSbF_6_ as a halide scavenger in dioxane at 100 °C. Complexes **1**, **3**, **6**, and **8** displayed great catalytic activity, reaching full conversion to the double hydroamination product 1,3-ethylimidazolidin-2-one (**10**) after 18 h (Table 2, entries 1, 4, 7, and 9), while formation of the monohydroaminated species was not detected. Interestingly, these complexes bear the bulkier phosphine ligands, with %*V*_bur_ values between 53.5 and 67.0. On the contrary, low or no conversion was obtained when employing complexes **2**, **4**, **5**, **7**, and [(Ph_3_P)AuCl] (Table 2, entries 3, 5, 6, 8, and 10), which present smaller phosphine ligands with %*V*_bur_ below 46.2. In addition, the previously isolated gold–ethylene complex **1**·**C_2_H_4_** was used as a catalyst reaching full conversion under our optimized conditions (Table 2, entry 26).

**Table 2:** Gold(I)-Catalyzed Hydroamination of Ethylene by Imidazolidine-2-one^a^

entry	catalyst	*P*_C2H4_ (bar)	conversion (%)^b^	**10**:**11**
1	**1**	4	>99	100:0
2	**1·MeCN**^c^	4	>99	100:0
3	**2**	4	0	-
4	**3**	4	>99	100:0
5	**4**	4	<5	n.d.^d^
6	**5**	4	<5	n.d.^d^
7	**6**	4	95	100:0
8	**7**	4	20	15:85
9	**8**	4	>99	100:0
10	[(Ph_3_P)AuCl]	4	0	
11		4	0	
12	**L1**	4	0	
13	**L3**	4	0	
14	**1**	2	>99	100:0
15	**1·MeCN**^c^	2	98	100:0
16	**3**	2	>99	100:0
17	**6**	2	50	35:65
18	**8**	2	56	n.d.^d^
19	**1**	1	98	100:0
20	**1·MeCN**^c^	1	50	35:65
21	**3**	1	95	100:0
22	**6**	1	11	10:90
23	**8**	1	30	35:65
24	**1**^e^	1	64	30:70
25	**3**^e^	1	50	20:80
26	**1·C_2_H_4_**	1	>99	100:0
27	**1**^f^	1	>99	100:0
28	**1**^g^	1	96	66:33
29	**3**^f^	1	70	31:69
30	**3**^g^	1	64	37:63
29	**13**	1	>99	100:0

a Reaction was performed with imidazolidine-2-one (0.20 mmol) under the indicated ethylene pressure, gold catalyst (0.01 mmol), and AgSbF_6_ (0.01 mmol) as a chloride abstractor in 1,4-dioxane (1 mL) at 100 °C for 18 h.

b Conversion was determined by ^1^H NMR spectroscopy with anisole as the internal standard.

c In the absence of AgSbF_6_.

d Not determined (n.d).

e Catalyst loading at 2 mol %.

f Reaction at 80 °C.

g Reaction at 60 °C.

**Scheme 2:**
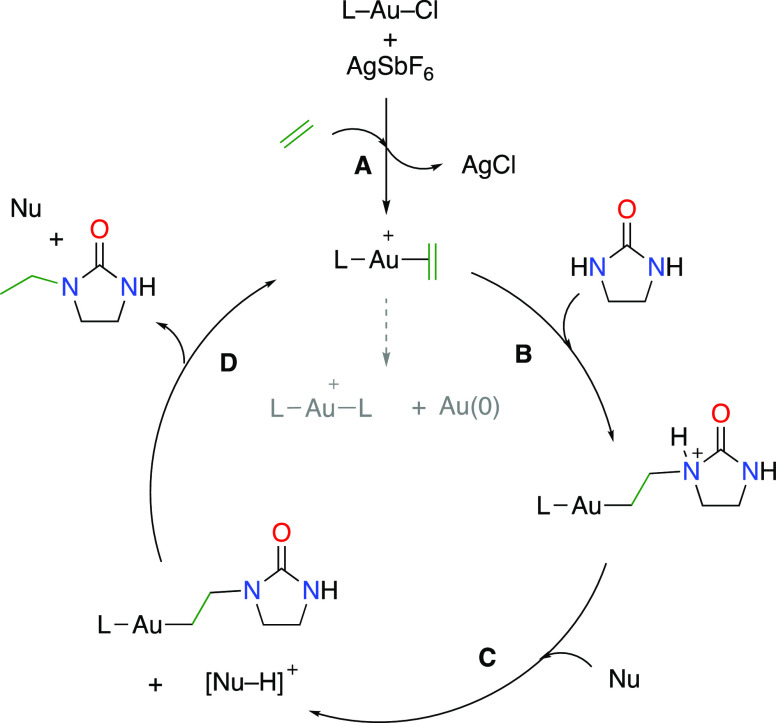
Proposed Mechanism for the Assisted Hydroamination of Ethylene Deactivation of the gold(I) precatalyst by the formation of [P–Au–P]^+^ and Au(0) nanoparticles is indicated with a dashed gray arrow.

^31^P{^1^H} NMR spectroscopy analysis of the final catalytic mixtures after 18 h revealed the presence of the independently authenticated gold(I)–ethylene complexes in most cases, together with variable amounts of the corresponding free phosphine ligands. However, in the case of **2**, **4**, **5**, and [(Ph_3_P)AuCl], the corresponding [P–Au–P]^+^ complexes were clearly observed as the major or sole gold-containing species. Formation of the latter under catalytic conditions is in agreement with our prior stability studies and can be understood as a deactivation pathway for the gold(I) complexes bearing the smaller phosphine ligands (Scheme 2), while more hindered phosphines prevent or slow down this unproductive route. Control experiments were also performed to investigate whether the presence of silver ions could have a direct influence on the catalytic outcome, as previously reported in other gold-catalyzed processes.^31^ No conversion was observed in the absence of the gold(I) complex (Table 2, entry 11) or in the presence of a combination of 5 mol % of **L1** or **L2** and AgSbF_6_ (Table 2, entries 12 and 13), indicating that under these conditions silver(I) is not capable of catalyzing the hydroamination of ethylene. In addition, the solvento complex **1·MeCN**(19) was used in the absence of AgSbF_6_ achieving full conversion after 18 h, ruling out a direct silver-effect during gold catalysis (Table 2, entry 2).^31^

Complex **1**·**MeCN** reaches full conversion with 2 bar of ethylene (Table 2, entry 15) but only 50% conversion under 1 bar of ethylene pressure (Table 2, entry 20). In this case, the presence of acetonitrile may compete with ethylene coordination at low pressure, as we have proved before,^19^ decreasing its catalytic activity. Complexes **6** and **8** only reach moderate conversions of ∼50% at 2 bar of ethylene pressure (Table 2, entries 17 and 18) and 30% and 11% (Table 2, entries 22 and 23) at 1 bar of ethylene pressure, respectively. For the latter two complexes, a mixture of the mono- (**11**) and dihydroaminated (**10**) products was detected by ^1^H NMR spectroscopy, suggesting that the double hydroamination process proceeds in a stepwise manner.

Considering the potential role of water as a proton shuttle in gold catalysis,^32^ and having in mind the existence of such proton rearrangements in this transformation (*vide infra*), we decided to investigate the addition of water and other additives. The results of these studies are collected in Table S1 in the Supporting Information. The presence of 10 mol % or 10 equiv of H_2_O or HFIP did not show any significant effect on the conversion. On the other hand, the presence of small amounts (or excess) of acids such as HOTf or CH_3_COOH has a detrimental effect on the catalytic transformation. Different bases such as ^*t*^BuOK, Et_3_N, and DBU were also employed, which caused a complete shutdown of the catalytic activity.

Next, to compare better the reactivity of the most active catalysts, complexes **1** and **3**, we investigated the hydroamination of ethylene under milder conditions. These two catalysts reach full conversion after 18 h when 2 and 1 bar of ethylene pressure was used (Table 2, entries 14, 16, 19, and 21). However, conversion drops to 64 and 50%, respectively, when the catalyst loading is lowered to 2 mol % at 1 bar of ethylene pressure at 100 °C (Table 2, entries 24 and 25). Remarkably, complex **1** also reaches full conversion even at 1 bar of ethylene pressure when the temperature is lowered to 80 °C and 96% conversion at only 60 °C (Table 1 entries 27 and 28). In contrast, conversion is reduced to 70 and 64% using complex **3** at 80 and 60 °C, respectively (entries 29 and 30). These results confirm the benefits of using the extremely bulky ligand **L1**, which slightly outperforms even the highly active catalyst based on **L3** under particularly mild conditions.

Complex **1** is also able to successfully convert 1-methyl-imdazolidine-2-one (**9′**) into 1-methyl-2-ethylimidazolidin-2-one (**10′**) at 1 bar of ethylene pressure after 18 h at 60 °C, while only traces (<10%) of the hydroaminated product were observed when 2-oxazolidinone was used as a substrate even at 4 bar of ethylene pressure at 100 °C (see Supporting Information, Table S2, entries 1–4). In contrast, acyclic amide substrates could not be converted. Bulky amines, such as diisopropylamine or *tert*-butylamine, were also tested as substrates using gold(I) complexes **1** and **3**, but no conversion was observed. In these cases, new signals were detected in the ^31^P{^1^H} NMR spectra of the final mixtures that differ from the corresponding gold(I) chloride and gold(I) π-ethylene complexes. For instance, from the reaction of complex **1** with diisopropylamine under catalytic conditions, a single crystal suitable for X-ray diffraction analysis was isolated and analyzed, confirming the coordination of the amine to the electrophilic Au(I) center (Figure S84) to form the corresponding [P–Au–NH^*i*^Pr_2_]^+^ complex **12**. Surprisingly, even more hindered amines like *N*-benzhydrylpropan-2-amine and tetramethylpiperidine are capable of displacing the ethylene molecule in gold(I) complex **1**·**C_2_H_4_** to yield gold adducts analogous to **12**, as inferred from their corresponding ^31^P{^1^H} and ^1^H NMR spectra (Figure S66, see the SI for more details). Thus, the extreme steric profile of **L1** does not seem to prevent amine coordination and as such the targeted nucleophilic attack of the amine toward the electrophilic carbon of the coordinated ethylene does not occur, preventing the initiation of the catalytic hydroamination process.

**Figure 3:**
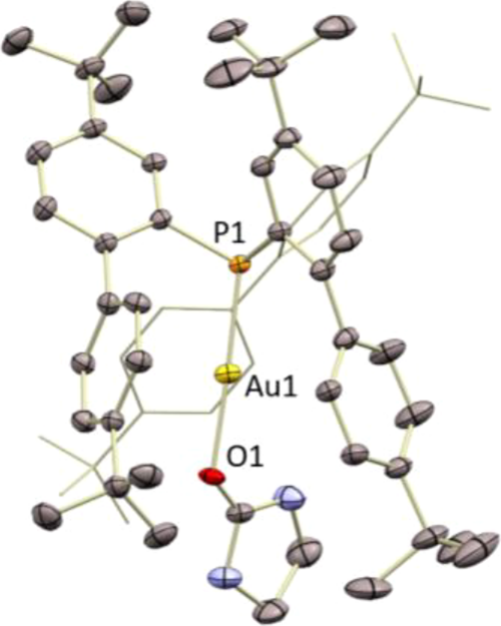
ORTEP representation of complex **13**. Thermal ellipsoids are set at 50% probability. Counteranions and hydrogen atoms are excluded for clarity, while tert-butyl groups and one biaryl fragment are represented in wireframe format. Selected bond length (Å) and angles (°): P1–Au1, 2.2084(12); Au1–O1, 2.083(3); P1–Au1–O1, 177.54(12).

In view of these results, we decided to attempt the isolation of the Au(I)–imidazolidinone adduct that could act as an intermediate in the catalytic cycle. Complex **1** was reacted with AgSbF_6_ and imidazolidine-2-one **9** to form the corresponding complex **13**, which was isolated as a stable solid under an inert atmosphere showing no decomposition at room temperature. Single crystals of complex **13** suitable for X-ray diffraction analysis were obtained by slow diffusion of pentane into a saturated dichloromethane solution of the gold(I) complex **13** at −30 °C. Complex **13** presents a solid-state structure with the gold center in a linear environment. In contrast to the isopropylamine ligand in complex **12**, and despite the low oxophilicity of gold, imidazolidine-2-one coordinates at the metal through the oxygen atom (Figure 3). Complex **13** was utilized as a precatalyst affording full conversion toward hydroaminated ethylene within 18 h under the optimized conditions (Table 2, entry 31). To investigate the role of complex **13** in the mechanism, a solution of complex **13** in CD_2_Cl_2_ was charged with 1 bar of ethylene pressure showing full imidazolidine-2-one replacement by ethylene to exclusively form the gold–ethylene complex **1**·**C_2_H_4_**, suggesting that under the catalytic conditions the presence of complex **13** is unlikely.

The catalytic activity of complex **1** toward the hydroamination of different 1-alkenes was also investigated (Table S2, see the Supporting Information for more details). Complex **1** converts imidazolidine-2-one to 1,3-diisopropylimidazolidin-2-one with complete Markovnikov regioselectivity at 6 bar of propene pressure after 18 h at 100 °C, while only 76% conversion is achieved when lowering the propene pressure to 4 bar. Complex **1** also fully converts 1-methyl-imdazolidine-2-one (**9′**) to 1-isopropyl-3-methylimidazolidin-2-one at 3 bar propene pressure after 18 h at 100 °C (87% conversion at 2 bar of propene pressure). The corresponding gold(I) π-propene complex **14** was also isolated and fully characterized, and X-ray diffraction analysis revealed a similar structure compared to the gold(I)–ethylene adduct **1**·**C_2_H_4_**. The gold center is in a linear environment with the propene molecule coordinating gold in an η^2^ fashion (Figure S85). The Au–C bond lengths in the two independent molecules present in the asymmetric unit (2.22–2.27 Å) are similar to those in complex **1**·**C_2_H_4_** and the aforementioned ethylene adducts **3**·**C_2_H_4_** and **8**·**C_2_H_4_**. The C=C double bond appears artificially shortened because of some degree of disorder on the olefin fragment and cannot be reliably determined. In addition, 1-alkenes with longer chains like 1-octene and cyclic alkenes such as cyclopentene and cyclohexene (Table S2, entries 12–17) are only moderately converted when using 1-methyl-imdazolidine-2-one (19–66%) at 100 °C even after longer reaction times (66 h). In contrast, 1-alkenes with bulkier substituents like 3,3-dimethyl-1-butene and styrene were not converted at all (<5% conversion, Table S2, entries 18 and 19), most likely because of the high steric profile of **L1**.

### Mechanistic Considerations of the Gold(I)-Catalyzed Hydroamination of Ethylene

The gold(I)-catalyzed hydroamination of alkenes and related substrates has been recently studied computationally by Lledós and co-workers.^10b,11c^ The proposed mechanism (Scheme 2) was described as a typical π-catalysis activation pathway, involving the coordination of the alkene to the gold(I) center (A) followed by the nucleophilic addition of the amide to the activated olefin (B). The next step involves the protodeauration process assisted by a proton shuttle (second amide molecule, C and D) to generate the hydroaminated product.

In this report, we have demonstrated that the use of sterically hindered phosphines is crucial to achieve good activities, which we attribute to the higher stability that they impart to the key π-ethylene intermediates, preventing (or slowing down) the formation of the corresponding [P–Au–P]^+^ complexes as the main deactivation route. In particular, the use of complex **1**·**C_2_H_4_**, bearing an extremely bulky phosphine ligand, has given (along with **3**·**C_2_H_4_** to a slightly lesser extent) the best catalytic activities in this transformation. Therefore, we sought to gain mechanistic insight into this system, by means of kinetic experiments and density functional theory (DFT) calculations,^33^ to assess whether the previously proposed reaction mechanism^11c^ was affected by the steric hindrance of phosphine **L1** in the catalytic process.

**Figure 4:**
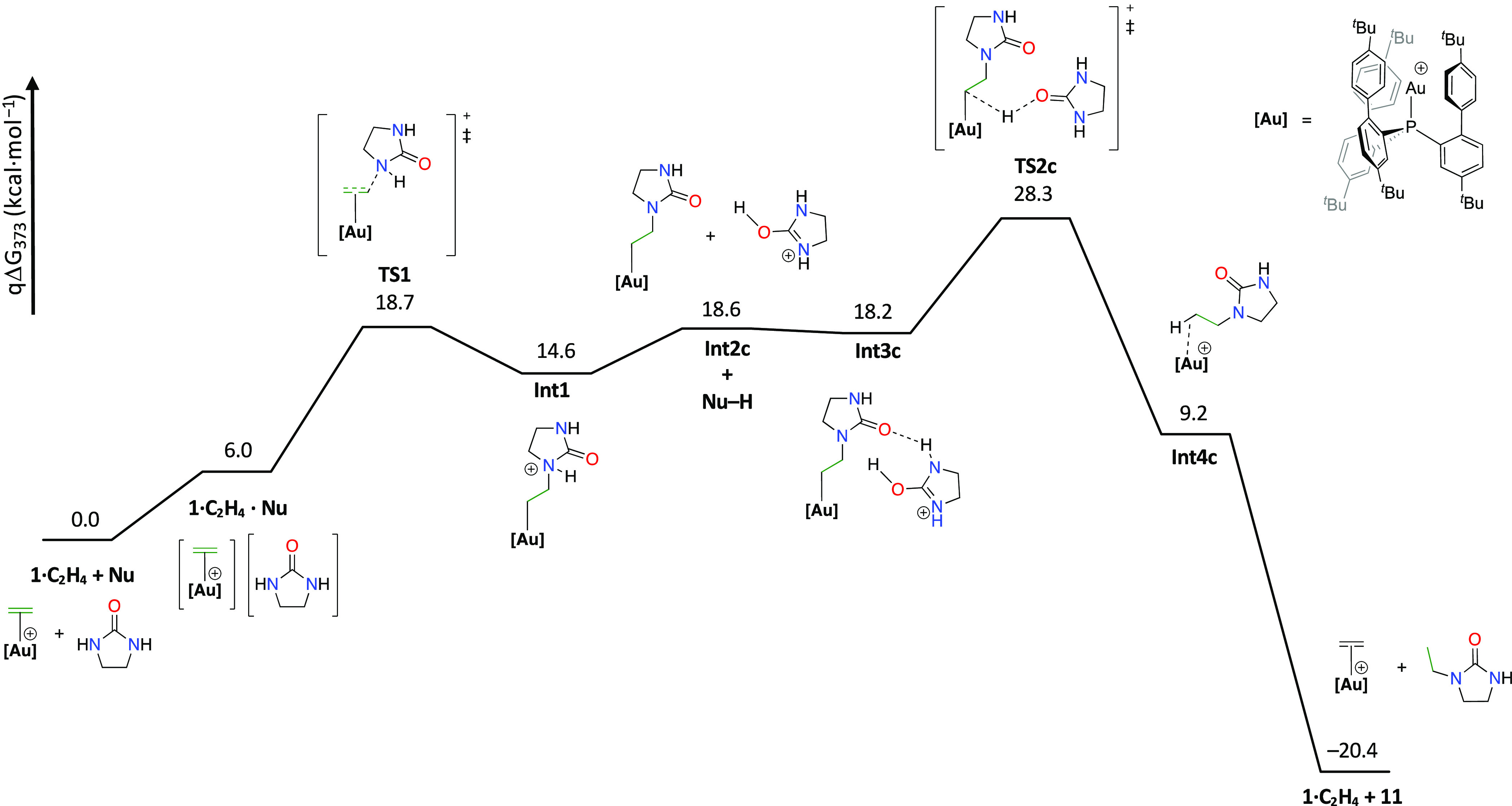
Free energy profile for the Au(I)-catalyzed hydroamination of ethylene with imidazolidine-2-one (**9** = Nu) assisted by a second molecule of imidazolidine-2-one acting as a proton shuttle (path C).

**Scheme 3:**
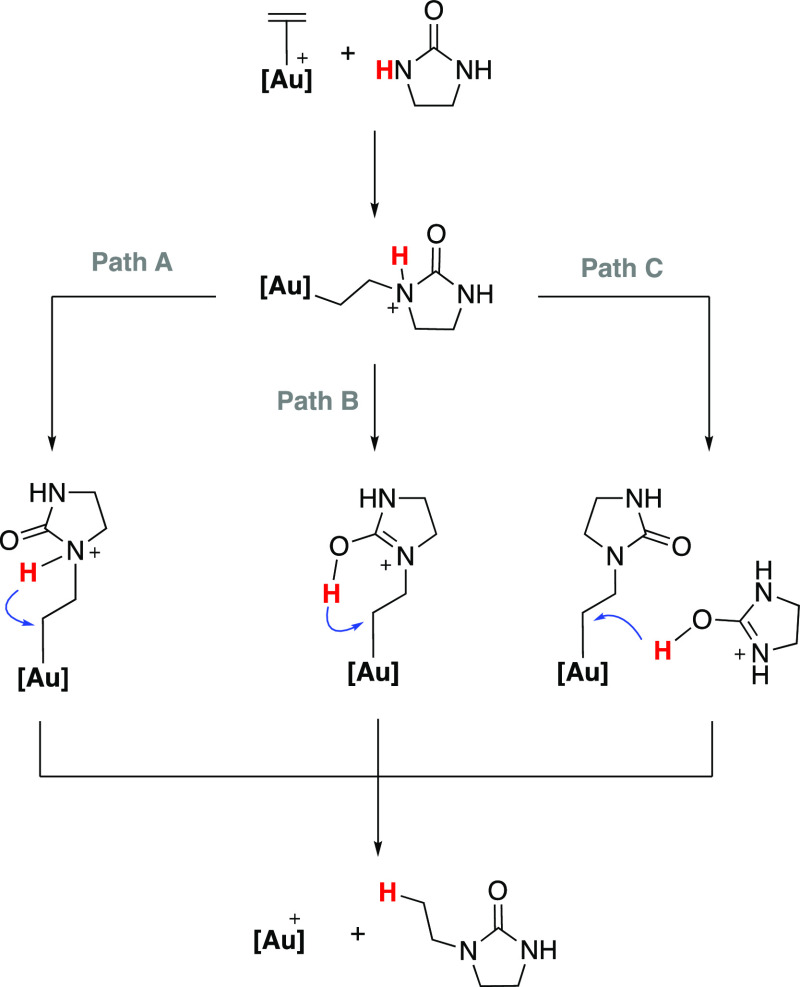
Schematic Representation of the Three Possible Mechanistic Pathways (Paths A–C) for the Protodeauration Step

As previously commented, chloride abstraction from the gold(I) chloride precatalyst generates the gold(I)–ethylene complex as the catalytically active species. Then, the nucleophilic addition of imidazolidine-2-one (**9** = Nu) to the electrophilic carbon–carbon double bond constitutes the first step of the process. This transformation presents a barrier of 18.7 kcal/mol (**TS1**) relative to the independently computed reactants (Figure 4). This reaction is an endergonic process, yielding **Int1** at 14.6 kcal/mol. From this step, we envisioned three possible mechanistic pathways, two in which the protodeauration step proceeds directly from the activated N-nucleophile **9** (intramolecular) and one assisted by a second molecule of imidazolidine-2-one (intermolecular) acting as a proton shuttle, as previously reported by Lledós and co-workers. These alternative intramolecular (paths A and B) and intermolecular (path C) routes are summarized in Scheme 3.

We have computationally explored the three potential routes depicted in Scheme 3, with two alternative scenarios for path B (*vide infra*). For the first intramolecular protodeauration process (path A, Figure S88), a direct proton transfer from the nitrogen to the coordinated carbon atom is proposed. An initial rearrangement through a rotation of the coordinated nucleophile (**TS2a**, 19.7 kcal/mol) is followed by the proton transfer from the nitrogen to the coordinated carbon atom. The transition state for the proton transfer (**TS3a**), which leads to the hydroaminated product **11**, was located at 45.9 kcal/mol (Figure S88). This barrier is too high to fit with our experimental observations. Alternatively, an intramolecular tautomerization through proton transfer from the nitrogen to the oxygen atom can be proposed, but the corresponding transition state (**TS2b** in Figure S90) is prohibitively high at 63.0 kcal/mol with respect to the separated reactants. Although the following proton transfer from the oxygen to the coordinated carbon atom (**TS3b**) drops to 23.8 kcal/mol (Figure S90), the overall kinetic barrier that accounts for 63.0 kcal/mol highly differs from our experimental results (experimentally we estimate an average Δ*G*_373K_ of around 26.8 kcal/mol; see the Supporting Information for details).

Because the protodeauration step in path B seems indeed feasible, we examined an alternative way to access the required O–H intermediate. More precisely, we examined the intermolecular tautomerization process previously proposed by Lledós^10g,11b^ and also related to the palladium intramolecular hydroamination of alkenes.^34^ The intermolecular proton transfer from the nitrogen atom to the oxygen atom of a second molecule of the imidazolidine-2-one occurs in a barrierless fashion, leading to **Int2b′** at 18.6 kcal/mol with respect to the separated reactants.^35^ In contrast to the intramolecular scenario in path B (**TS2b**, 63.0 kcal/mol), the intermolecular tautomerization process through a proton transfer to a second molecule of **9** presents a negligible energy barrier (**TS2b′**, Figure S93). Then, the intramolecular proton transfer to the coordinated carbon atom (**TS3b** = **TS3b′**, at 23.8 kcal/mol) leads to the final hydroaminated product **11** (Figure S93, path B′).

Instead of mediating the above tautomerization, the protonated molecule of **9** may directly affect the intermolecular protodeauration as depicted in path C. We have also computed this route. The proton transfer from the protonated nucleophile (Nu–H) can occur directly to the coordinated carbon atom (**TS2c**, at 28.3 kcal/mol) leading to the final product **11** (Figure 4). This energy barrier (28.3 kcal/mol) is relatively higher than the one found for the intramolecular proton transfer from the oxygen to the coordinated carbon (**TS3b′** in path B′), but it also fits reasonably well with the overall value measured experimentally (Δ*G*_373K_ ≈ 26.8 kcal/mol). These studies indicate that the two intramolecular processes (paths A and B) present unfeasibly overall high energy barriers of 45.9 and 63.0 kcal/mol and that a second molecule of imidazolidine-2-one is required as a proton shuttle to mediate the subsequent intramolecular (path B′) or intermolecular (path C) protodeauration step. In any case, it is interesting to note that despite its extreme bulkiness, the flexibility of ligand **L1** permits the accommodation of a second molecule of the amide in the cavity generated around the gold center, allowing the catalytic process.

**Figure 5:**
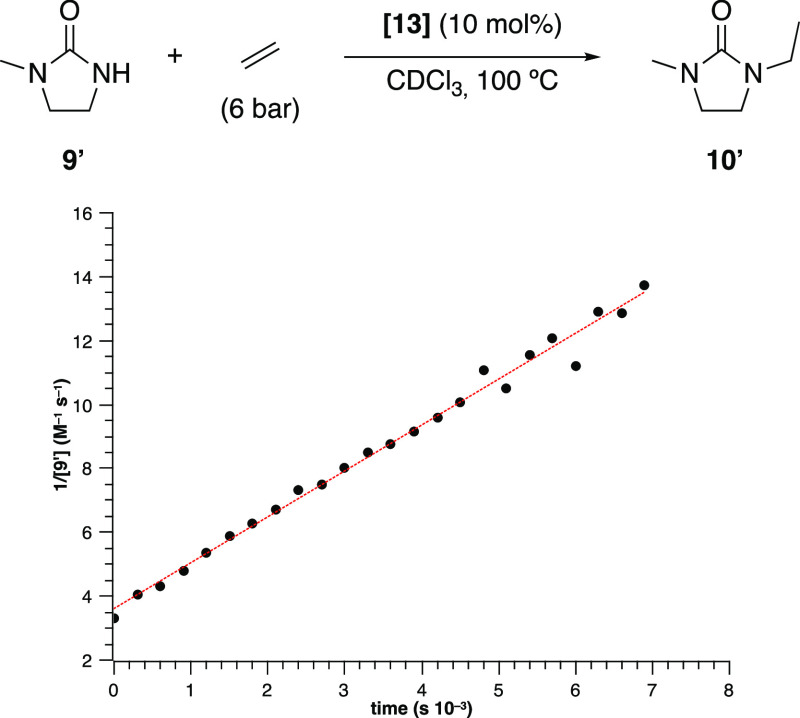
Second-order kinetic representation of the consumption of 1-methyl-imidazolidine-2-one at 100 °C in CDCl_3_ under 6 bar of ethylene (*k*_obs_ = 1.43 ± 0.03 × 10^–3^ M^–1^ s^–1^).

To further support the intermolecular-assisted mechanism and to differentiate between the two aforementioned and close-in-energy potential routes, we studied experimentally the hydroamination of ethylene by 1-methyl-imidazolidine-2-one **9′** catalyzed by complex **13**. It is worth noting that complex **13** was employed instead of complex **1** to avoid any potential complication associated with the presence of silver salts. In an initial experiment, we monitored by ^1^H NMR spectroscopy the conversion of 1-methyl-imidazolidine-2-one (0.3 M) under catalytic conditions of 10 mol % of complex **13** (0.03 M) at 100 °C in CDCl_3_ under 6 bar of ethylene pressure (0.8 M). A plot of 1/[**9′**] vs time was linear with a pseudo-second-order rate constant of 1.43 ± 0.03 × 10^–3^ M^–1^ s^–1^, indicating a pseudo-second-order dependence of the rate of the reaction on the concentration of 1-methyl-imdazolidine-2-one (Figure 5). This and subsequent kinetic experiments to be discussed were run in duplicate or triplicate in all cases. In addition, ^1^H and ^31^P NMR spectra of the catalytic reactions (Figures S78–S80) showed that the only gold species detected during the course of the catalysis, the one acting as the resting state, is the gold–ethylene complex **1**·**C_2_H_4_**, in accordance with the potential profiles investigated by DFT (Figures 4 and S93). This indicates the key role of the gold(I)–ethylene adduct in the gold(I)-catalyzed hydroamination of ethylene, in agreement with the benefits derived from using ligands that stabilize this unusual resting state.

**Figure 6:**
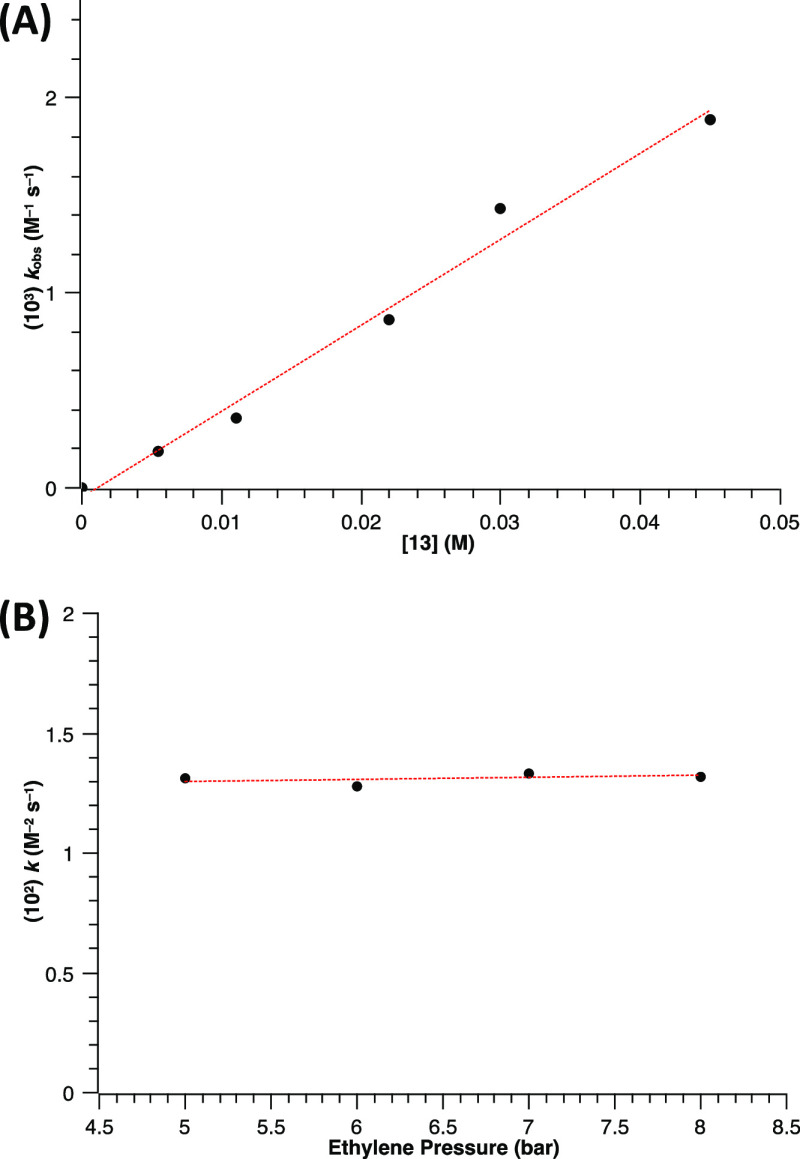
(A) Plot of pseudo-second-order rate constants vs catalyst concentration for the hydroamination of **9′** with ethylene (6 bar) catalyzed by complex **13** (0.0055–0.045 M) in CDCl_3_ at 100 °C. (B) Plot of pseudo-third-order (*k* = *k*_obs_/[**13**]) vs ethylene pressure for the hydroamination of **9′** with ethylene (5–8 bar) catalyzed by complex **13** (0.03 M) in CDCl_3_ at 60 °C.

To determine the dependence of the rate of the hydroamination reaction on catalyst concentration, pseudo-second-order rate constants were determined for the gold(I)-catalyzed hydroamination of ethylene (6 bar) with **9′** (0.3 M) as a function of complex **13** from 0.055 to 0.450 M at 100 °C, which established a first-order dependence of the rate on catalyst concentration (Figure 6A). Likewise, to determine the dependence of the rate of hydroamination on ethylene pressure, pseudo-second-order rate constants were determined for the reaction of **9′** (0.2 M) with ethylene catalyzed by complex **13** (15 mol %, 0.03 M) as a function of ethylene pressure from 4 to 8 bar at 60 °C. A plot of the corresponding pseudo-second-order rate constants vs the ethylene pressure was almost flat, which establishes a zero-order dependence on ethylene concentration (Figure 6B). To differentiate between the two potential mechanisms proposed above, that is, the intramolecular (path B′, Figure S93) and the intermolecular (path C, Figure 4) protodeauration, we derived their corresponding differential equations (see Schemes S1 and S2 in the Supporting Information). While both alternative pathways agree with a zero-order dependence on ethylene and first-order dependence on the catalyst, only the route depicted in Figure 4 (path C) is in agreement with a second-order dependence on the nucleophile. Thus, we proposed the hydroamination of ethylene to proceed through that pathway.

To gain more insight into the proposed involvement of a proton transfer process in the turnover-limiting step during gold-catalyzed hydroamination, we evaluated the kinetic isotope effect (KIE) resulting from the deuteroamination of ethylene with the deuterated 1-methyl-imdazolidine-2-one (**9′**-*d*_1_). The corresponding plot of 1/[**9′**-*d*_1_] vs time was linear with a pseudo-second-order constant of 4.56 ± 0.02 × 10^–4^ M^–1^ s^–1^. Comparison of the pseudo-second-order constant determined for the hydroamination of ethylene with 1-methyl-imdazolidine-2-one gave a significant primary deuterium KIE of *k*_H_/*k*_D_ = 3.14 (Figure S81), which corroborates the involvement of H-containing bond-breaking processes during the rate-limiting step of the catalytic reaction, which is attributed to intermolecular protodeauration.

## Conclusions

In summary, we have synthesized and structurally characterized a family of highly unusual dicoordinate gold(I)–ethylene complexes bearing phosphine ligands with variable bulkiness. The use of bulky phosphines is crucial to stabilize the gold(I)–ethylene bond and prevent catalyst decomposition, two key aspects for catalytic performance. In fact, while there is no apparent decomposition for the more sterically hindered complex **1**·**C_2_H_4_**, slow decomposition of complexes **2**–**8**·**C_2_H_4_** either in solution or in the solid state is detected. Interestingly, X-ray diffraction revealed a nonsymmetric coordination of ethylene at gold(I) with a slipped η^2^-coordination for complex **8**·**C_2_H_4_**, further suggesting the lability of this type of coordination. Complexes **1**–**8** have been tested as precatalysts for the underdeveloped Au(I)-catalyzed hydroamination of ethylene. Precatalysts bearing the most sterically demanding phosphine **1** showed the best results achieving full conversion within 18 h under only 1 bar of ethylene pressure at 60 °C, highlighting the high catalytic potential of very sterically crowded catalysts. On the other hand, complexes with smaller phosphine ligands afforded little or no conversion in this transformation. Kinetic analysis together with DFT calculations shows that the preferred mechanistic pathway involves the assistance of a second molecule of the nucleophile even when using the more sterically congested cavity-shaped complex **1**. In addition, a strong primary KIE has been observed, corroborating the involvement of H-containing bond-breaking processes in the rate-limiting step of the catalytic transformation that we attribute to intermolecular protodeauration.

## Experimental Section

### General Considerations

**Figure 7:**
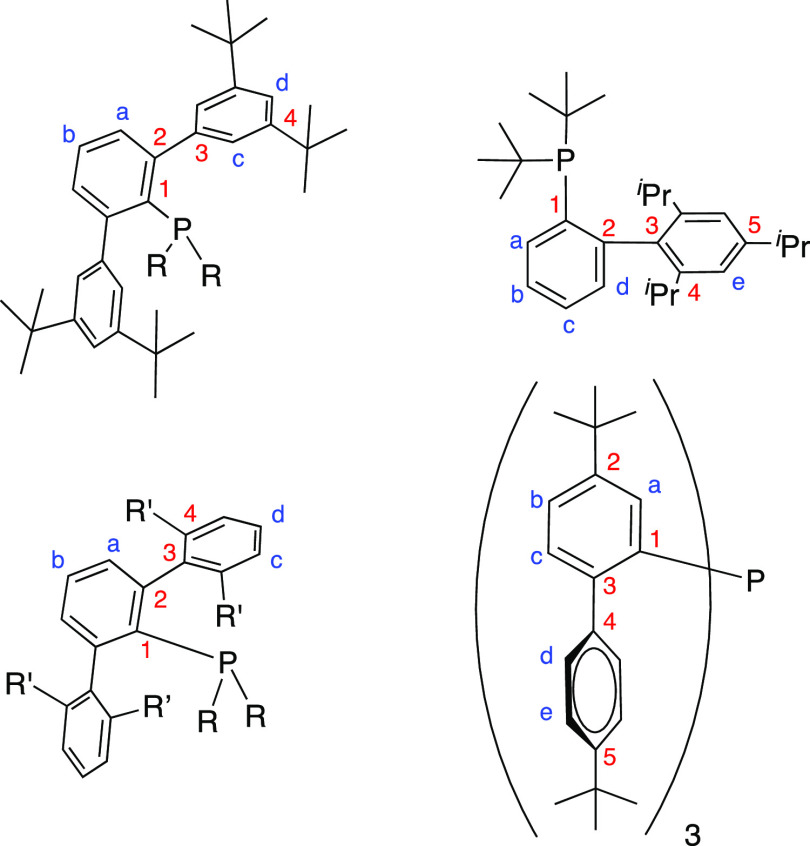
Labeling scheme used for ^1^H and ^13^C{^1^H} NMR assignments.

Unless otherwise stated, all reactions and manipulations were carried out under an atmosphere of dry argon or nitrogen using standard Schlenk techniques or in a nitrogen glovebox. Solvents were distilled under an inert atmosphere prior to use. Solution ^1^H, ^13^C, and ^31^P NMR spectra were recorded on Bruker AMX-300, DRX-400, and DRX-500 spectrometers at 298 K unless otherwise stated. Chemical shifts (δ) are expressed with a positive sign, in parts per million. ^1^H and ^13^C chemical shifts reported are referenced internally to residual protio (^1^H) or deutero (^13^C) solvent, while ^31^P chemical shifts are relative to 85% H_3_PO_4_. The following abbreviations and their combinations are used: br, broad; s, singlet; d, doublet; t, triplet; m, multiplet. The ^1^H and ^13^C resonance signals were attributed by means of 2D HSQC and HMBC experiments (Figure 7). For elemental analyses, a LECO TruSpec CHN elementary analyzer was utilized. [AuCl(THT)]^36^ (THT = tetrahydrothiophene) and all used phosphines (**L1**–**L8**)^18,24^ were prepared according to literature procedures. All other reagents were used as received from commercial suppliers.

### General Synthesis of Gold(I) Chloride Complexes

A solution of the corresponding phosphine (0.470 mmol) in toluene (10 mL) was added over a suspension of [AuCl(THT)] (150 mg, 0.470 mmol) in toluene (5 mL) at 0 °C. The initial white suspension was stirred for 12 h at rt until it became a clear solution. The solvent was removed under vacuum, and the resulting colorless solid was washed with pentane and dried to give the corresponding gold chloride complexes. Complexes **1**–**6** have been previously reported.^19,21,23b^

#### Compound **7**

Complex **7** was prepared following the general procedure from **L7** (221 mg, 63%). Crystals suitable for X-ray diffraction were grown by slow evaporation of pentane into a dichloromethane solution of complex **7** at −32 °C. ^1^H NMR (300 MHz, C_6_D_6_, 25 °C) δ: 7.61 (s, 2H, H_d_), 7.58 (s, 4H, H_c_), 7.19–7.02 (m, 3H, H_a_ + H_b_), 1.43 (s, 36H, CH_3(*t*Bu)_), 0.50 (d, 6H, ^2^*J*_HP_ = 10.0 Hz, PCH_3_). ^13^C{^1^H} NMR (100 MHz, C_6_D_6_, 25 °C) δ: 152.3 (C_4_), 148.7 (d, ^2^*J*_CP_ = 8 Hz, C_3_), 141.8 (s, C_2_), 131.3 (d, ^3^*J*_PC_ = 7 Hz, CH_a_), 130.1 (s, CH_b_), 129.4 (d, ^1^*J*_PC_ = 55 Hz, C_1_), 124.9 (s, CH_c_) 122.9 (s, CH_d_), 35.3 (s, C_(*t*Bu)_), 31.8 (s, CH_3(*t*Bu)_), 17.6 (d, ^1^*J*_CP_ = 40 Hz, PCH_3_). ^31^P{^1^H} NMR (202 MHz, C_6_D_6_, 25 °C) δ: 0.4.

#### Compound **8**

Complex **8** was prepared following the general procedure from **L8** (295 mg, 71%). Crystals suitable for X-ray diffraction were grown by slow evaporation of a concentrated dichloromethane solution of complex **8**. Anal. calcd for C_46_H_67_AuClP: C, 62.54; H, 7.64. Found: C, 62.36; H, 7.27. ^1^H NMR (300 MHz, CD_2_Cl_2_, 25 °C) δ: 7.56 (d, 2H, ^4^*J*_HH_ = 1.7 Hz, CH_d_), 7.45 (td, 1H, ^3^*J*_HH_ = 7.5 Hz, ^5^*J*_HP_ = 1.6 Hz, CH_b_), 7.24 (dd, 2H, ^3^*J*_HH_ = 7.5 Hz, ^4^*J*_HP_ = 3.0 Hz, CH_a_), 7.06 (d, 4H, ^4^*J*_HH_ = 1.8 Hz, CH_c_), 2.12–1.95 (m, 2H, CH_(Cy)_), 1.78–1.55 (m, 10H, CH_2(Cy)_), 1.52–1.45 (m, 2H, CH_(Cy)_), 1.40 (s, 36H, CH_3(*t*Bu)_), 1.35–1.25 (m, 4H, CH_2(Cy)_), 1.19–1.11 (m, 2H, CH_2(Cy)_). 1.10–0.99 (m, 2H, CH_2(Cy)_). ^13^C{^1^H} NMR (100 MHz, CD_2_Cl_2_, 25 °C) δ: 151.3 (s, C_4_), 150.6 (s, C_2_), 142.9 (d, ^3^*J*_PC_ = 5 Hz, C_3_), 133.4 (d, ^3^*J*_PC_ = 7 Hz, CH_a_), 129.8 (s, CH_b_), 124.5 (s, CH_c_), 124.3 (d, ^1^*J*_PC_ = 48 Hz, C_1_), 123.2 (CH_c_), 37.8 (d, ^1^*J*_PC_ = 31 Hz, CH_(Cy)_), 35.7 (s, C_(*t*Bu)_), 34.6 (d, ^3^*J*_PC_ = 6 Hz, CH_2(Cy)_), 32.1 (s, CH_3(*t*Bu)_), 31.7 (s, CH_2(Cy)_), 27.1 (d, ^2^*J*_PC_ = 13 Hz, CH_2(Cy)_), 26.8 (d, ^2^*J*_PC_ = 15 Hz, CH_2(Cy)_), 26.3 (s, CH_2(Cy)_). ^31^P{^1^H} NMR (162 MHz, CD_2_Cl_2_, 25 °C) δ: 48.8.

### General Synthesis of Gold(I)–Ethylene Complexes

In a glovebox, a Schlenk flask was charged with silver hexafluoroantimonate (8 mg, 0.022 mmol) in dichloromethane (1 mL). The corresponding gold(I) chloride complex (0.02 mmol) was transferred into a small glass vial and dissolved in dichloromethane (1 mL). The vial solution was loaded into a plastic syringe equipped with a stainless steel needle. Outside the glovebox, the Schlenk flask was cooled down to −30 °C. At this temperature, the solution of the gold(I) chloride complex was added to the AgSbF_6_ suspension while bubbling ethylene. The mixture was allowed to slowly warm up to room temperature and filtered through a short pad of Celite to remove the silver salts, and the solvent was removed under vacuum affording the corresponding gold(I)-ethylene complexes as colorless solids. Complex **1**·**C_2_H_4_** has been previously reported.^19^

#### Compound **2**·**C_2_H_4_**

Complex **2**·**C_2_H_4_** was prepared following the general procedure from gold(I) chloride complex **2** (13 mg, 78%). Anal. calcd for C_29_H_37_AuF_6_PSb: C, 41.01; H, 4.39. Found: C, 41.08; H, 4.54. ^1^H NMR (400 MHz, CD_2_Cl_2_, 25 °C) δ: 7.01 (bs, 6H, m-CH), 5.46 (bs, 4H, CH_2(C2H4)_), 2.66 (bs, 9H, o-CH_3_), 2.36 (s, 9H, p-CH_3_), 1.86 (bs, 9H, o-CH_3_). ^13^C{^1^H} NMR (100 MHz, CD_2_Cl_2_, 25 °C) δ: 143.6 (s, p-C), 143.0 (bs, o-C), 132.9 (bs, m-CH), 123.3 (d, ^1^*J*_CP_ = 56 Hz, C), 111.2 (d, ^2^*J*_CP_ = 9 Hz, C_(C2H4)_), 24.4 (bs, o-CH_3_), 21.3 (s, p-CH_3_). ^31^P{^1^H} NMR (162 MHz, CD_2_Cl_2_, 25 °C) δ: 1.5. ^1^H NMR (400 MHz, CD_2_Cl_2_, −30 °C) δ:7.06 (s, 3H, m-CH), 6.90 (s, 3H, m-CH), 5.43 (bs, 4H, CH_2(C2H4)_), 2.64 (s, 9H, o-CH_3_), 2.31 (s, 9H, p-CH_3_), 1.78 (s, 9H, o-CH_3_). ^13^C{^1^H} NMR (100 MHz, CD_2_Cl_2_, −30 °C) δ: 143.3 (d, ^3^*J*_CP_ = 5 Hz, o-C), 143.0 (s, p-C), 142.2 (d, ^3^*J*_CP_ = 16 Hz, o-C), 133.0 (d, ^3^*J*_CP_ = 9 Hz, m-CH), 132.0 (d, ^4^*J*_CP_ = 10 Hz, m-CH), 122.6 (bs, CH_2(C2H4)_), 122.5 (d, ^1^*J*_CP_ = 56 Hz, C), 24.9 (d, ^3^*J*_CP_ = 17 Hz, o-CH_3_), 24.0 (d, ^3^*J*_CP_ = 5 Hz, o-CH_3_), 21.0 (s, p-CH_3_). ^1^H NMR (400 MHz, CD_2_Cl_2_, −70 °C) δ: 7.03 (d, 6H, ^4^*J*_HP_ = 5.0 Hz, m-CH), 6.87 (s, 6H, m-CH), 5.67 (d, 4H, ^3^*J*_HP_ = 2.9 Hz, CH_2(C2H4)_), 2.60 (s, 9H, o-CH_3_), 2.28 (s, 9H, p-CH_3_), 1.72 (s, 9H, o-CH_3_).

#### Compound **3**·**C_2_H_4_**

Complex **3**·**C_2_H_4_** was prepared following the general procedure from gold(I) chloride complex **3** (16 mg, 89%). Crystals suitable for X-ray diffraction were grown by slow evaporation of pentane into a dichloromethane solution of complex **3**·**C_2_H_4_**. Anal. calcd for C_31_H_49_AuF_6_PSb: C, 42.05; H, 5.58. Found: C, 41.83; H, 5.89. ^1^H NMR (400 MHz, CD_2_Cl_2_, 25 °C) δ: 7.90 (m, 1H, CH_d_), 7.64 (m, 2H, CH_b_ + CH_c_), 7.29 (s, 2H, CH_e_), 7.23 (m, 1H, CH), 4.95 (d, 4H, ^3^*J*_HP_ = 2.6 Hz, CH_2(C2H4)_), 3.04 (hept, 1H, ^3^*J*_HH_ = 6.9 Hz, CH_(*i*Pr)_), 2.35 (hept, 2H, ^3^*J*_HH_ = 6.9 Hz, CH_(*i*Pr)_), 1.45 (d, 18H, ^3^*J*_HP_ = 16.5 Hz, CH_3(*t*Bu)_), 1.35 (d, 6H, ^3^*J*_HH_ = 6.9 Hz, CH_3(*i*Pr)_), 1.24 (d, 6H, ^3^*J*_HH_ = 6.9 Hz, CH_3(*i*Pr)_), 0.94 (d, 6H, ^3^*J*_HH_ = 6.9 Hz, CH_3(*i*Pr)_). ^13^C{^1^H} NMR (100 MHz, CD_2_Cl_2_, 25 °C) δ: 151.9 (s, C_5_), 149.0 (s, C_4_), 146.8 (d, ^2^*J*_CP_ = 15 Hz, C_2_), 136.9 (d, ^3^*J*_CP_ = 7 Hz, C_3_), 135.8 (d, ^3^*J*_CP_ = 3 Hz, CH_d_), 135.4 (d, ^2^*J*_CP_ = 8 Hz, CH_a_), 132.5 (d, ^4^*J*_CP_ = 2 Hz, CH_c_), 128.7 (d, ^3^*J*_CP_ = 7 Hz, CH_b_), 128.4 (d, ^1^*J*_CP_ = 45 Hz, C_1_), 123.5 (s, CH_e_), 110.9 (d, ^2^*J*_CP_ = 8 Hz, C_(C2H4)_), 39.9 (d, ^1^*J*_CP_ = 24 Hz, C_(*t*Bu)_), 34.7 (s, p-CH_(*i*Pr)_), 31.6 (s, o-CH_(*i*Pr)_), 31.6 (s, CH_3(*t*Bu)_) 26.0 (s, CH_3(*i*Pr)_), 24.6 (s, CH_3(*i*Pr)_), 23.8 (s, CH_3(*i*Pr)_). ^31^P{^1^H} NMR (162 MHz, CD_2_Cl_2_, 25 °C) δ: 65.6.

#### Compound **4**·**C_2_H_4_**

Complex **4**·**C_2_H_4_** was prepared following the general procedure from gold(I) chloride complex **4** (13 mg, 71%). Anal. calcd for C_34_H_47_AuF_6_PSb: C, 44.42; H, 5.15. Found: C, 44.48; H, 5.31. ^1^H NMR (400 MHz, CD_2_Cl_2_, 25 °C) δ: 7.64 (td, 1H, ^3^*J*_HH_ = 7.6 Hz, ^5^*J*_HP_ = 1.9 Hz, CH_b_), 7.55 (t, 2H, ^3^*J*_HH_ = 7.3 Hz, CH_d_), 7.40 (d, 4H, ^3^*J*_HH_ = 7.3 Hz, CH_c_), 7.26 (dd, 2H, ^3^*J*_HH_ = 7.6 Hz, ^4^*J*_HP_ = 3.7 Hz, CH_a_), 4.85 (d, 4H, ^3^*J*_HP_ = 3.0 Hz, CH_2(C2H4)_), 2.45 (hept, ^3^*J*_HH_ = 6.8 Hz, 4H, CH_(*i*Pr)_), 1.46 (d, ^3^*J*_HP_ = 10.7 Hz, 6H, PCH_3_), 1.31 (d, 2H, ^3^*J*_HH_ = 6.8 Hz, 12H, CH_3(*i*Pr)_), 1.06 (d, 2H, ^3^*J*_HH_ = 6.8 Hz, 12H, CH_3(*i*Pr)_). ^13^C{^1^H} NMR (100 MHz, CD_2_Cl_2_, 25 °C) δ: 147.8 (s, C_4_), 146.2 (d, ^2^*J*_PC_ = 12 Hz, C_2_), 138.2 (d, ^3^*J*_PC_ = 6 Hz, C_3_), 133.7 (d, ^3^*J*_PC_ = 8 Hz, CH_a_), 132.0 (s, CH_b_), 130.4 (s, CH_d_), 127.9 (d, ^1^*J*_CP_ = 60 Hz C1), 124.6 (s, CH_c_), 110.3 (d, ^2^*J*_CP_ = 9 Hz, CH_2(C2H4)_), 32.0 (s, CH_(*i*Pr)_), 25.6 (s, CH_3(*i*Pr)_), 23.2 (s, CH_3(*i*Pr)_), 16.2 (d, ^2^*J*_CP_ = 37 Hz, PCH_3_). ^31^P{^1^H} NMR (162 MHz, CD_2_Cl_2_, 25 °C) δ: 4.3.

#### Compound **5**·**C_2_H_4_**

Complex **5**·**C_2_H_4_** was prepared following the general procedure from gold(I) chloride complex **5** (9 mg, 53%). ^1^H NMR (400 MHz, CD_2_Cl_2_, 25 °C) δ: 7.72 (td, 1H, ^3^*J*_HH_ = 7.6 Hz, ^5^*J*_HP_ = 1.9 Hz, CH_b_), 7.36 (t, 2H, ^3^*J*_HH_ = 7.3 Hz, CH_d_), 7.28 (d, 4H, ^3^*J*_HH_ = 7.3 Hz, CH_c_), 7.15 (dd, 2H, ^3^*J*_HH_ = 7.6 Hz, ^4^*J*_HP_ = 3.6 Hz, CH_a_), 5.00 (bs, 4H, CH_2(C2H4)_), 2.04 (s, 12H, CH_3(Xyl)_), 1.49 (d, 2H, ^2^*J*_HP_ = 10.4 Hz, PCH_3_). ^13^C{^1^H} NMR (100 MHz, CD_2_Cl_2_, 25 °C) δ: 147.8 (d, ^2^*J*_PC_ = 13 Hz, C_2_), 140.7 (s, C_3_), 137.3 (s, C_4_), 134.3 (s, CH_b_), 132.4 (d, ^3^*J*_PC_ = 8 Hz, CH_a_), 129.3 (s, CH_d_), 129.0 (s, CH_c_), 125.6 (s, C_1_), 111.5 (s, CH_2(C2H4)_), 21.8 (s, CH_3(Xyl)_), 16.0 (d, ^2^*J*_CP_ = 37 Hz, PCH_3_). ^31^P{^1^H} NMR (162 MHz, CD_2_Cl_2_, 25 °C) δ: 4.1. ^1^H NMR (400 MHz, CD_2_Cl_2_, −30 °C) δ: 7.72 (td, 1H, ^3^*J*_HH_ = 7.6 Hz, ^5^*J*_HP_ = 1.9 Hz, CH_b_), 7.37 (t, 2H, ^3^*J*_HH_ = 7.5 Hz, CH_d_), 7.28 (d, 4H, ^3^*J*_HH_ = 7.5 Hz, CH_c_), 7.14 (dd, 2H, ^3^*J*_HH_ = 7.6 Hz, ^4^*J*_HP_ = 3.7 Hz, CH_a_), 5.36 (bs, 4H, CH_2(C2H4)_), 2.02 (s, 12H, CH_3(Xyl)_), 1.48 (d, 2H, ^2^*J*_HP_ = 10.7 Hz, PCH_3_). ^13^C{^1^H} NMR (100 MHz, CD_2_Cl_2_, −30 °C) δ: 147.1 (d, ^2^*J*_PC_ = 12 Hz, C_2_), 140.1 (d, ^3^*J*_PC_ = 7 Hz, C_3_), 136.9 (s, C_4_), 134.0 (s, CH_b_), 131.8 (d, ^3^*J*_PC_ = 7 Hz, CH_a_), 128.9 (s, CH_d_), 128.5 (s, CH_c_), 125.1 (d, ^1^*J*_PC_ = 62 Hz, C_1_), 120.9 (bs, CH_2(C2H4)_), 21.6 (s, CH_3(Xyl)_), 15.6 (d, ^2^*J*_CP_ = 37 Hz, PCH_3_).

#### Compound **6**·**C_2_H_4_**

Complex **6**·**C_2_H_4_** was prepared following the general procedure from gold(I) chloride complex **6** (16 mg, 87%). Anal. calcd for C_34_H_43_AuF_6_PSb: C, 44.61; H, 4.73. Found: C, 44.68; H, 4.99. ^1^H NMR (400 MHz, CD_2_Cl_2_, 25 °C) δ: 7.69 (td, 1H, ^3^*J*_HH_ = 7.6 Hz, ^5^*J*_HP_ = 1.8 Hz, CH_b_), 7.40–7.22 (m, 6H, CH_d_ + CH_c_), 7.21–7.13 (m, 2H, CH_a_), 4.86 (d, 4H, ^3^*J*_HP_ = 2.8 Hz, CH_2(C2H4)_), 2.45–2.28 (m, 2H, CH_(Cy)_), 2.08 (s, 12H, CH_3(Xyl)_), 1.93–1.85 (m, 2H, CH_2(Cyp)_), 1.76–1.57 (m, 6H, CH_2(Cyp)_), 1.56–1.43 (m, 4H, CH_2(Cyp)_), 1.40–1.19 (m, 4H, CH_2(Cyp)_). ^13^C{^1^H} NMR (100 MHz, CD_2_Cl_2_, 25 °C) δ: 148.2 (d, ^2^*J*_PC_ = 10 Hz, C_2_), 140.9 (bs, C_3_), 137.8 (s, C_4_), 133.4 (s, CH_a_), 133.2 (s, CH_b_), 129.2 (s, CH_c_), 128.7 (s, CH_d_), 128.7 (d, ^1^*J*_PC_ = 50 Hz, C_1_), 111.0 (d, ^2^*J*_CP_ = 9 Hz, CH_2(C2H4)_), 38.3 (d, ^1^*J*_PC_ = 31 Hz, CH_(Cyp)_), 36.3 (d, ^3^*J*_PC_ = 8 Hz, CH_2(Cyp)_), 32.8 (d, ^3^*J*_PC_ = 6 Hz, CH_2(Cyp)_), 25.8 (d, ^2^*J*_PC_ = 12 Hz, CH_2(Cyp)_), 25.6 (d, ^2^*J*_PC_ = 14 Hz, CH_2(Cyp)_), 21.9 (s, CH_3(Xyl)_). ^31^P{1H} NMR (162 MHz, CD_2_Cl_2_, 25 °C) δ: 57.6. ^1^H NMR (400 MHz, CD_2_Cl_2_, −30 °C) δ: 7.71 (td, 1H, ^3^*J*_HH_ = 7.6 Hz, ^5^*J*_HP_ = 1.9 Hz, CH_b_), 7.49–7.38 (m, 2H, CH_d_), 7.34–7.23 (m, 2H, CH_c_), 7.23–7.17 (m, 2H, CH_c_), 7.11–7.04 (m, 2H, CH_a_), 4.86 (d, 4H, ^3^*J*_HP_ = 2.8 Hz, CH_2(C2H4)_), 2.43–2.25 (m, 2H, CH_(Cy)_), 2.08 (s, 12H, CH_3(Xyl)_), 1.94–1.84 (m, 2H, CH_2(Cyp)_), 1.79–1.45 (m, 10H, CH_2(Cyp)_), 1.40–1.14 (m, 4H, CH_2(Cyp)_). ^13^C{^1^H} NMR (100 MHz, CD_2_Cl_2_, −30 °C) δ: 147.9 (s, C_2_), 147.3 (d, ^2^*J*_PC_ = 19 Hz, C_2_), 141.8 (s, C_3_), 139.1 (s, C_3_), 137.5 (s, C_4_), 137.3 (s, C_4_), 133.2 (d, ^4^*J*_PC_ = 7 Hz, CH_a_), 133.1 (s, CH_b_), 132.0 (d, ^4^*J*_PC_ = 7 Hz, CH_a_), 129.5 (s, CH_c_), 129.2 (s, CH_d_), 128.8 (d, ^1^*J*_PC_ = 46 Hz, C_1_), 127.7 (s, CH_c_), 111.0 (d, ^2^*J*_CP_ = 9 Hz, CH_2(C2H4)_), 37.6 (d, ^1^*J*_PC_ = 32 Hz, CH_(Cyp)_), 35.9 (d, ^3^*J*_PC_ = 8 Hz, CH_2(Cyp)_), 32.3 (d, ^3^*J*_PC_ = 6.5 Hz, CH_2(Cyp)_), 25.2 (d, ^2^*J*_PC_ = 12 Hz, CH_2(Cyp)_), 25.1 (d, ^2^*J*_PC_ = 14 Hz, CH_2(Cyp)_), 21.8 (s, CH_3(Xyl)_), 21.3 (s, CH_3(Xyl)_).

#### Compound **7**·**C_2_H_4_**

Complex **7**·**C_2_H_4_** was prepared following the general procedure from gold(I) chloride complex **7** (12 mg, 63%). Anal. calcd for C_38_H_55_AuF_6_PSb: C, 46.79; H, 5.68. Found: C, 46.71; H, 5.94. ^1^H NMR (400 MHz, CD_2_Cl_2_, 25 °C) δ: 7.77–7.65 (m, 1H, CH_b_), 7.64 (t, 2H, ^3^*J*_HH_ = 1.7 Hz, CH_d_), 7.43 (dd, 2H, ^3^*J*_HH_ = 7.6 Hz, ^4^*J*_HP_ = 3.9 Hz, CH_a_), 7.25 (d, 4H, ^4^*J*_HH_ = 1.7 Hz, CH_c_), 5.16 (s, 4H, CH_2(C2H4)_), 1.60 (d, 2H, ^2^*J*_HP_ = 10.7 Hz, PCH_3_), 1.41 (s, 36H, CH_3(*t*Bu)_).^13^C{^1^H} NMR (100 MHz, CD_2_Cl_2_, 25 °C) δ: 152.6 (s, C_4_), 150.3 (s, ^2^*J*_CP_ = 11 Hz, C_3_), 141.1 (d, ^3^*J*_CP_ = 6 Hz, C_2_), 132.9 (d, ^3^*J*_CP_ = 8 Hz, CH_a_), 132.1 (s, CH_b_), 125.5 (d, ^1^*J*_PC_ = 60 Hz, C_1_), 125.0 (s, CH_d_), 123.5 (s, CH_d_), 111.8 (bs, CH_2(C2H4)_), 35.6 (s, C_(*t*Bu)_), 31.9 (s, CH_3(*t*Bu)_), 18.4 (d, ^2^*J*_CP_ = 37 Hz, PCH_3_). ^31^P{^1^H} NMR (162 MHz, CD_2_Cl_2_, 25 °C) δ: 9.4. ^1^H NMR (400 MHz, CD_2_Cl_2_, −30 °C) δ: 7.71 (td, 1H, ^3^*J*_HH_ = 7.7 Hz, ^5^*J*_HP_ = 1.7 Hz, CH_b_), 7.59 (t, 2H, ^3^*J*_HH_ = 1.7 Hz, CH_d_), 7.43 (dd, 2H, ^3^*J*_HH_ = 7.7 Hz, ^4^*J*_HP_ = 3.8 Hz, CH_a_), 7.22 (d, 4H, ^4^*J*_HH_ = 1.8 Hz, CH_c_), 5.35 (s, 4H, CH_2(C2H4)_), 1.56 (d, 2H, ^2^*J*_HP_ = 10.4, PCH_3_), 1.37 (s, 36H, CH_3(*t*Bu)_). ^13^C{^1^H} NMR (100 MHz, CD_2_Cl_2_, −30 °C) δ: 151.9 (s, C_4_), 149.9 (s, ^2^*J*_CP_ = 11 Hz, C_3_), 140.6 (s, ^3^*J*_CP_ = 7 Hz, C_2_), 132.3 (d, ^3^*J*_CP_ = 7 Hz, CH_a_), 131.7 (s, CH_b_), 125.0 (d, ^1^*J*_PC_ = 62 Hz, C_1_), 124.6 (s, CH_d_), 123.0 (s, CH_d_), 120.5 (s, CH_2(C2H4)_), 35.2 (s, C_(*t*Bu)_), 31.4 (s, CH_3(*t*Bu)_), 18.0 (d, ^2^*J*_CP_ = 37 Hz, PCH_3_).

#### Compound **8**·**C_2_H_4_**

Complex **8**·**C_2_H_4_** was prepared following the general procedure from gold(I) chloride complex **8** (21 mg, 93%). Crystals suitable for X-ray diffraction were grown by slow diffusion of pentane into a dichloromethane solution of complex **8**·**C_2_H_4_**. Anal. calcd for C_48_H_71_AuF_6_PSb: C, 51.86; H, 6.44. Found: C, 51.92; H, 6.64. ^1^H NMR (400 MHz, CD_2_Cl_2_, 25 °C) δ: 7.65 (s, 2H, CH_d_), 7.55 (td, 1H, ^3^*J*_HH_ = 7.7 Hz, ^5^*J*_HP_ = 1.8 Hz, CH_b_), 7.26 (bs, 2H, CH_a_), 7.13 (d, 4H, ^4^*J*_HH_ = 1.8 Hz, CH_c_), 4.77 (s, 4H, CH_2(C2H4)_), 2.37–2.21 (m, 2H, CH_(Cy)_), 1.89–1.65 (m, 10H, CH_2(Cy)_), 1.42 (s, 36H, CH_3(*t*Bu)_), 1.22–1.04 (m, 10H, CH_2(Cy)_). ^13^C{^1^H} NMR (100 MHz, CD_2_Cl_2_, 25 °C) δ: 152.8 (bs, C_4_), 149.9 (s, C_3_), 149.8 (s, C_2_), 133.9 (s, CH_a_), 131.4 (s, CH_b_), 123.9 (bs, CH_c_ + CH_d_), 122.8 (d, ^1^*J*_PC_ = 50 Hz, C_1_), 109.0 (s, CH_2(C2H4)_), 38.6 (d, ^1^*J*_PC_ = 28 Hz, CH_(Cy)_), 35.7 (s, C_(*t*Bu)_), 34.3 (d, ^3^*J*_PC_ = 6 Hz, CH_2(Cy)_), 31.9 (s, CH_3(*t*Bu)_), 31.9 (s, CH_2(Cy)_), 26.9 (d, ^2^*J*_PC_ = 16 Hz, CH_2(Cy)_), 26.7 (d, ^2^*J*_PC_ = 13 Hz, CH_2(Cy)_), 26.0 (s, CH_2(Cy)_). ^31^P{^1^H} NMR (162 MHz, CD_2_Cl_2_, 25 °C) δ: 55.4. ^1^H NMR (400 MHz, CD_2_Cl_2_, −30 °C) δ: 7.60 (s, 2H, CH_d_), 7.54 (td, 1H, ^3^*J*_HH_ = 7.6 Hz, ^5^*J*_HP_ = 1.8 Hz, CH_b_), 7.31 (d, 1H, ^3^*J*_HH_ = 7.6 Hz, CH_a_), 7.19 (dd, 1H, ^3^*J*_HH_ = 7.6 Hz, ^4^*J*_HP_ = 4.3 Hz, CH_a_), 7.10 (s, 2H, CH_c_), 7.08 (s, 2H, CH_c_), 4.69 (d, 4H, ^3^*J*_HP_ = 2.6 Hz, CH_2(C2H4)_), 2.26–2.07 (m, 2H, CH_(Cy)_), 1.84–1.58 (m, 10H, CH_2(Cy)_), 1.38 (s, 18H, CH_3(*t*Bu)_), 1.37 (s, 18H, CH_3(*t*Bu)_), 1.23–0.98 (m, 10H, CH_2(Cy)_). ^13^C{^1^H} NMR (100 MHz, CD_2_Cl_2_, −30 °C) δ: 152.3 (s, C_4_), 151.0 (s, C_4_), 149.1 (s, C_3_), 149.0 (s, C_2_), 142.7 (s, C_3_), 140.4 (s, C_2_), 133.6 (d, ^3^*J*_PC_ = 6 Hz, CH_a_), 133.2 (d, ^3^*J*_PC_ = 6 Hz, CH_a_), 131.0 (s, CH_b_), 124.3 (s, CH_c_),123.9 (s, CH_d_), 122.8 (s, CH_c_), 122.6 (d, ^1^*J*_PC_ = 50 Hz, C_1_), 109.0 (d, ^2^*J*_PC_ = 8 Hz, CH_2(C2H4)_), 37.2 (d, ^1^*J*_PC_ = 29 Hz, CH_(Cy)_), 35.5 (s, C_(*t*Bu)_), 35.2 (s, C_(*t*Bu)_), 33.9 (d, ^3^*J*_PC_ = 5 Hz, CH_2(Cy)_), 31.5 (s, CH_3(*t*Bu)_), 31.3 (s, CH_2(Cy)_), 26.3 (d, ^2^*J*_PC_ = 15 Hz, CH_2(Cy)_), 26.2 (d, ^2^*J*_PC_ = 15 Hz, CH_2(Cy)_), 25.5 (s, CH_2(Cy)_).

### Synthesis of Gold(I)–Amine Complex **12**

A solution of complex **1** (32 mg, 0.02 mmol) in dichloromethane (1 mL) in the presence of diisopropylamine (5 mL, 0.03 mmol) was added to a suspension of silver hexafluoroantimonate (12 mg, 0.03 mmol) in dichloromethane (1 mL) at rt. The mixture was stirred for 30 min and filtered through a short pad of Celite to remove the silver salts, and the solvent was removed under vacuum affording complex **12** as a colorless solid (24 mg, 87%). Crystals suitable for X-ray diffraction were grown by slow diffusion of pentane into a dichloromethane solution of complex **12**. ^1^H NMR (500 MHz, CD_2_Cl_2_, 25 °C) δ: 7.73 (d, 3H, ^3^*J*_HH_ = 8.1 Hz, CH_b_), 7.51–7.35 (m, 12H, CH_a_ + CH_c_), 7.23 (d, 6H, ^3^*J*_HH_ = 8.0 Hz, CH_e_), 6.88 (d, 6H, ^3^*J*_HH_ = 8.0 Hz, CH_d_), 2.73 (hept, 1H, ^3^*J*_HH_ = 6.3 Hz, CH_(*i*Pr)_), 2.16 (bs, 1H, CH_(*i*Pr)_), 1.26 (s, 27H, CH_3(tBu)_), 1.20 (s, 27H, CH_3(*t*Bu)_), 0.65 (d, 3H, ^3^*J*_HH_ = 6.3 Hz, CH_3(*i*Pr)_), 0.53 (d, 3H, ^3^*J*_HH_ = 6.3 Hz, CH_3(*i*Pr)_), 0.51 (d, 3H, ^3^*J*_HH_ = 6.3 Hz, CH_3(*i*Pr)_), 0.45 (d, 3H, ^3^*J*_HH_ = 6.3 Hz, CH_3(*i*Pr)_). ^13^C{^1^H} NMR (125 MHz, CD_2_Cl_2_, 25 °C) δ: 151.8 (d, ^3^*J*_CP_ = 8 Hz, C_2_), 151.4 (s, C_5_), 143.6 (d, ^2^*J*_CP_ = 15 Hz, C_3_), 138.7 (d, ^3^*J*_CP_ = 6 Hz, C_4_), 134.9 (d, ^3^*J*_CP_ = 10 Hz, CH_a_ or CH_c_), 133.9 (d, ^3^*J*_CP_ = 8 Hz, CH_a_ or CH_c_), 130.2 (s, CH_d_), 129.6 (d, ^4^*J*_CP_ = 3 Hz, CH_b_), 127.8 (d, ^1^*J*_CP_ = 62 Hz, C_1_), 126.0 (s, CH_e_), 49.5 (s, CH_(*i*Pr)_), 49.1 (s, CH_(*i*Pr)_), 35.3 (s, C_(*t*Bu)_), 35.1 (s, C_(*t*Bu)_), 31.6 (s, CH_3(*t*Bu)_), 31.3 (s, CH_3(*t*Bu)_), 25.6 (s, CH_3(*i*Pr)_), 23.8 (s, CH_3(*i*Pr)_). ^31^P{^1^H} NMR (202 MHz, CD_2_Cl_2_, 25 °C) δ: 11.1.

### Synthesis of Gold(I)–Imidazolidine-2-one Complex **13**

A solution of complex **1** (106 mg, 0.10 mmol) in dichloromethane (1 mL) in the presence of imidazolidine-2-one (9 mg, 0.10 mmol) was added to a suspension of silver hexafluoroantimonate (38 mg, 0.11 mmol) in dichloromethane (1 mL) at rt. The mixture was stirred for 30 min and filtered through a short pad of Celite to remove the silver salts, and the solvent was removed under vacuum affording complex **13** as a white colorless solid (122 mg, 91%). Crystals suitable for X-ray diffraction were grown by slow diffusion of pentane into a dichloromethane solution of complex **13**. ^1^H NMR (400 MHz, CD_2_Cl_2_, 25 °C) δ: 7.70 (dd, 3H, ^3^*J*_HH_ = 8.1 Hz, ^4^*J*_HH_ = 1.8 Hz, CH_b_), 7.45–7.35 (m, 12H, CH_a_ + CH_c_), 7.15 (d, 6H, ^3^*J*_HH_ = 7.8 Hz, CH_e_), 6.68 (d, 6H, ^3^*J*_HH_ = 7.8 Hz, CH_d_), 4.14 (bs, 2H, NH), 3.58 (s, 4H, CH_2_), 1.25 (s, 27H, CH_3(tBu)_), 1.24 (s, 27H, CH_3(*t*Bu)_). ^13^C{^1^H} NMR (100 MHz, CD_2_Cl_2_, 25 °C) δ: 166.9 (CO), 151.9 (s, C_5_), 151.5 (d, ^3^*J*_CP_ = 8 Hz, C_2_), 144.4 (d, ^2^*J*_CP_ = 16 Hz, C_3_), 138.4 (d, ^3^*J*_CP_ = 7 Hz, C_4_), 133.1 (d, ^3^*J*_CP_ = 10 Hz, CH_a_ or CH_c_), 133.0 (d, ^3^*J*_CP_ = 8 Hz, CH_a_ or CH_c_), 129.6 (s, CH_d_), 129.3 (s, CH_b_), 127.8 (d, ^1^*J*_CP_ = 66 Hz, C_1_), 125.4 (s, CH_e_), 42.0 (s, CH_2_), 35.3 (s, C_(*t*Bu)_), 35.0 (s, C_(*t*Bu)_), 31.5 (s, CH_3(*t*Bu)_), 31.4 (s, CH_3(*t*Bu)_). ^31^P{^1^H} NMR (202 MHz, CD_2_Cl_2_, 25 °C) δ: 0.6.

### Synthesis of Gold(I)–1-Propene Complex **14**

In a glovebox, a Schlenk flask was charged with silver hexafluoroantimonate (8 mg, 0.022 mmol) in dichloromethane (1 mL). The corresponding gold(I) chloride complex (21 mg, 0.02 mmol) was transferred into a small glass vial and dissolved in dichloromethane (1 mL). The vial solution was loaded into a plastic syringe equipped with a stainless steel needle. Outside the glovebox, the solution of the gold(I) chloride complex was added to the AgSbF_6_ suspension while bubbling 1-propene and stirred for 10 min. The mixture was filtered through a short pad of Celite to remove the silver salts, and the solvent was removed under vacuum affording the complex **14** as colorless solid (23 mg, 87%). Crystals suitable for X-ray diffraction were grown by slow diffusion of pentane into a dichloromethane solution of complex **14**. NMR analysis showed the presence of two isomers at rt with a 1:1 ratio that were analyzed altogether as **14** and **14′**. ^1^H NMR (400 MHz, CD_2_Cl_2_, 25 °C) δ: 7.76 (d, 3H, ^3^*J*_HH_ = 8.1 Hz, CH_b_ + CH_b′_), 7.76 (d, 3H, ^3^*J*_HH_ = 8.1 Hz, CH_b′_), 7.54 (d, 3H, ^4^*J*_HH_ = 2.0 Hz, CH_a_), 7.51 (d, 3H, ^4^*J*_HH_ = 2.0 Hz, CH_a′_), 7.44 (dd, 3H, ^3^*J*_HH_ = 8.1 Hz, ^4^*J*_HH_ = 2.0 Hz, CH_c_), 7.44 (dd, 3H, ^3^*J*_HH_ = 8.1 Hz, ^4^*J*_HH_ = 2.0 Hz, CH_c’_),7.26 (d, 6H, ^3^*J*_HH_ = 8.6 Hz, CH_e_), 7.25 (d, 6H, ^3^*J*_HH_ = 8.6 Hz, CH_e′_), 6.76 (d, 6H, ^3^*J*_HH_ = 8.1 Hz, CH_d_), 6.76 (d, 6H, ^3^*J*_HH_ = 8.1 Hz, CH_d′_), 4.64 (m, 1H, CH_prop_), 4.50 (m, 1H, CH_(prop)’_), 3.45 (m, 2H, CH_2(prop)_), 3.45 (m, 2H, CH_2(prop)′_),1.27 (s, 27H, CH_3(*t*Bu)_), 1.23 (s, 3H, CH_3(prop)_), 1.23, (s, 3H, CH_3(prop)′_), 1.24 (s, 27H, CH_3(*t*Bu)_). ^13^C{^1^H} NMR (100 MHz, CD_2_Cl_2_, 25 °C) δ: 152.3 (s, C_5_), 152.3 (s, C_5′_), 143.5 (d, ^2^*J*_CP_ = 16 Hz, C_3_), 143.5 (d, ^2^*J*_CP_ = 16 Hz, C_3′_), 138.6 (d, ^3^*J*_CP_ = 7 Hz, C_4_), 138.5 (d, ^3^*J*_CP_ = 7 Hz, C_4’_), 134.1 (d, ^3^*J*_CP_ = 6 Hz, CH_c_), 134.0 (d, ^3^*J*_CP_ = 6 Hz, CH_c′_), 133.7 (s, CH_a_), 133.7 (s, CH_a′_), 132.3 (s, CH_(prop)_), 132.2 (s, CH_(prop)′_), 130.0 (s, CH_b_), 130.0 (s, CH_b_), 129.9 (s, CH_d_), 129.9 (s, CH_d′_), 127.7 (d, ^2^*J*_CP_ = 62 Hz, C_1_), 127.7 (d, ^1^*J*_CP_ = 62 Hz, C_1′_), 126.1 (s, CH_e_ + CH_e′_), 101.6 (d, ^2^*J*_CP_ = 5 Hz, CH_2(prop)_), 101.1 (d, ^2^*J*_CP_ = 5 Hz, CH_2(prop)′_), 35.4 (s, C_(*t*Bu)_), 35.2 (s, C_(*t*Bu)_), 22.9 (s, CH_3(prop)_), 21.9 (s, CH_3(prop)′_), 35.0 (s, C_(*t*Bu)_), 31.6 (s, CH_3(*t*Bu)_), 31.3 (s, CH_3(*t*Bu)_). ^31^P{^1^H} NMR (202 MHz, CD_2_Cl_2_, 25 °C) δ: 13.8 and 13.6.

### General Procedure for the Gold(I)-Catalyzed Hydroamination of Ethylene

A mixture of amide (0.20 mmol), gold chloride complex (0.01 mmol), and silver hexafluoroantimoniate (4 mg, 0.01 mmol) in dioxane (1 mL) was placed in a Fischer Porter tube together with a magnetic stirring bar under a nitrogen atmosphere. The tube was freeze-pumped to remove the nitrogen gas, filled with the indicated ethylene pressure, and stirred at 100 °C for 18 h. After this time, the mixture was cooled down to rt and diluted in CH_2_Cl_2_ (5 mL), and anisole (22 mL, 0.20 mmol) was added as the internal standard. The mixture was then filtered through a short pad of Celite, the solvents were removed under reduced pressure, and the sample was analyzed by NMR spectroscopy in CDCl_3_.

## Supporting Information Available

NMR spectra, catalytic and kinetic studies, X-ray structural data, and computational details (PDF)
Cartesian coordinates (XYZ)
Crystallographic information (CIF) (CIF) (CIF) (CIF) (CIF) (CIF) (CIF) (CIF)

## Supplementary Material

cs1c05823_si_001.pdf

cs1c05823_si_002.cif

cs1c05823_si_003.cif

cs1c05823_si_004.cif

cs1c05823_si_005.cif

cs1c05823_si_006.cif

cs1c05823_si_007.cif

cs1c05823_si_008.cif

cs1c05823_si_009.xyz

cs1c05823_si_010.cif

## Accession Codes

CCDC 2129167–2129172 contain the supplementary crystallographic data for this paper. These data can be obtained free of charge via www.ccdc.cam.ac.uk/data_request/cif, or by emailing data_request@ccdc.cam.ac.uk, or by contacting The Cambridge Crystallographic Data Centre, 12 Union Road, Cambridge CB2 1EZ, UK; fax: +44 1223 336033.
