# Supplementary material for: Dicoordinate Au(I)–Ethylene Complexes as Hydroamination Catalysts
Source: ACS Catal. 2022 Mar 23;12(7):4227–41. doi: 10.1021/acscatal.1c05823 (PMC8981211; doi:10.1021/acscatal.1c05823)
Supplement: Supplementary file 1 — cs1c05823_si_001.pdf [file cs1c05823_si_001.pdf]

Dicoordinate Au(I)–Ethylene Complexes as Hydroamination Catalysts

Miquel Navarro*, Macarena G. Alférez, Morgane de Sousa, Juan Miranda-Pizarro, Jesús Campos*

† Instituto de Investigaciones Químicas (IIQ), Departamento de Química Inorgánica and Centro de Innovación en Química Avanzada (ORFEO−CINQA). Consejo Superior de Investigaciones Científicas (CSIC) and University of Sevilla, 41092 Sevilla, Spain

E-mail: miquel.navarro@iiq.csic.es; jesus.campos@iiq.csic.es

1. NMR spectroscopic experiments S2

2. Formation of Au(I)-Ag(I) multimetallic species S33

3. Formation of Au(I)-amine adducts S36

4. Catalytic experiments S37

5. Kinetic experiments S40

6. Crystal structure determinations S52

7. Buried volume analysis S58

8. Computational details S60

9. References S65

1. NMR spectroscopic experiments

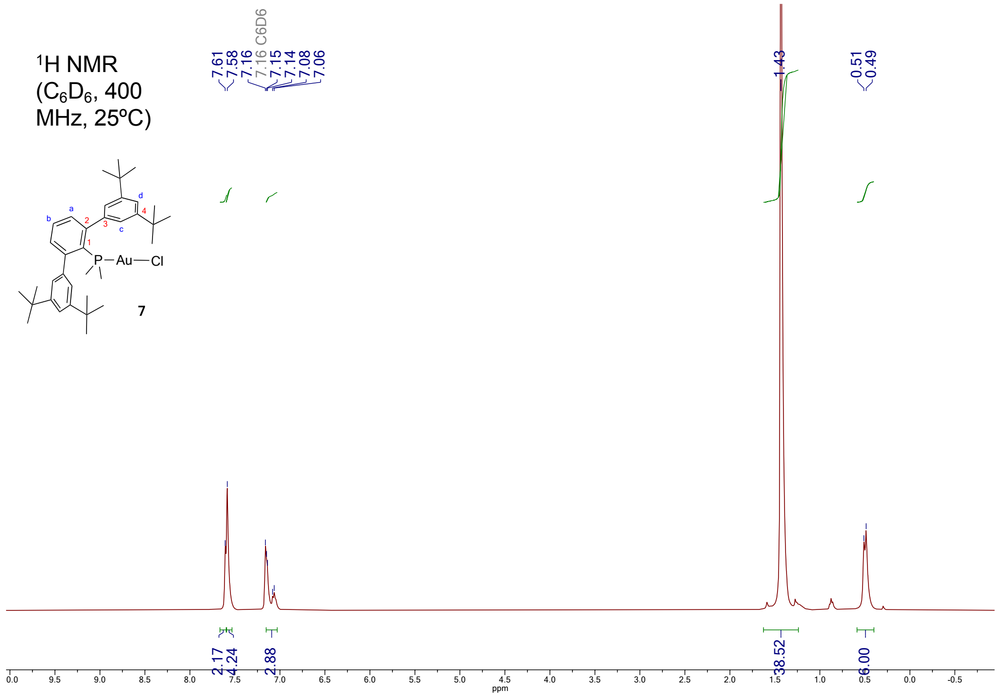

Figure S1. ^{1}H NMR of complex **7**.

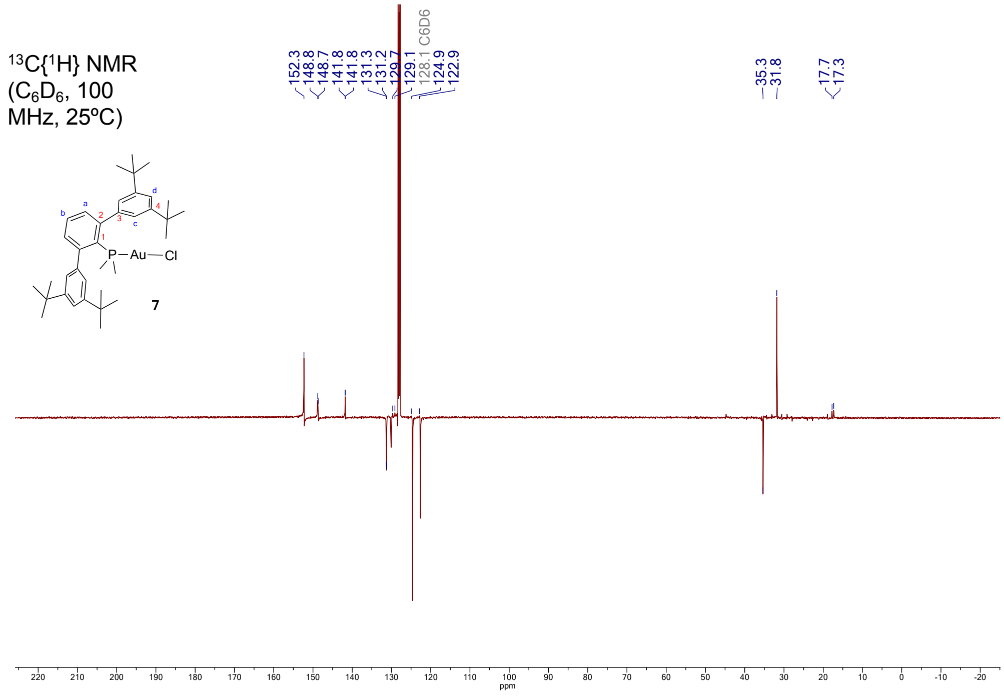

Figure S2. DEPT ^{13}C{^{1}H} NMR of complex **7**.

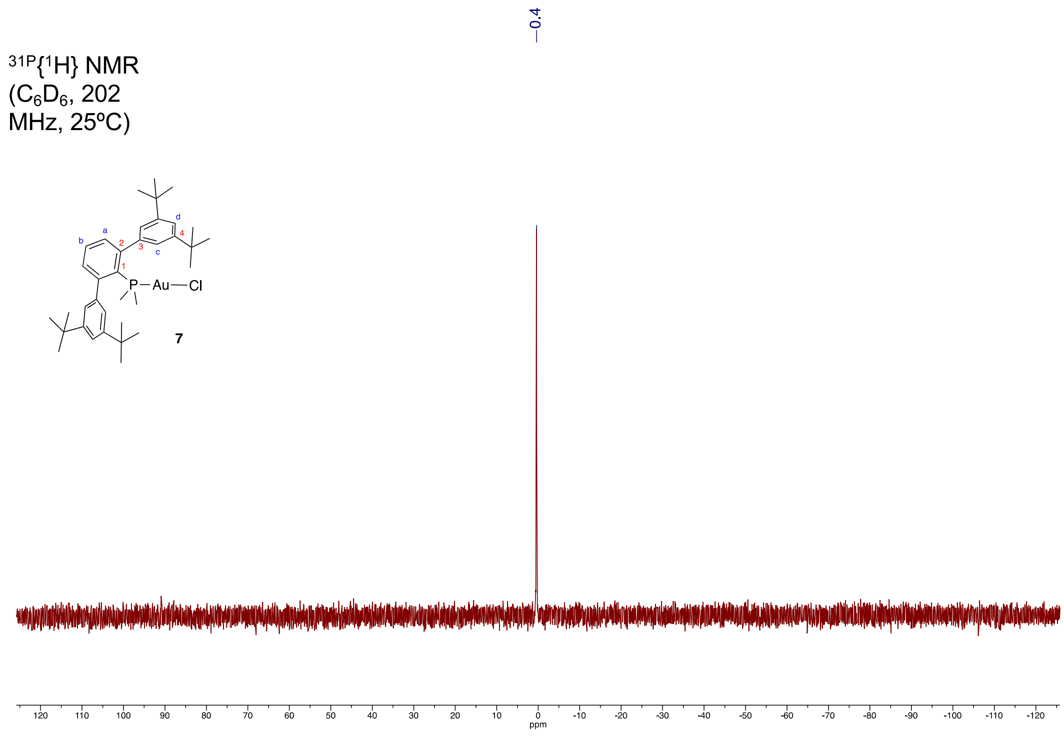

Figure S3. $^{31}P\{^{1}H\}$ NMR of complex **7**.

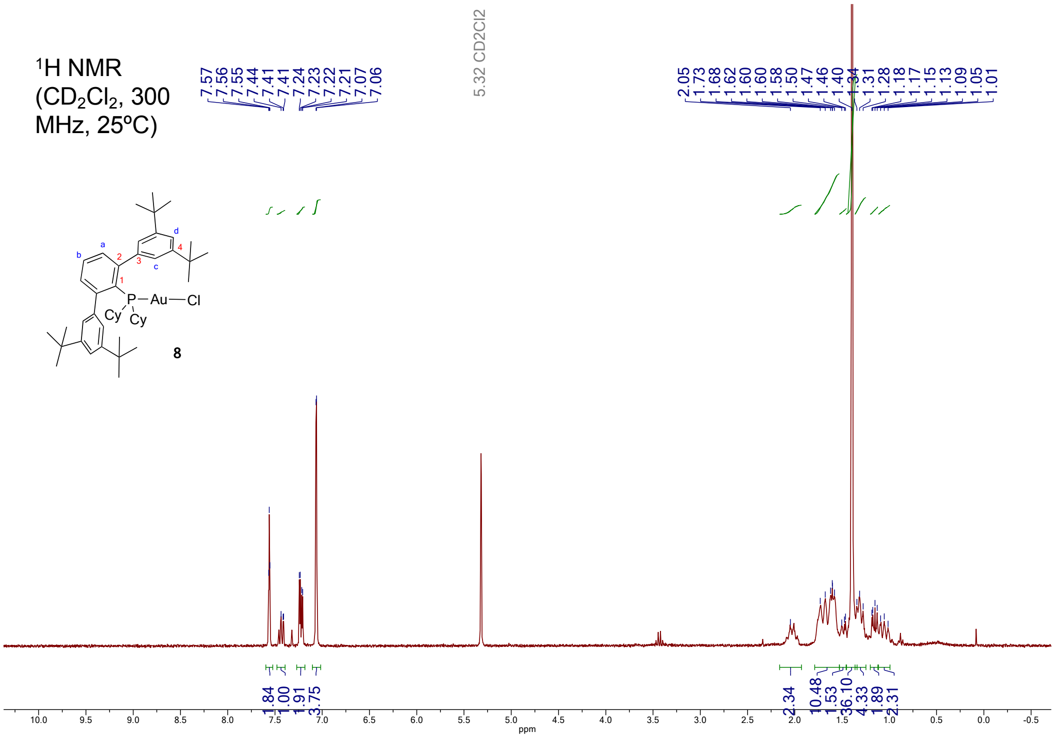

Figure S4. ^{1}H NMR of complex **8**.

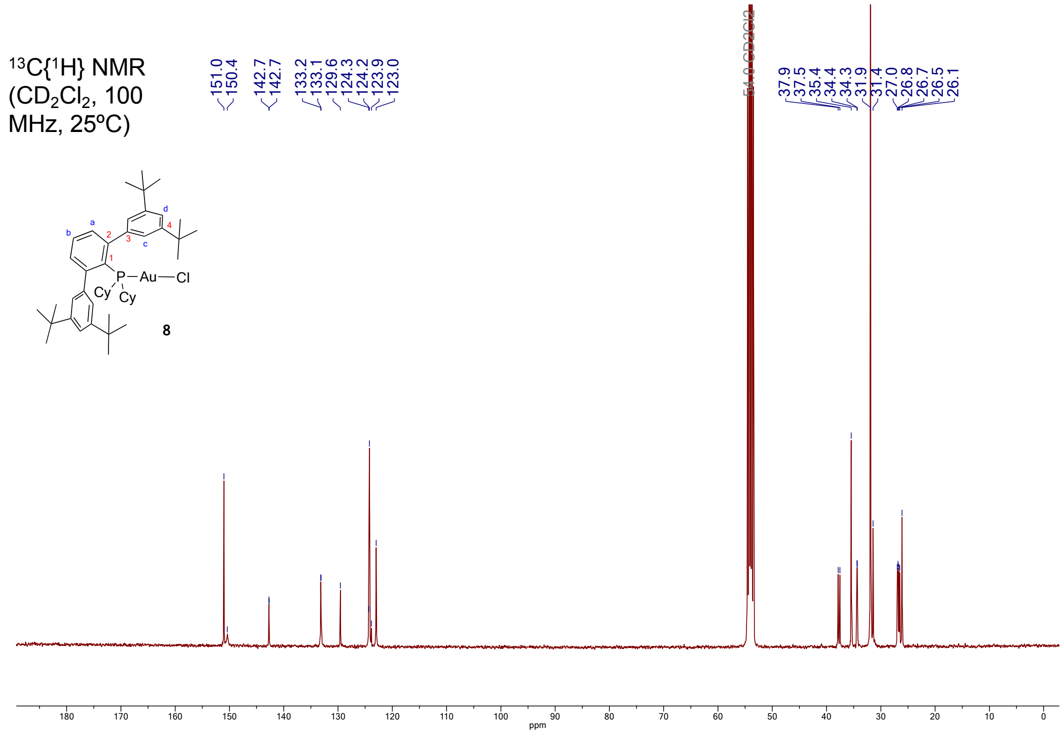

Figure S5. $^{13}C\{^1H\}$ NMR of complex **8**.

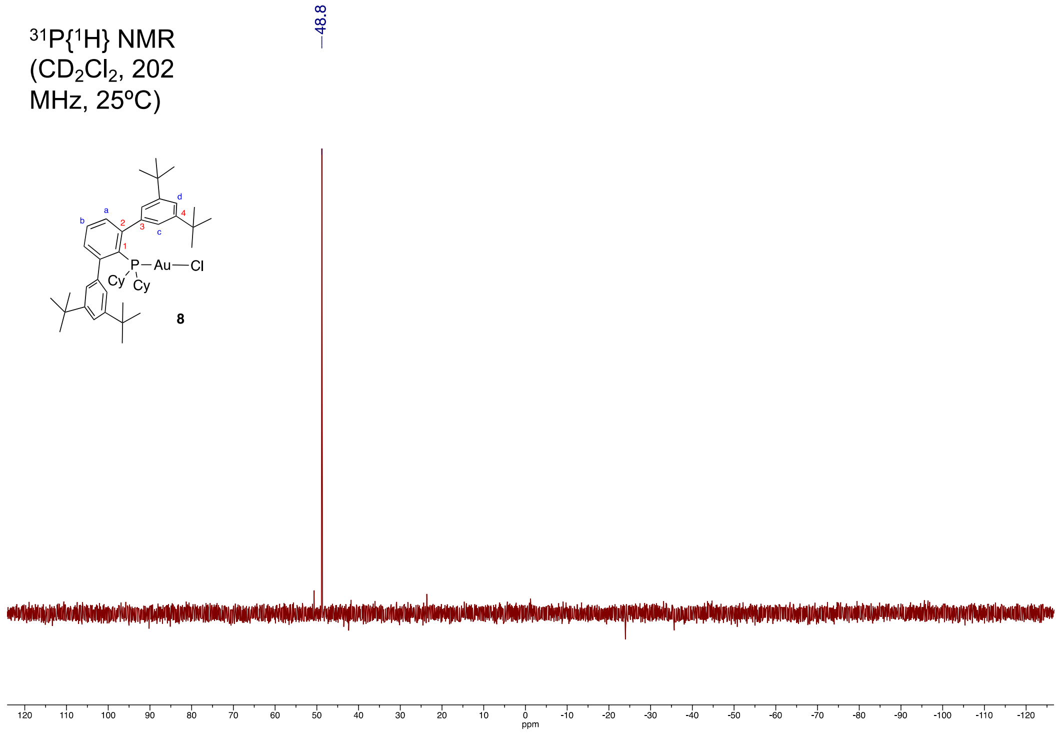

Figure S6. $^{31}P\{^1H\}$ NMR of complex **8**.

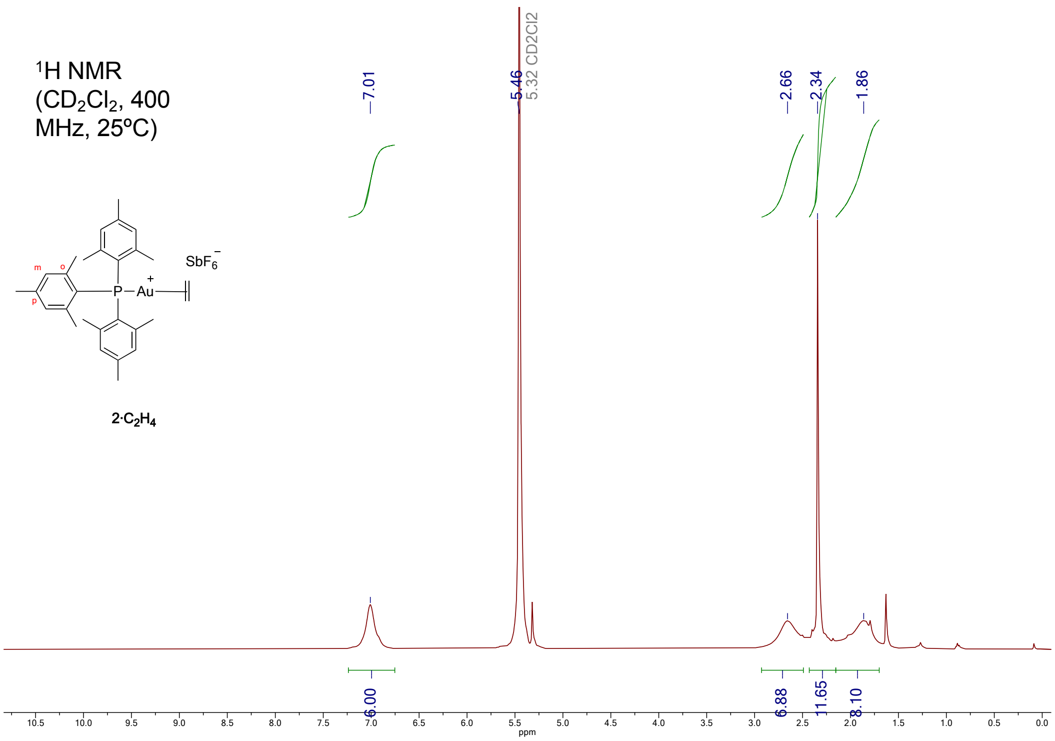

Figure S7. ^{1}H NMR of complex **2·C_2H_4** in excess of ethylene.

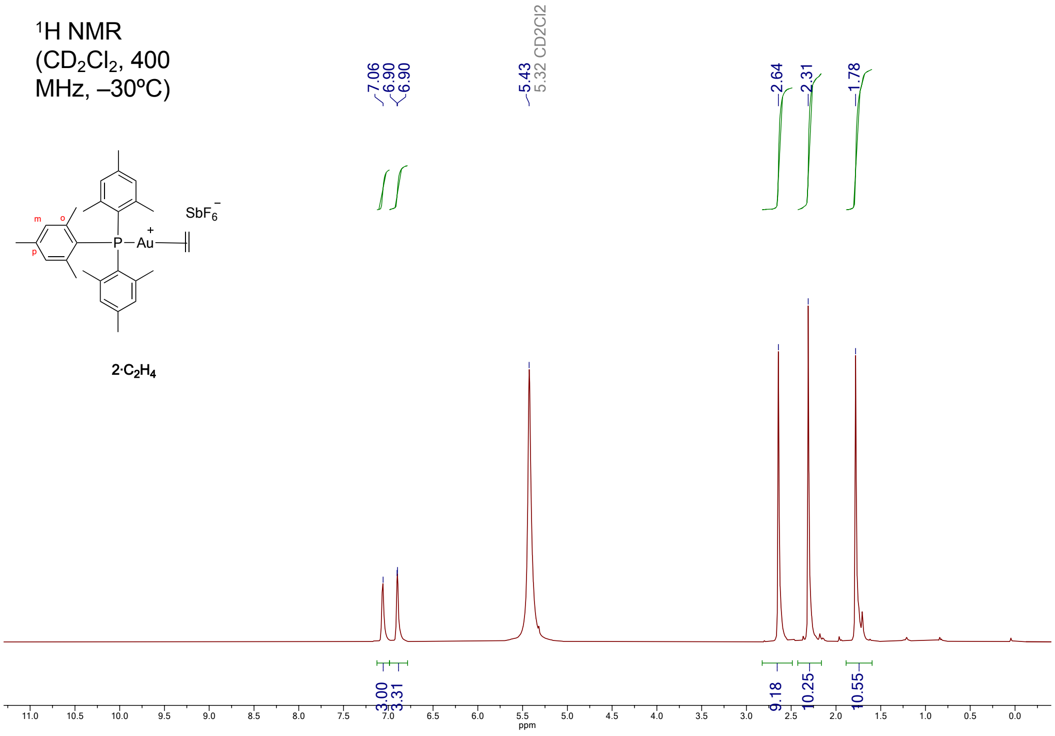

Figure S8. ^{1}H NMR of complex **2·C_2H_4** in excess of ethylene at –30 ºC.

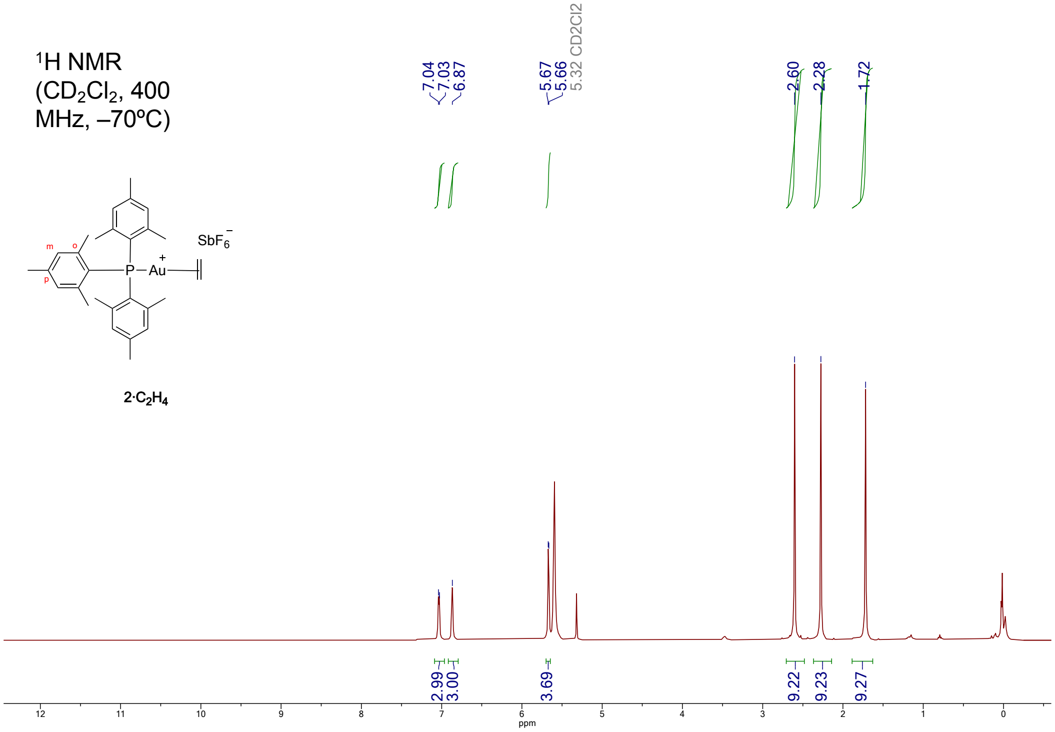

Figure S9. ^{1}H NMR of complex **2·C_2H_4** in excess of ethylene at –70 ºC.

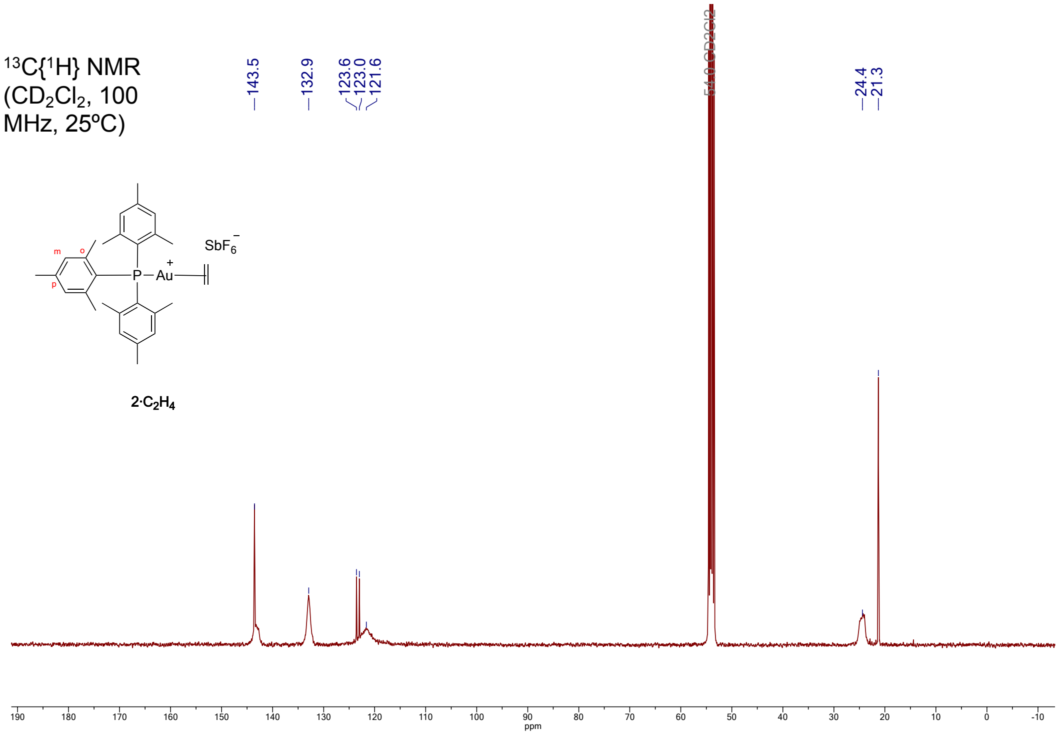

Figure S10. ^{13}C{^{1}H} NMR of complex **2·C_2H_4** in excess of ethylene at 25 ºC.

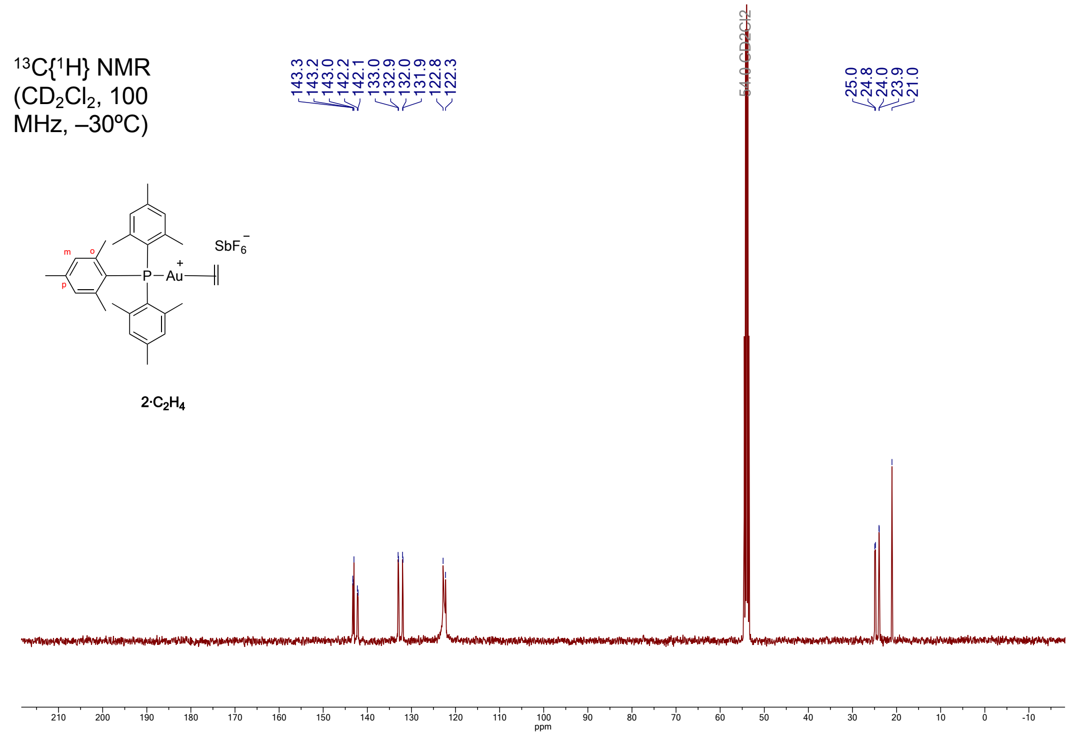

Figure S11. $^{13}C\{^1H\}$ NMR of complex **2·C$_2$H$_4$** in excess of ethylene at –30 ºC.

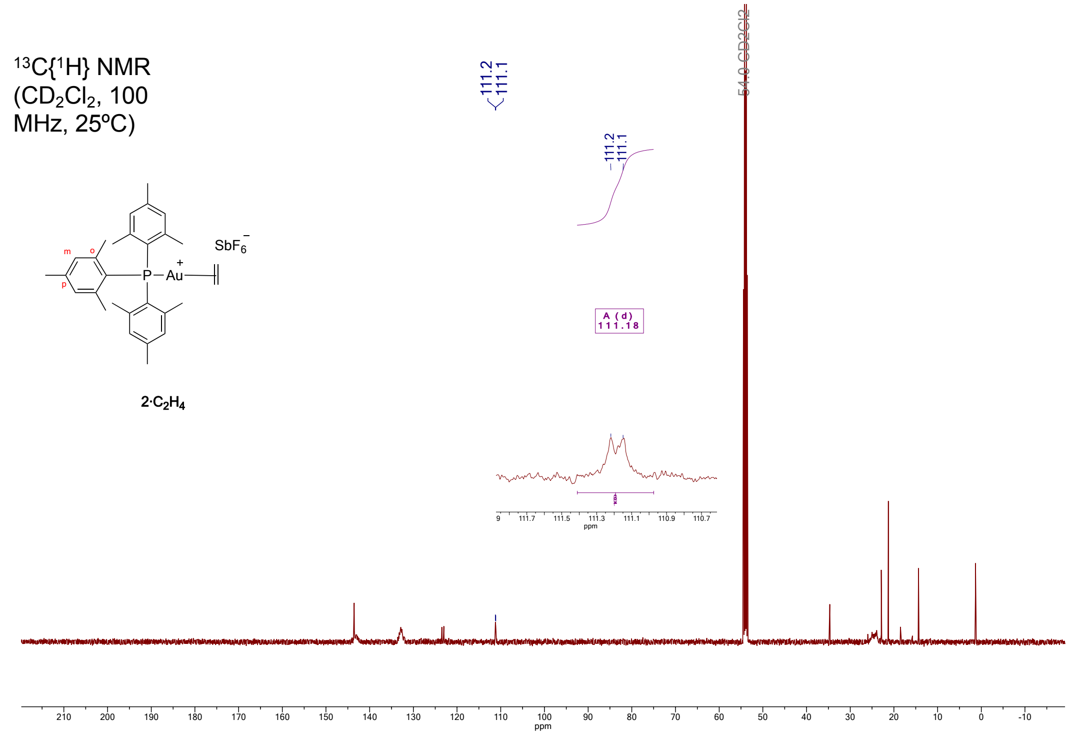

Figure S12. $^{13}C\{^1H\}$ NMR of complex **2·C$_2$H$_4$** with inset of the coordinated ethylene region at 25 ºC.

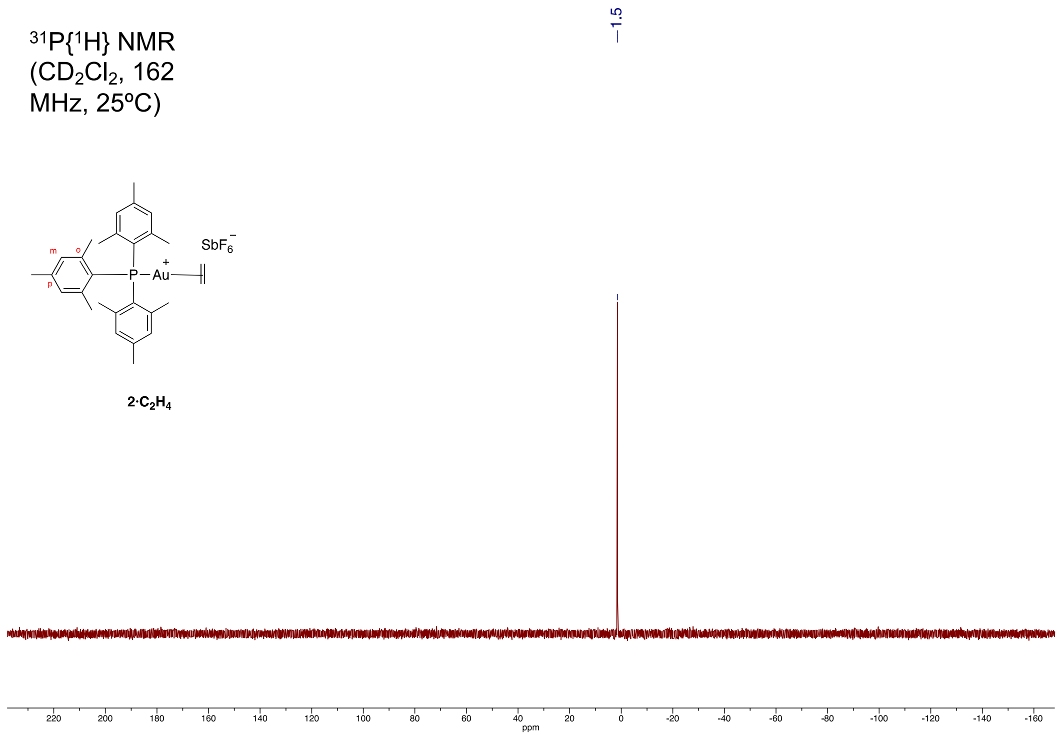

Figure S13. $^{31}P\{^1H\}$ NMR of complex $\mathbf{2{\cdot}C_2H_4}$.

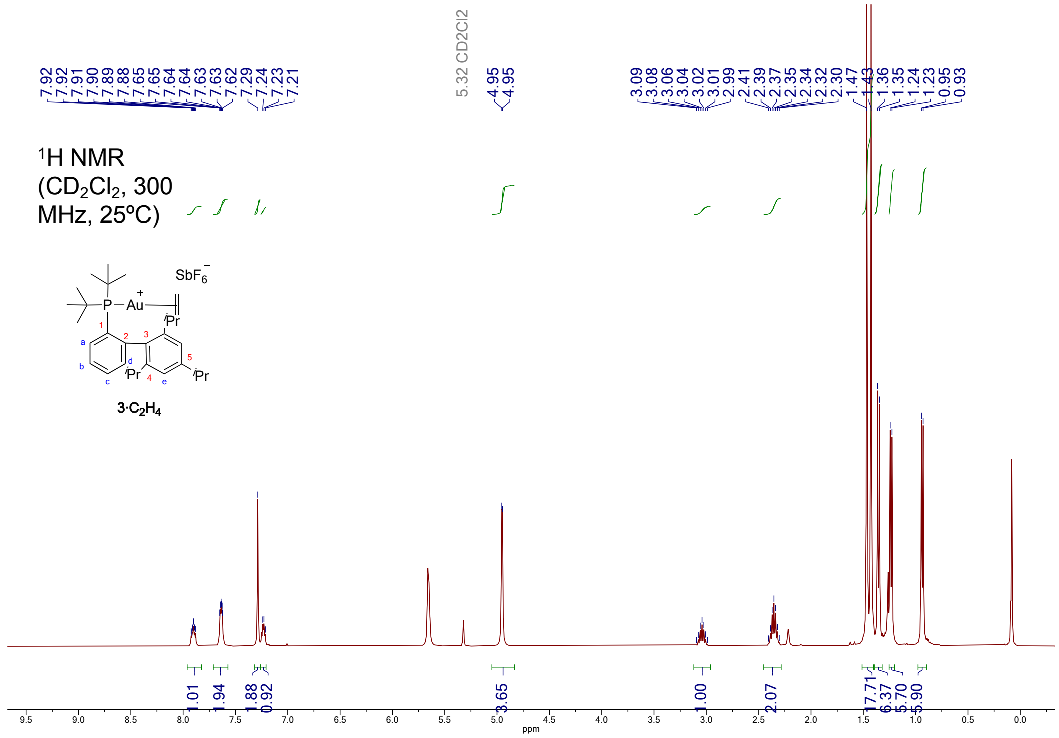

Figure S14. ^1H NMR of complex $\mathbf{3{\cdot}C_2H_4}$.

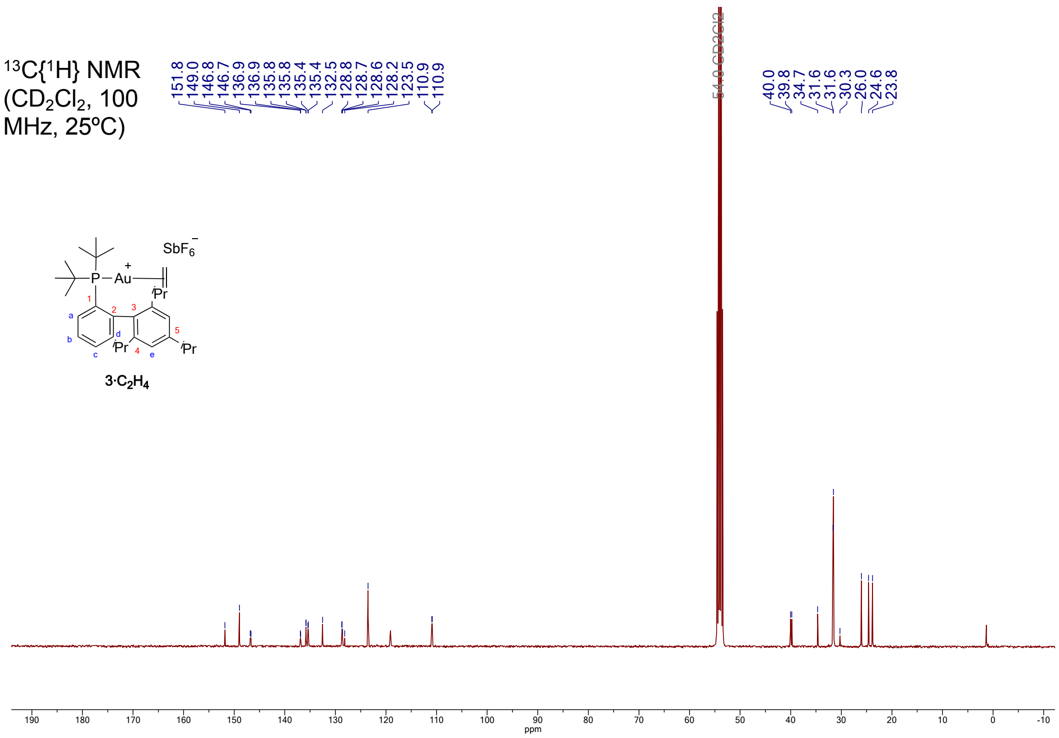

Figure S15. $^{13}C\{^1H\}$ NMR of complex $\mathbf{3{\cdot}C_2H_4}$.

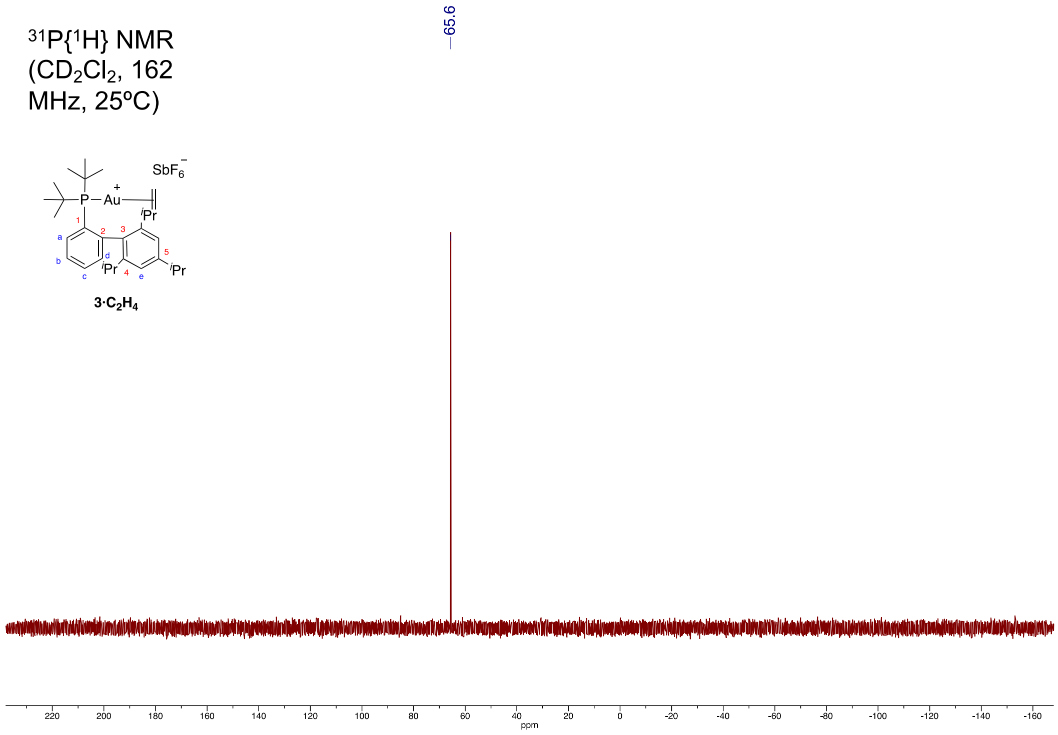

Figure S16. $^{31}P\{^1H\}$ NMR of complex $\mathbf{3{\cdot}C_2H_4}$.

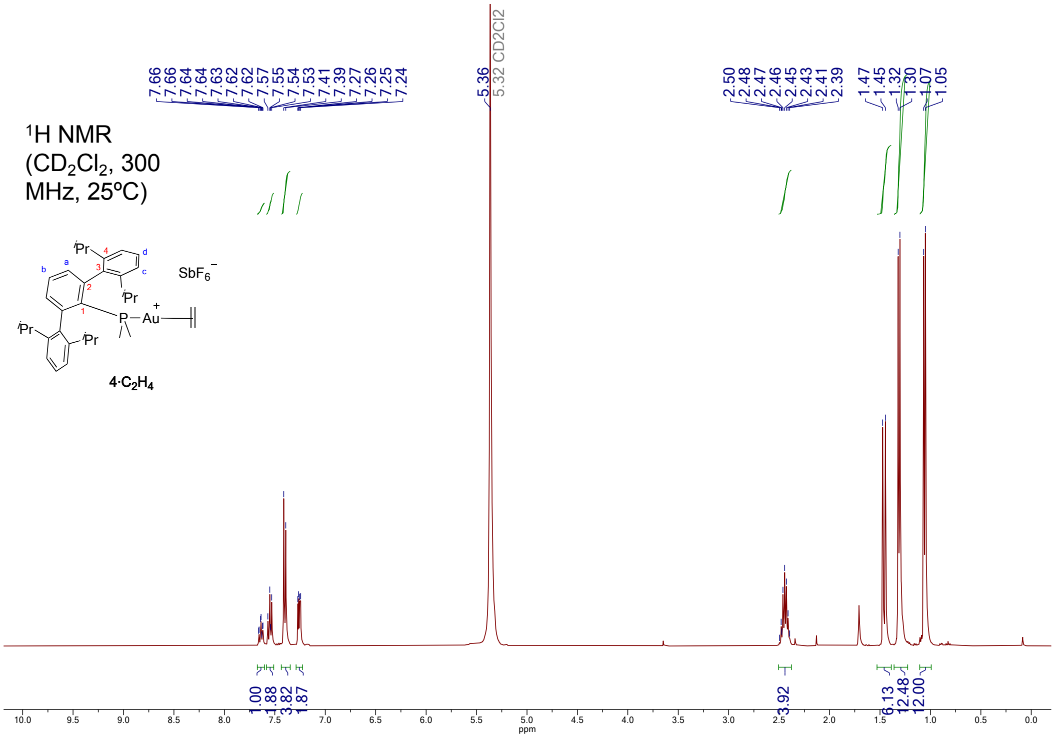

Figure S17. ^{1}H NMR of complex **4·C$_2$H$_4$** in excess of ethylene at 25 ºC.

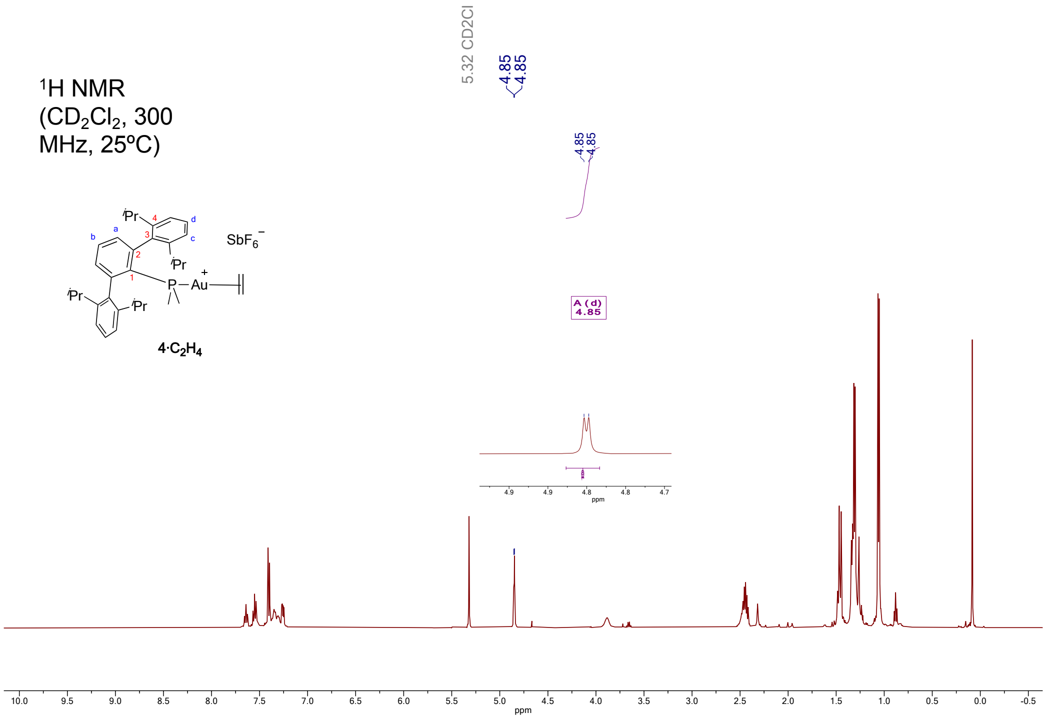

Figure S18. ^{1}H NMR of complex **4·C$_2$H$_4$** with inset of the coordinated ethylene region at 25 ºC.

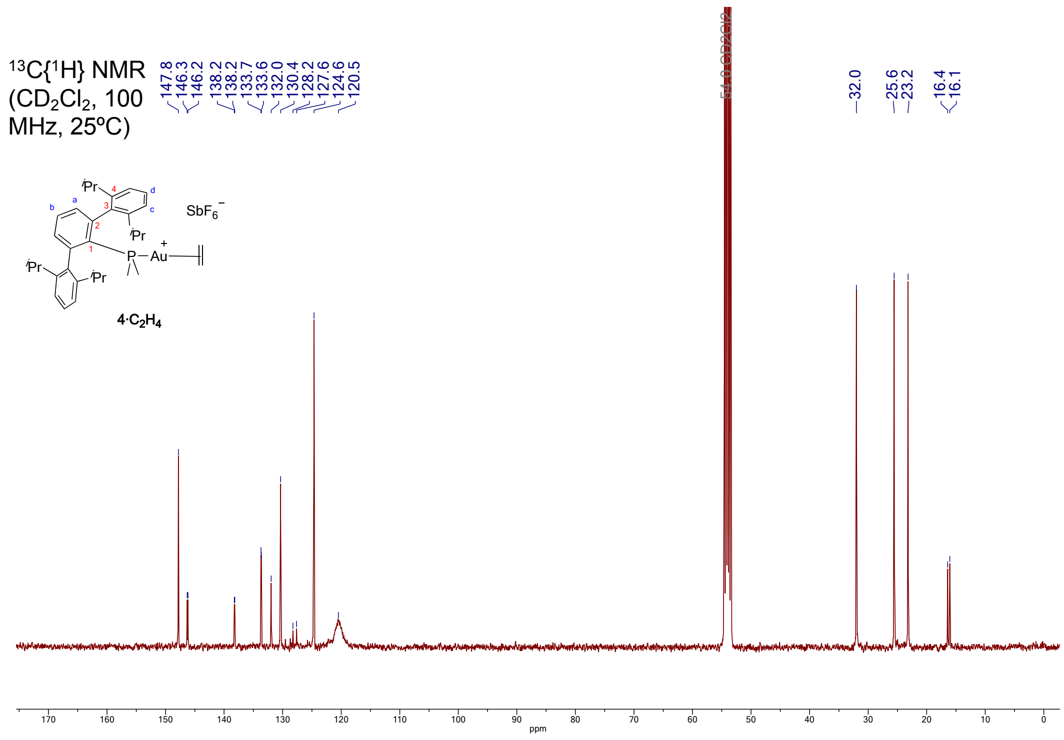

Figure S19. $^{13}C\{^1H\}$ NMR of complex **4·C₂H₄** in excess of ethylene at 25 ºC.

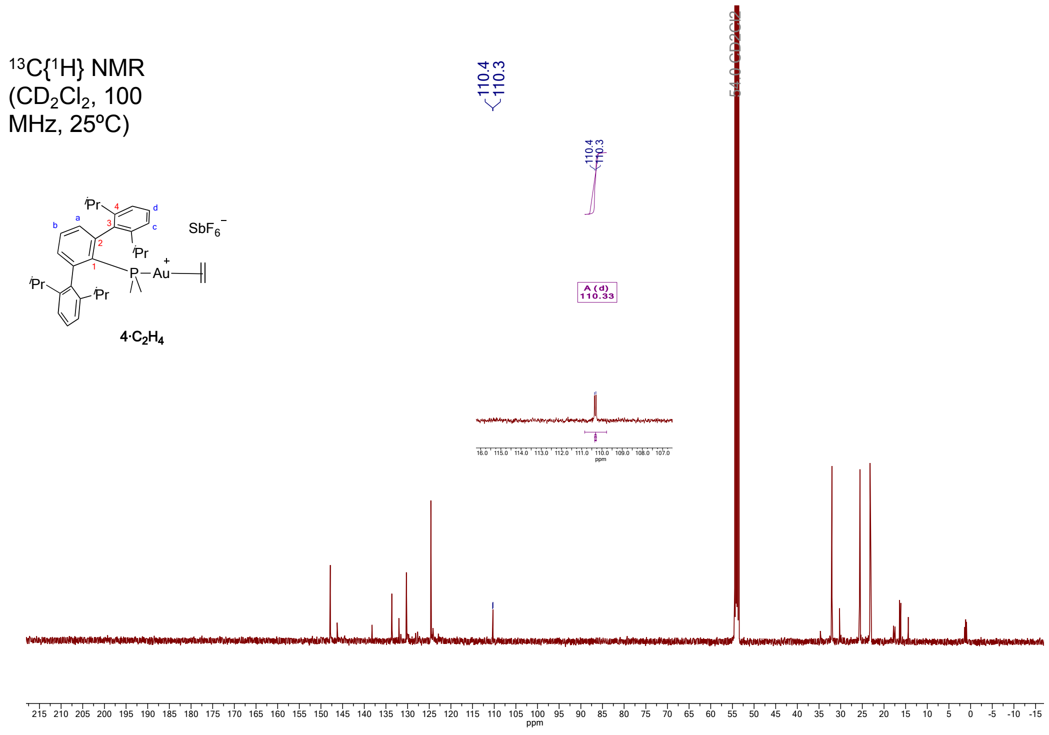

Figure S20. $^{13}C\{^1H\}$ NMR of complex **4·C_2H_4** with inset of the coordinated ethylene region at 25 ºC.

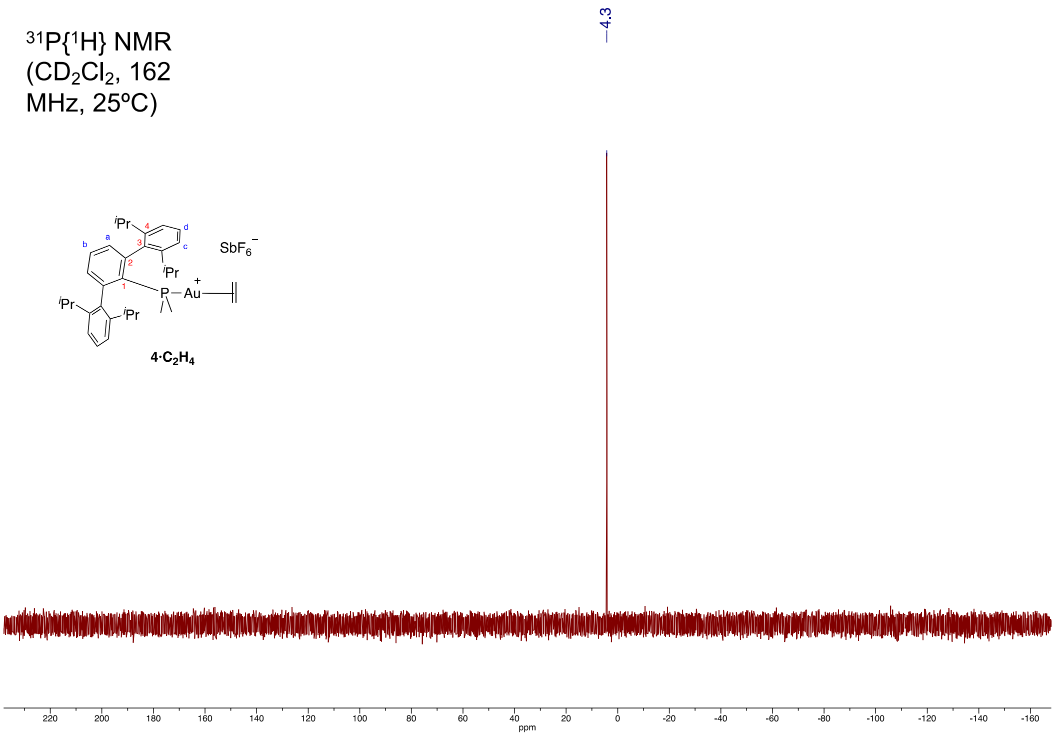

Figure S21. $^{31}P\{^1H\}$ NMR of complex **4·C$_2$H$_4$**.

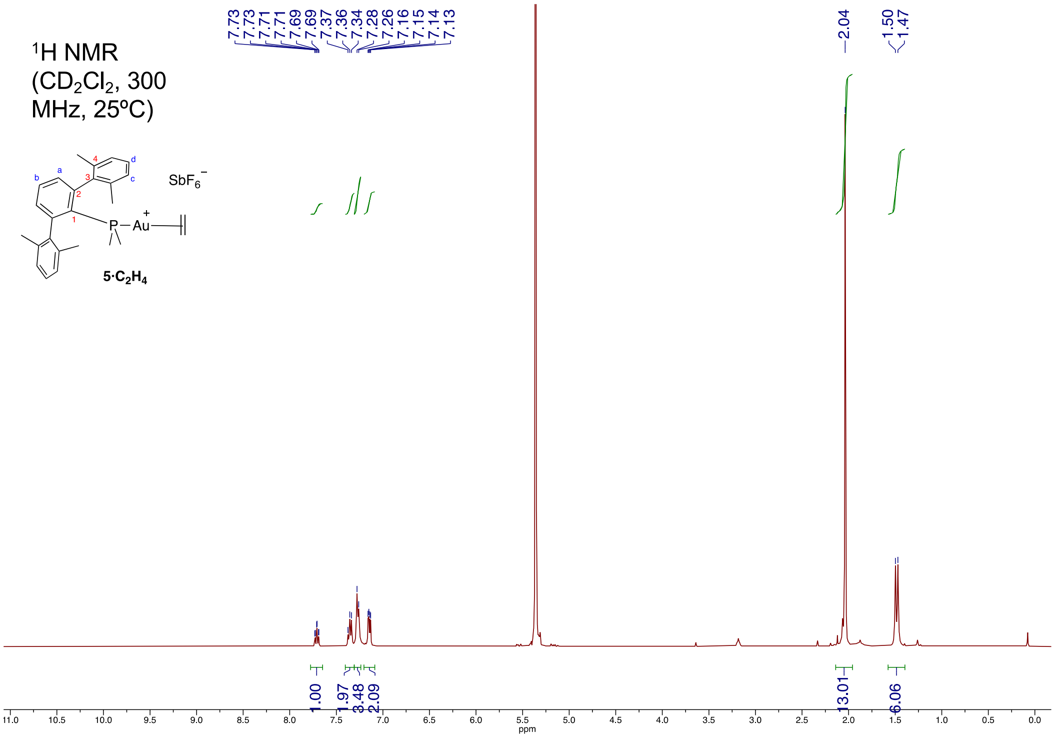

Figure S22. ^1H NMR of complex **5·C$_2$H$_4$** in excess of ethylene at 25 ºC.

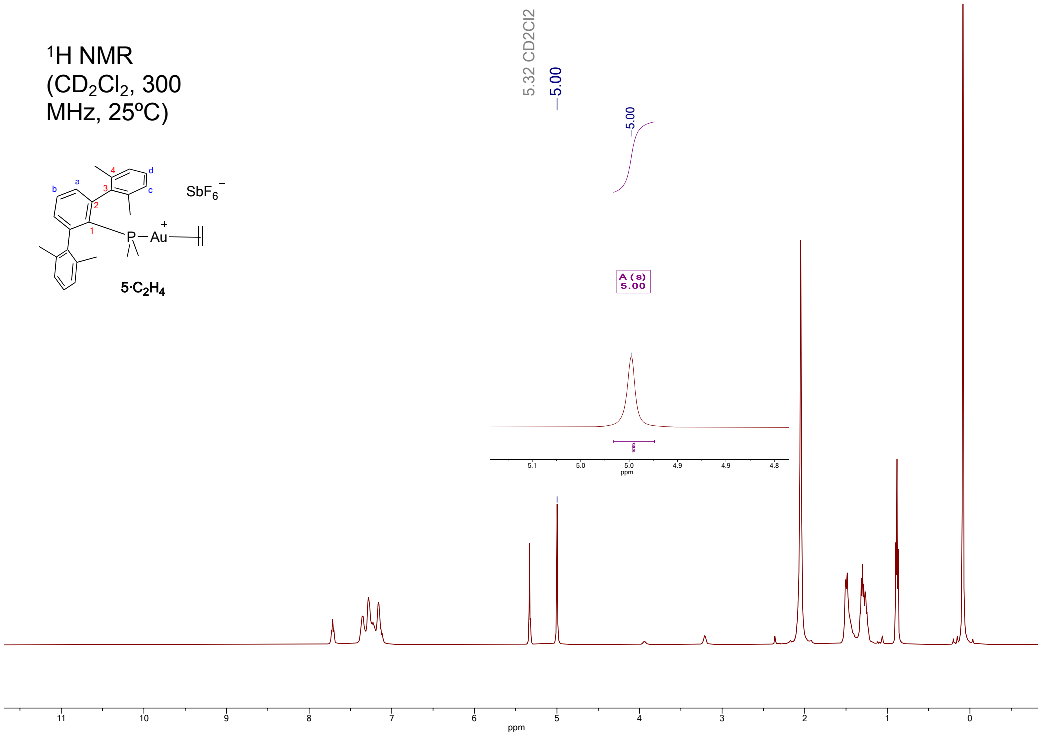

Figure S23. ^{1}H NMR of complex **5·C$_2$H$_4$** with inset of the coordinated ethylene region at 25 ºC.

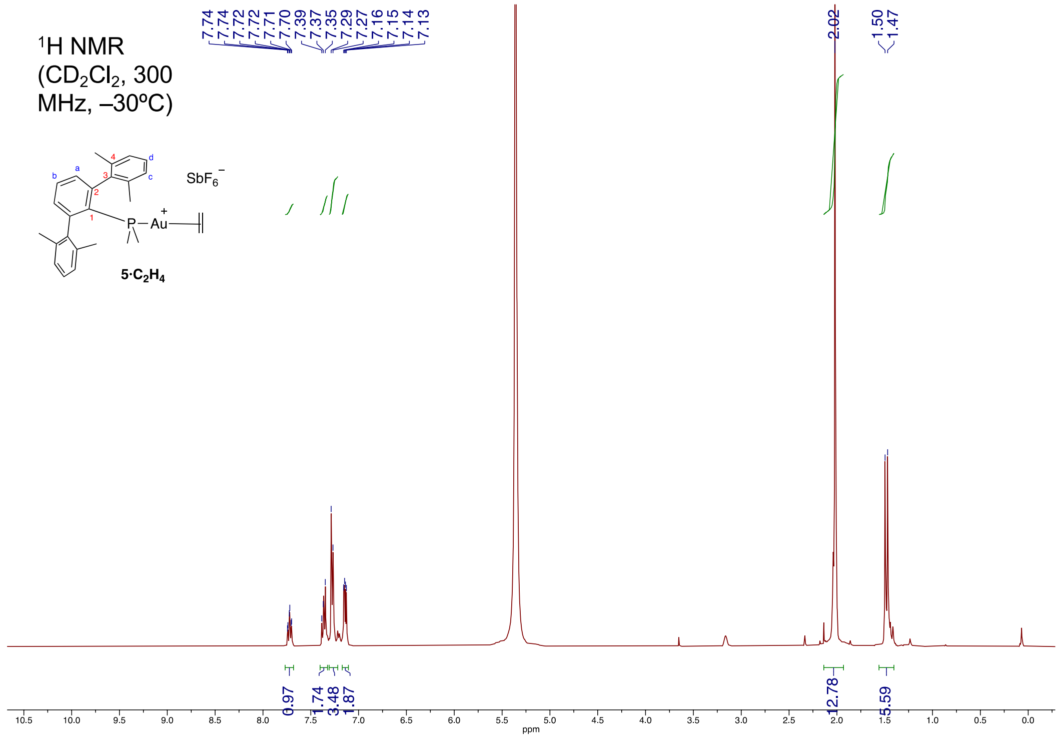

Figure S24. ^{1}H NMR of complex **5·C$_2$H$_4$** in excess of ethylene at –30 ºC.

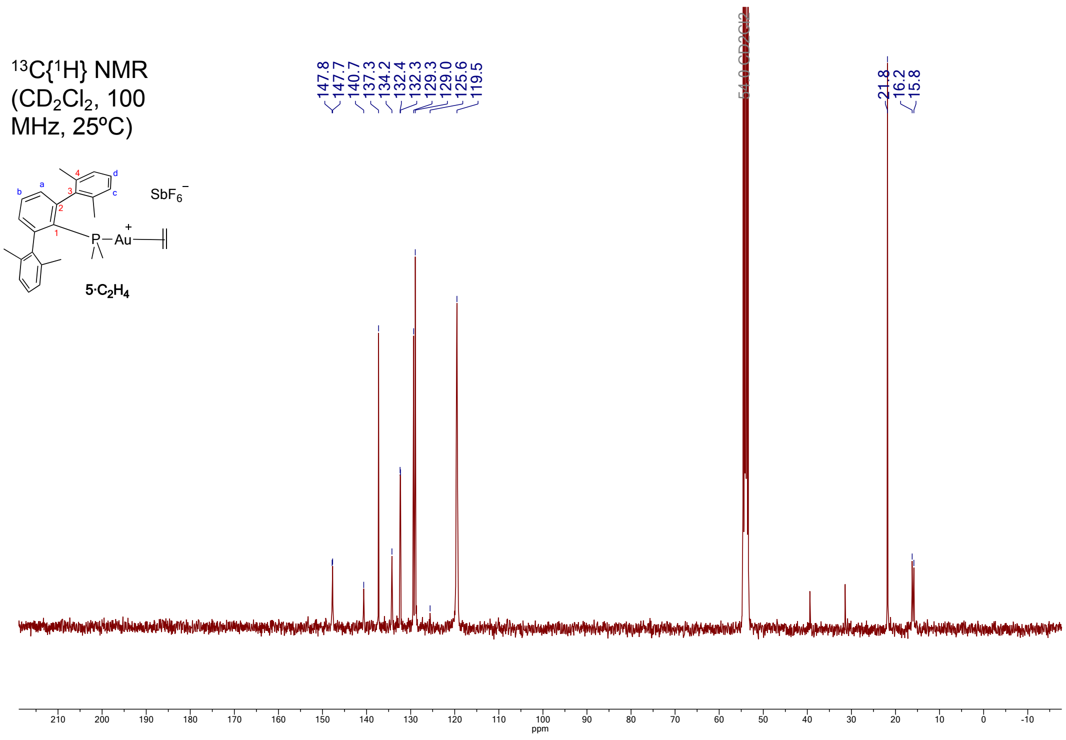

Figure S25. $^{13}C\{^1H\}$ NMR of complex $\mathbf{5{\cdot}C_2H_4}$ in excess of ethylene at 25 ºC.

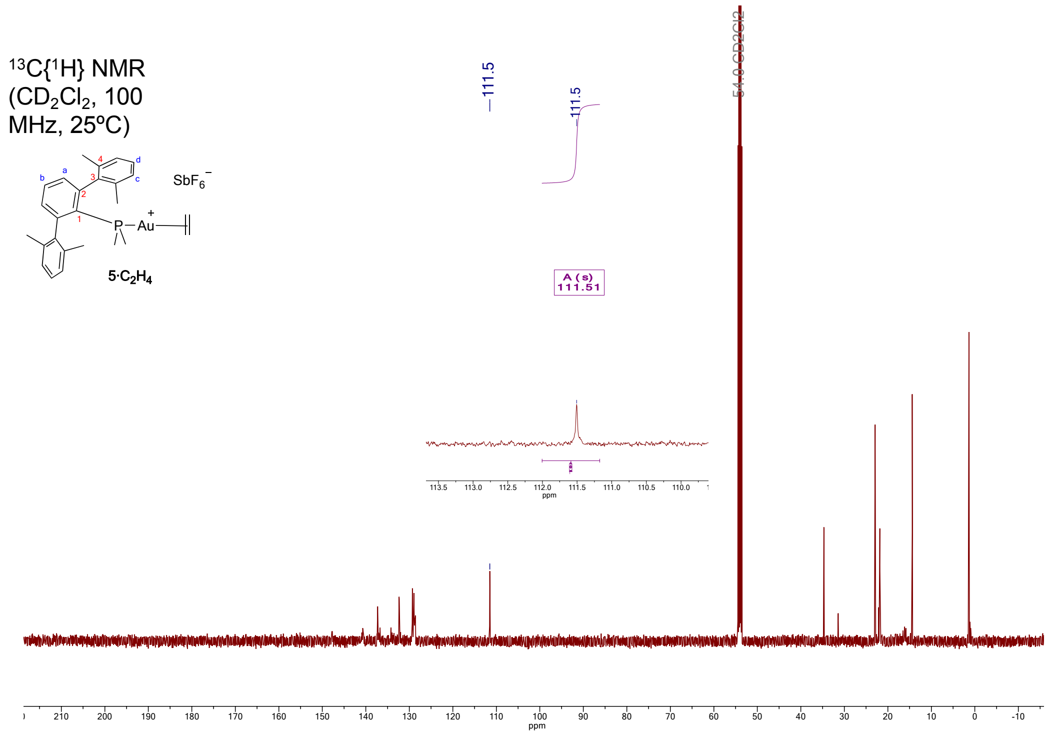

Figure S26. $^{13}C\{^1H\}$ NMR of complex $\mathbf{5{\cdot}C_2H_4}$ with inset of the coordinated ethylene region at 25 ºC.

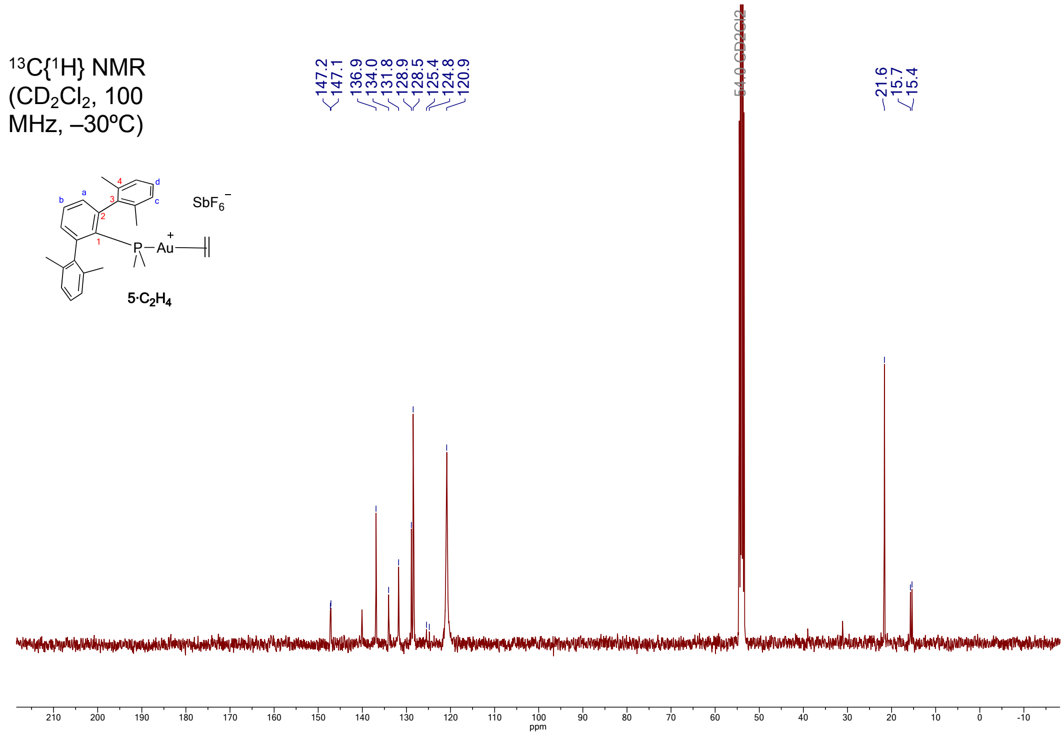

Figure S27. $^{13}C\{^{1}H\}$ NMR of complex **5·C$_2$H$_4$** in excess of ethylene at –30 ºC.

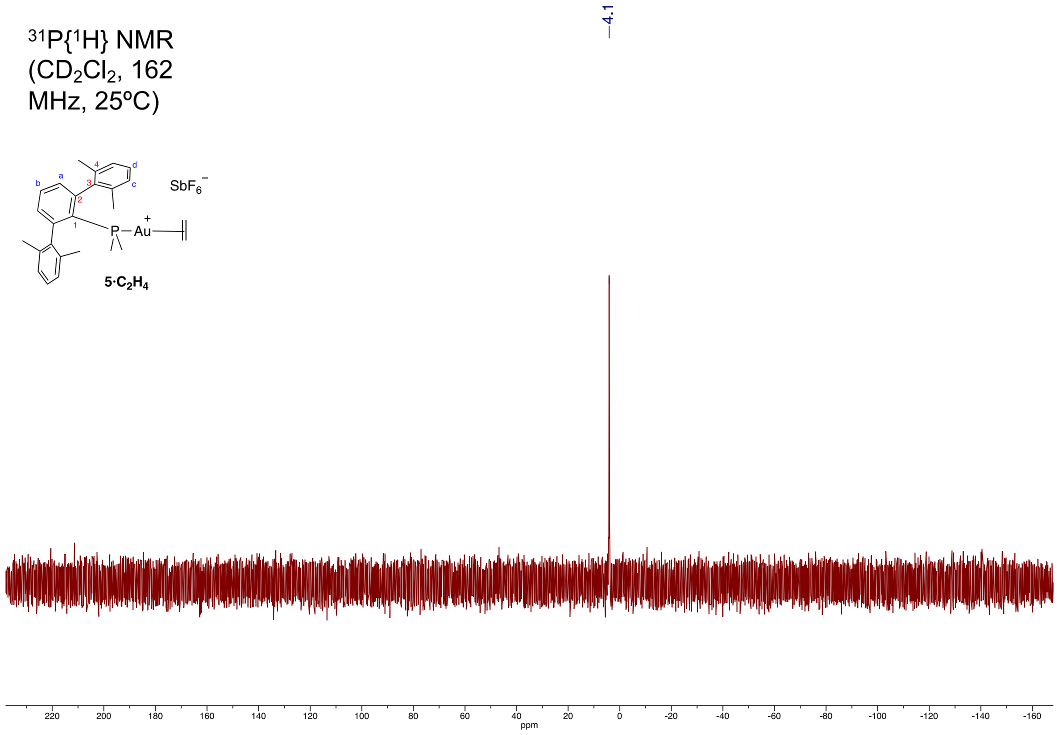

Figure S28. $^{31}P\{^{1}H\}$ NMR of complex **5·C$_2$H$_4$**.

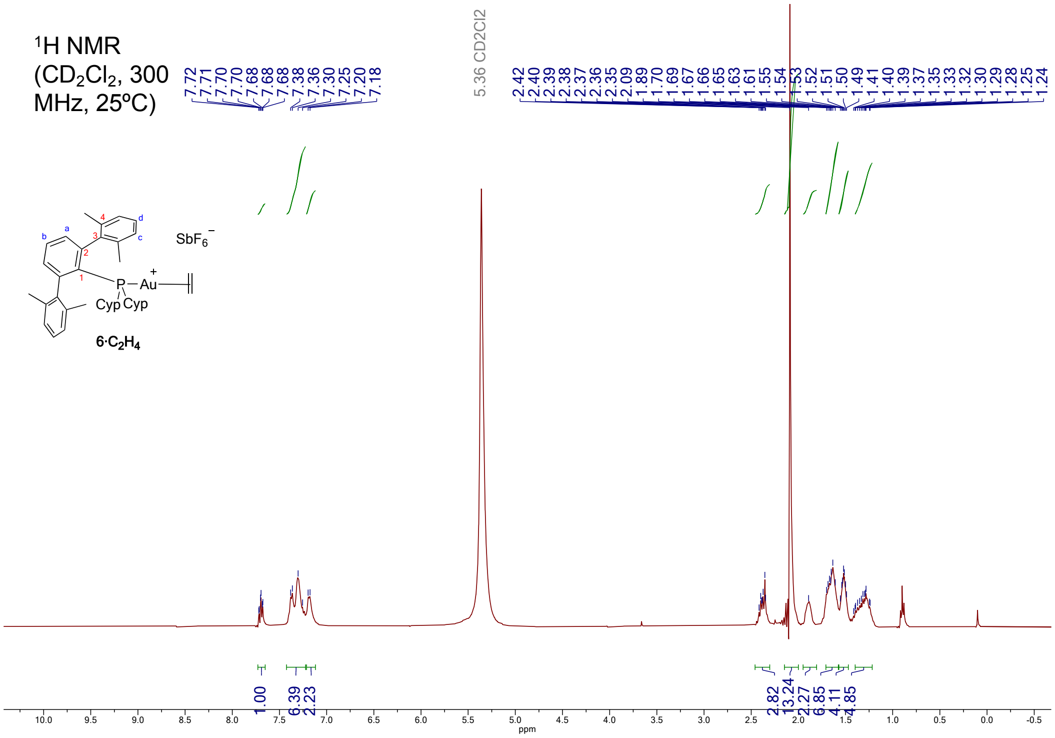

Figure S29. ^{1}H NMR of complex **6·C$_2$H$_4$** in excess of ethylene at 25 ºC.

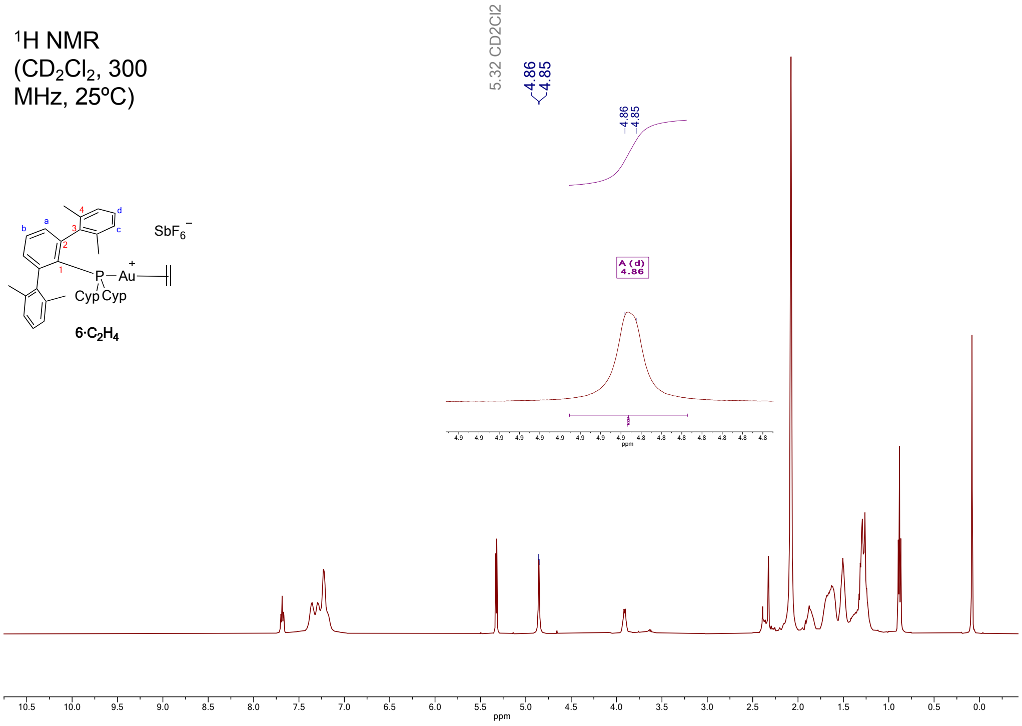

Figure S30. ^{1}H NMR of complex **6·C$_2$H$_4$** with inset of the coordinated ethylene region at 25 ºC.

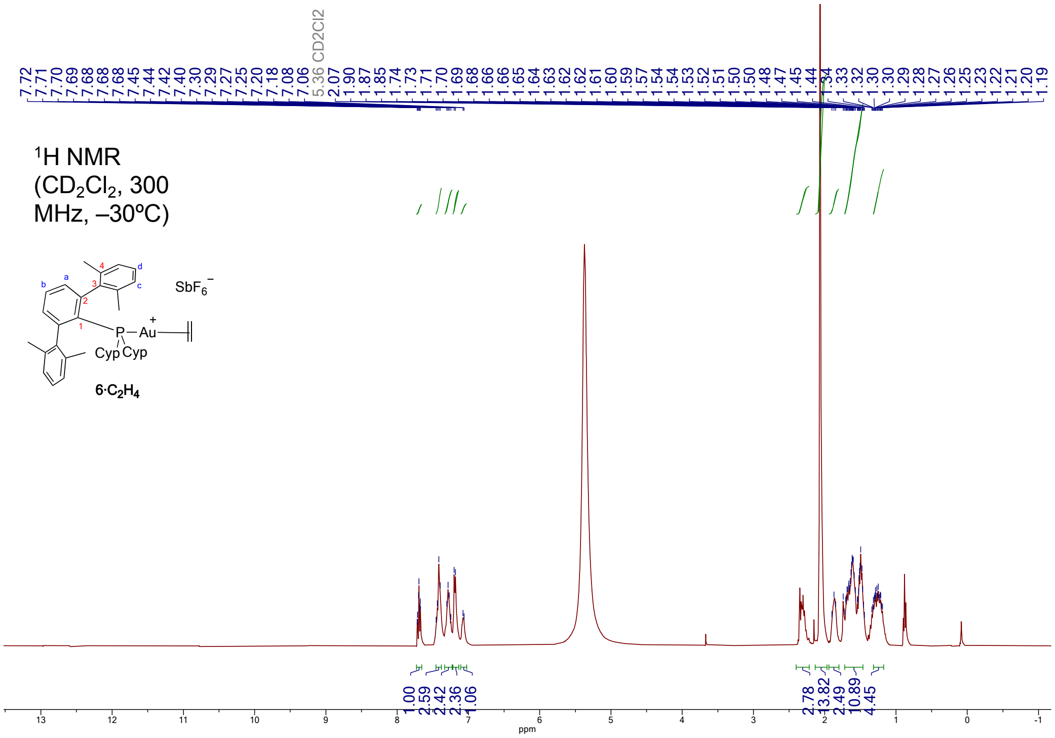

Figure S31. ^{1}H NMR of complex **6·C$_2$H$_4$** in excess of ethylene at –30 ºC.

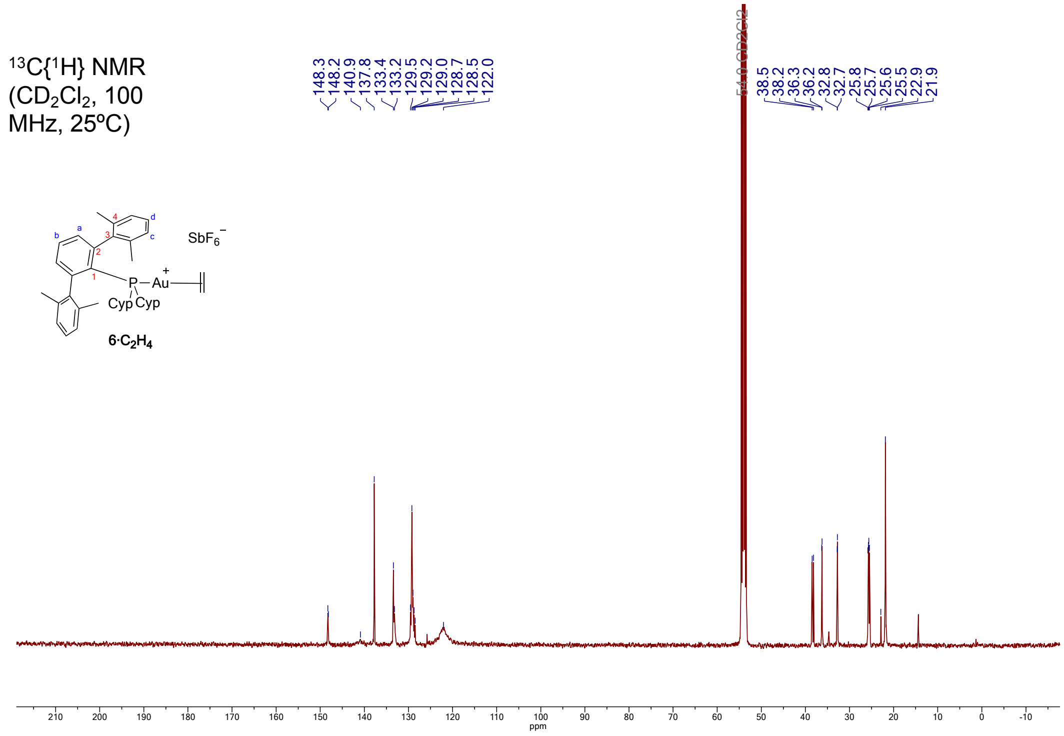

Figure S32. $^{13}C\{^{1}H\}$ NMR of complex **6·C$_2$H$_4$** in excess of ethylene at 25 ºC.

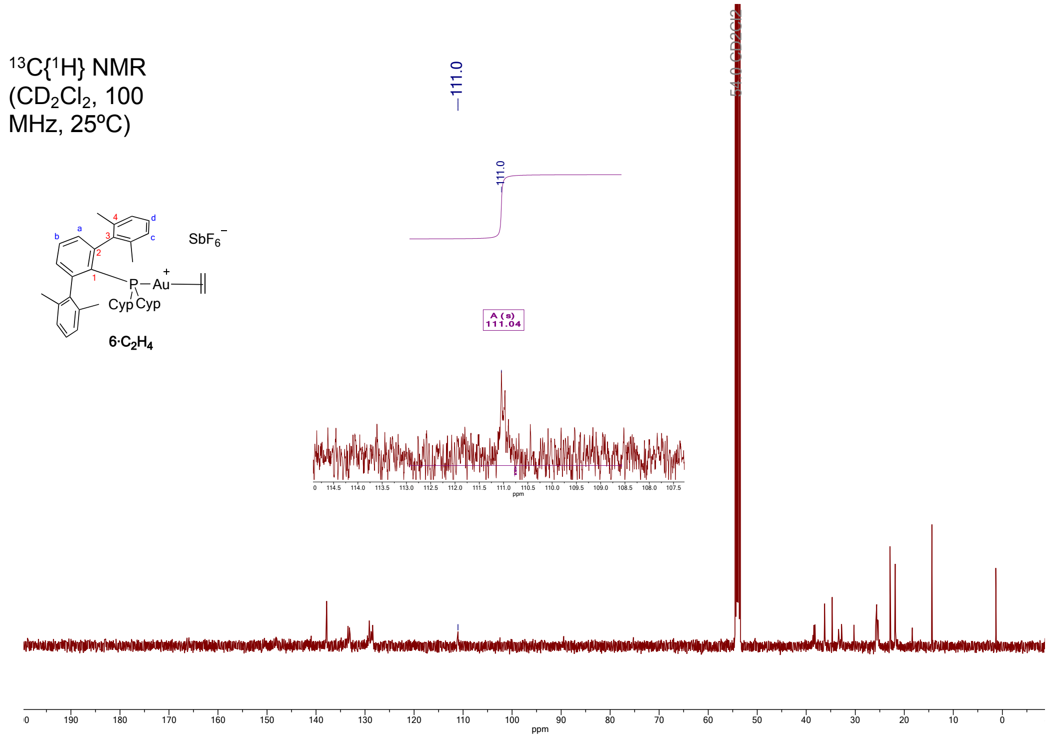

Figure S33. $^{13}C\{^1H\}$ NMR of complex **6·C₂H₄** with inset of the coordinated ethylene region at 25 ºC.

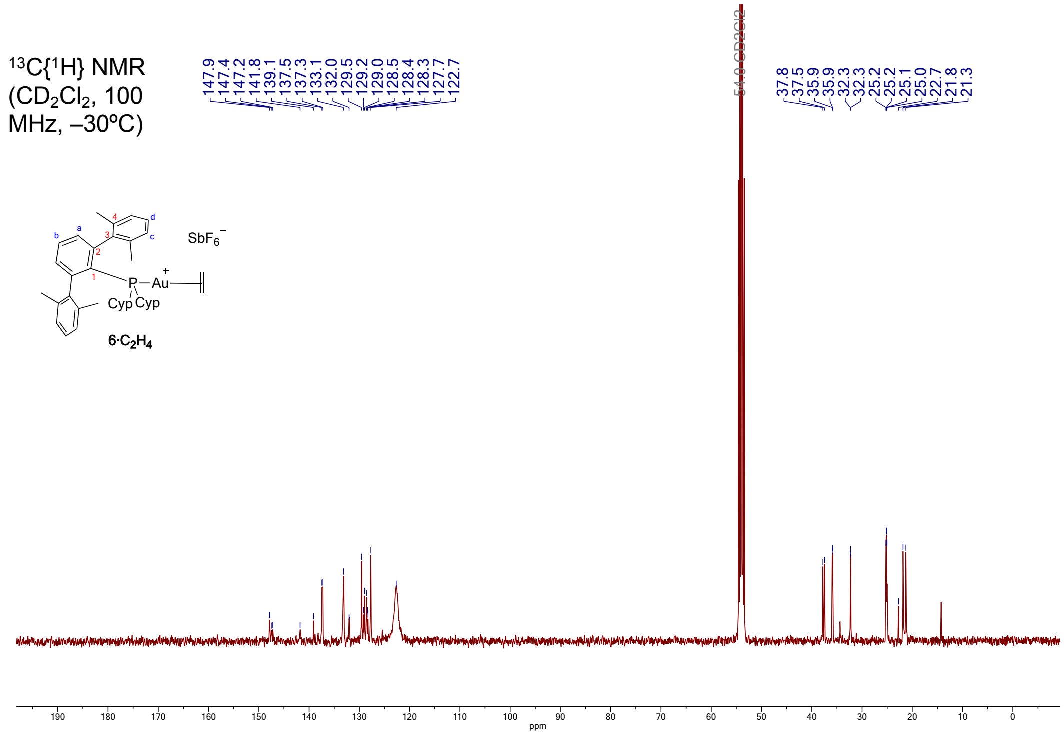

Figure S34. $^{13}C\{^1H\}$ NMR of complex **6·C₂H₄** in excess of ethylene at –30 ºC.

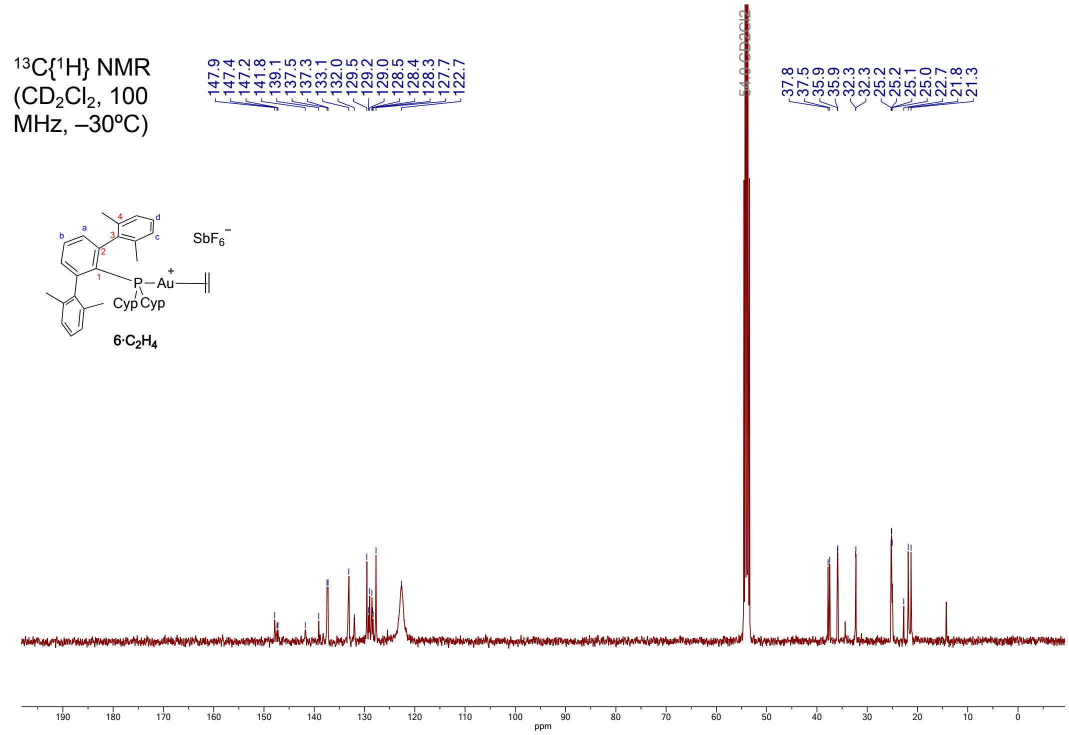

Figure S35. $^{31}P\{^1H\}$ NMR of complex **$6·C_2H_4$**.

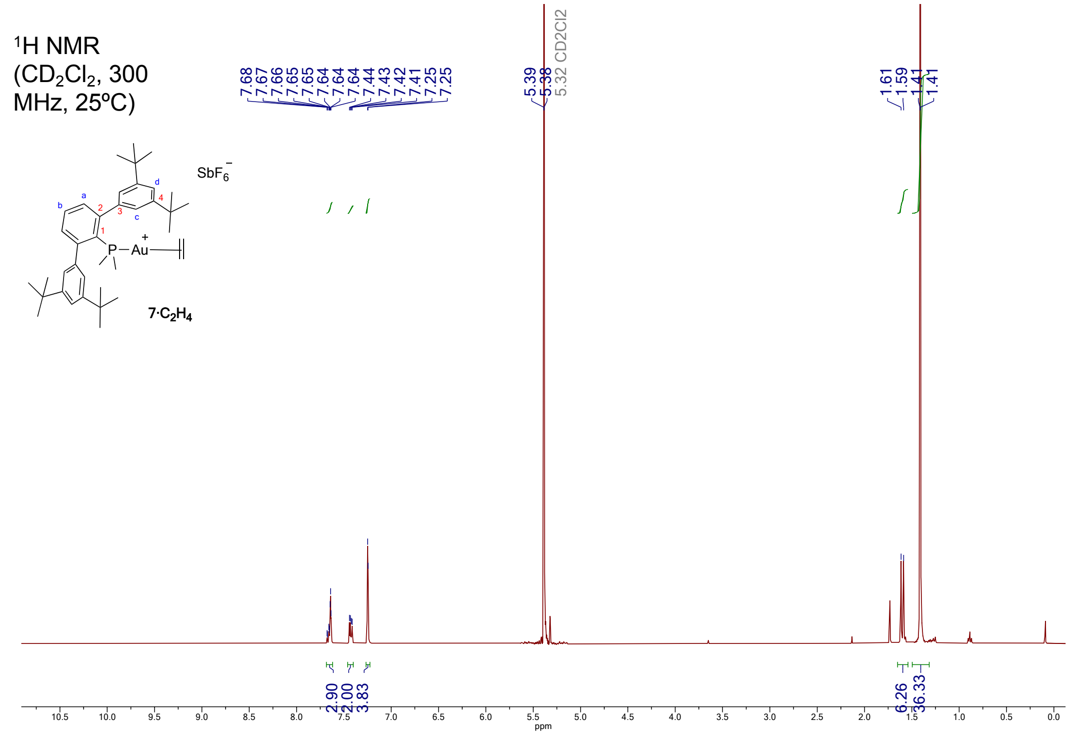

Figure S36. ^1H NMR of complex **$7·C_2H_4$** in excess of ethylene at 25 ºC.

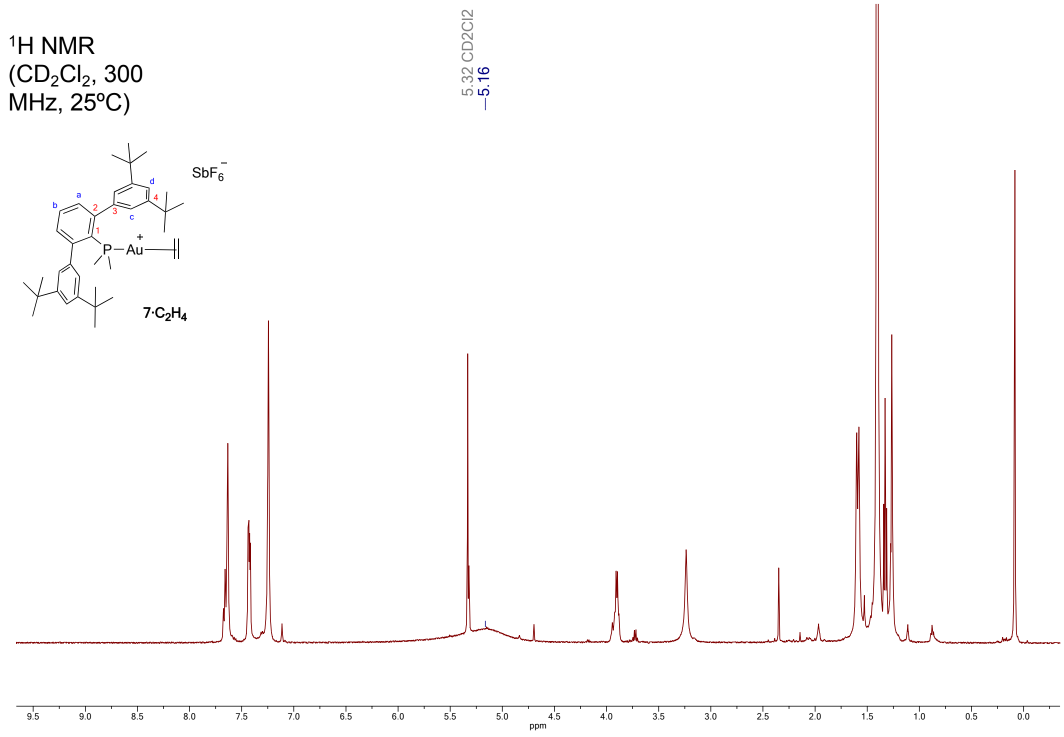

Figure S37. ^{1}H NMR of complex **7·C_2H_4** with coordinated ethylene selected 25 ºC.

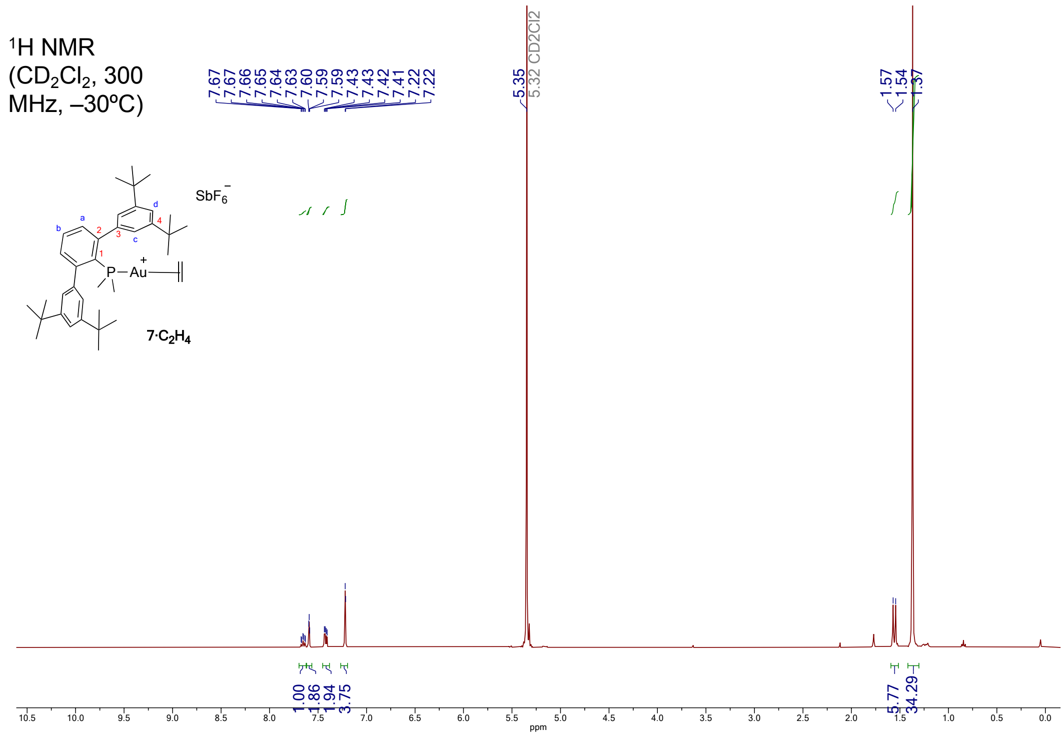

Figure S38. ^{1}H NMR of complex **7·C_2H_4** in excess of ethylene at –30 ºC.

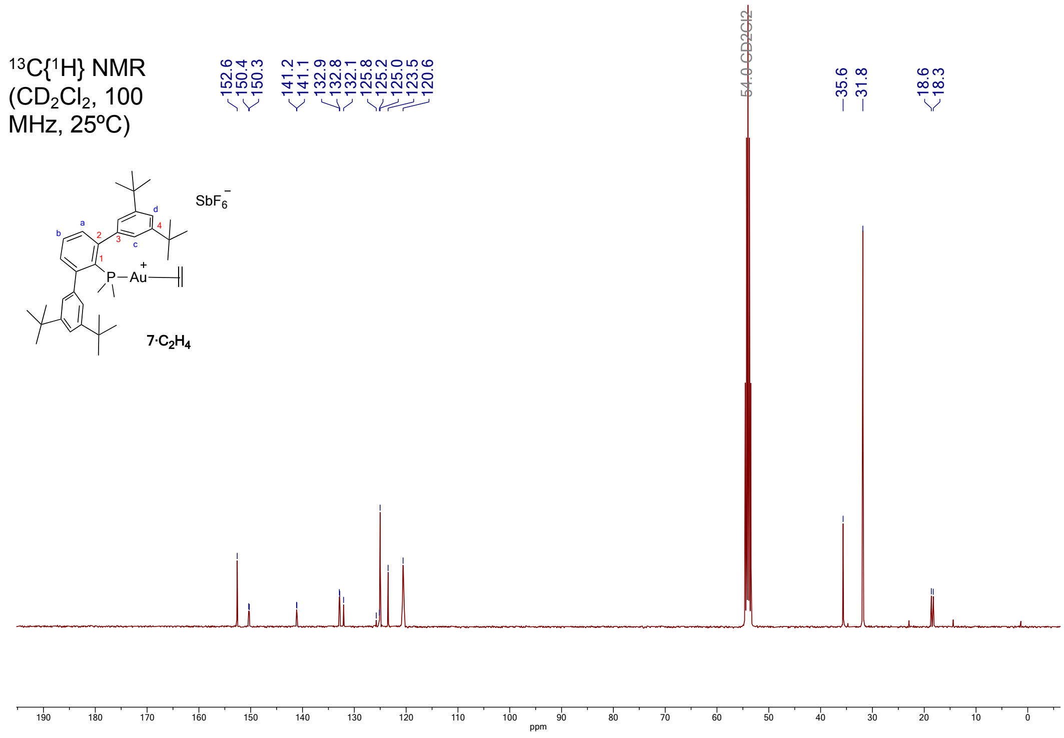

Figure S39. $^{13}C\{^1H\}$ NMR of complex **7·C_2H_4** in excess of ethylene at 25 ºC.

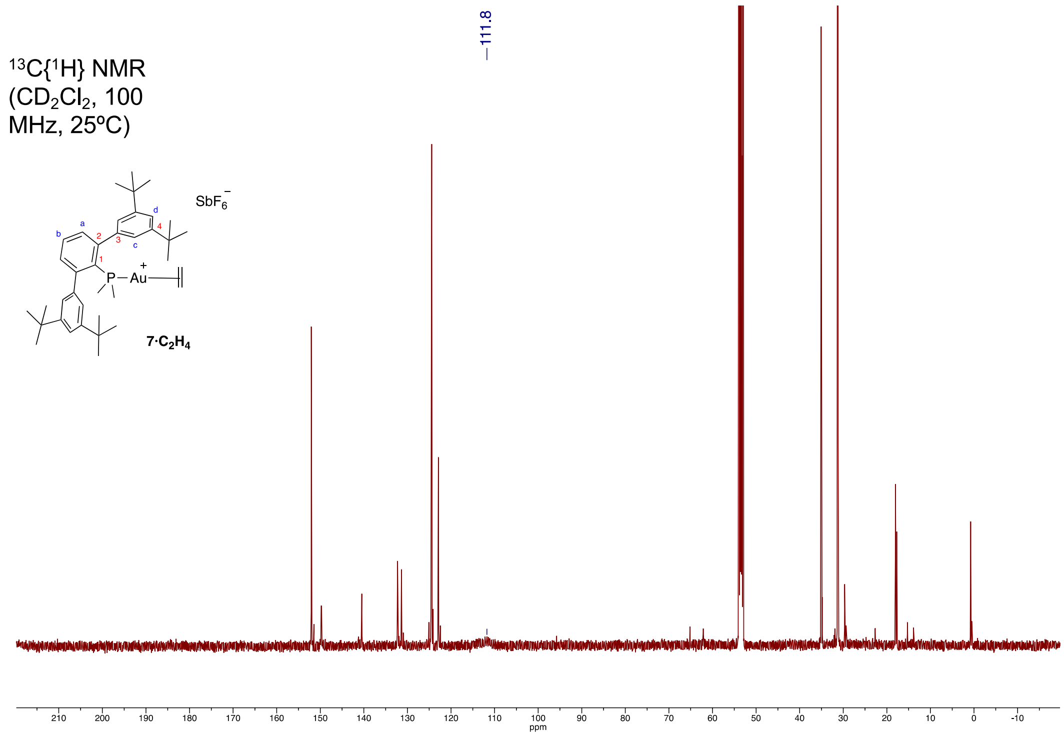

Figure S40. $^{13}C\{^1H\}$ NMR of complex **7·C_2H_4** with coordinated ethylene selected at 25 ºC.

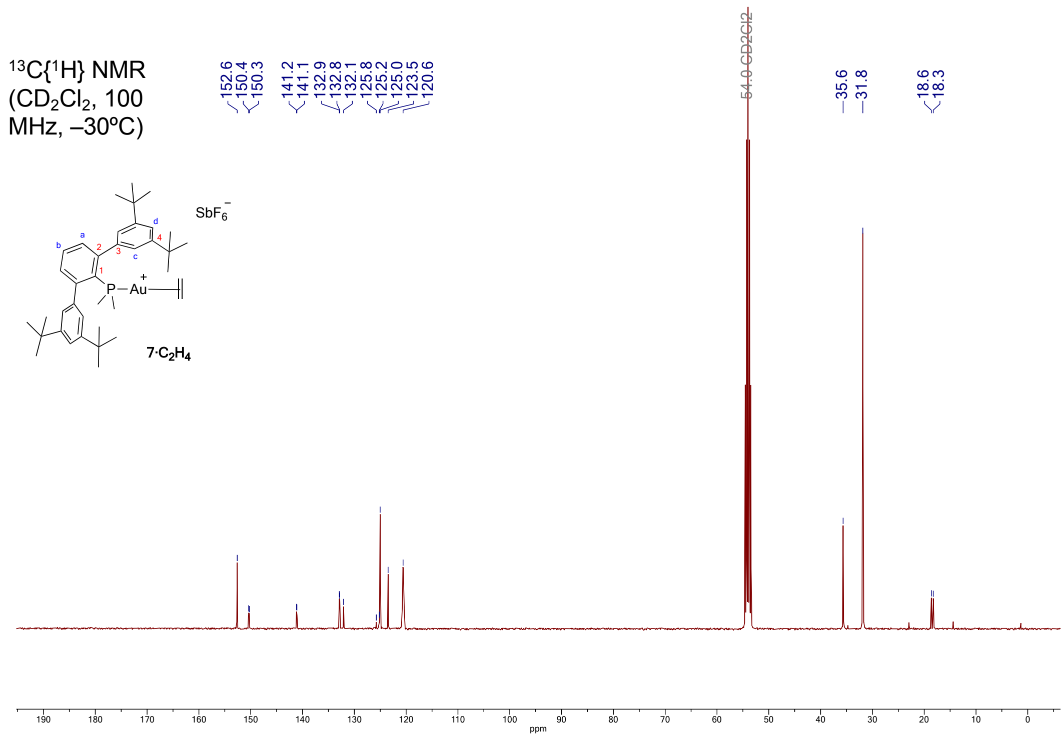

Figure S41. $^{13}C\{^1H\}$ NMR of complex **$7{\cdot}C_2H_4$** in excess of ethylene at –30 ºC.

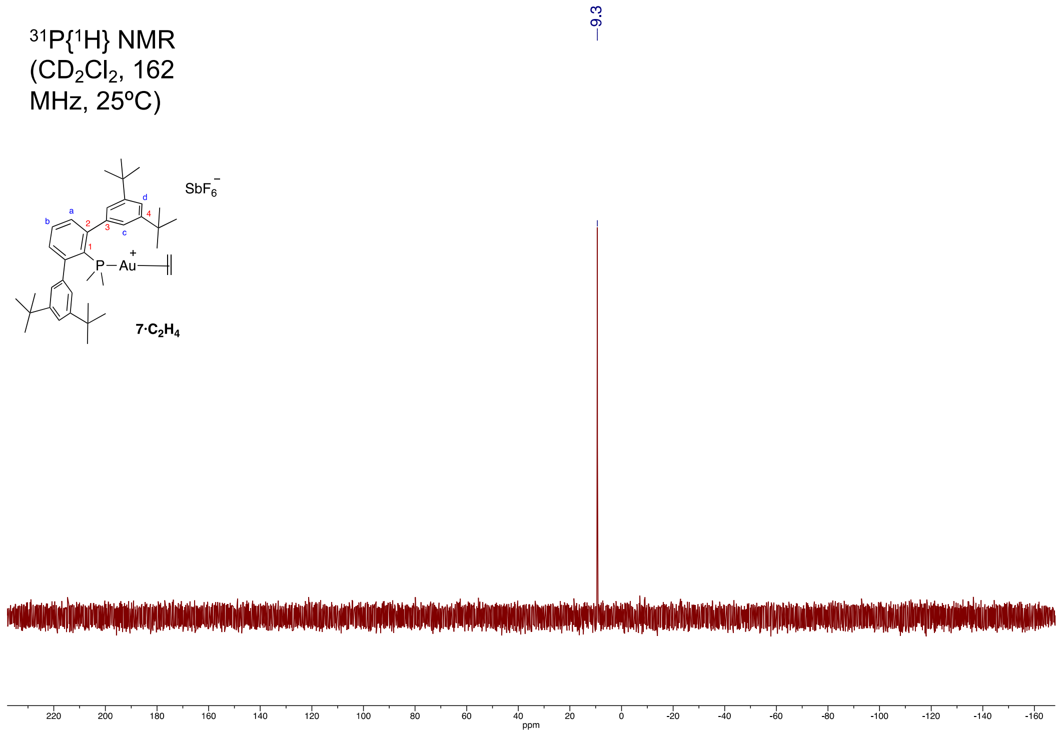

Figure S42. $^{31}P\{^1H\}$ NMR of complex **$7{\cdot}C_2H_4$**.

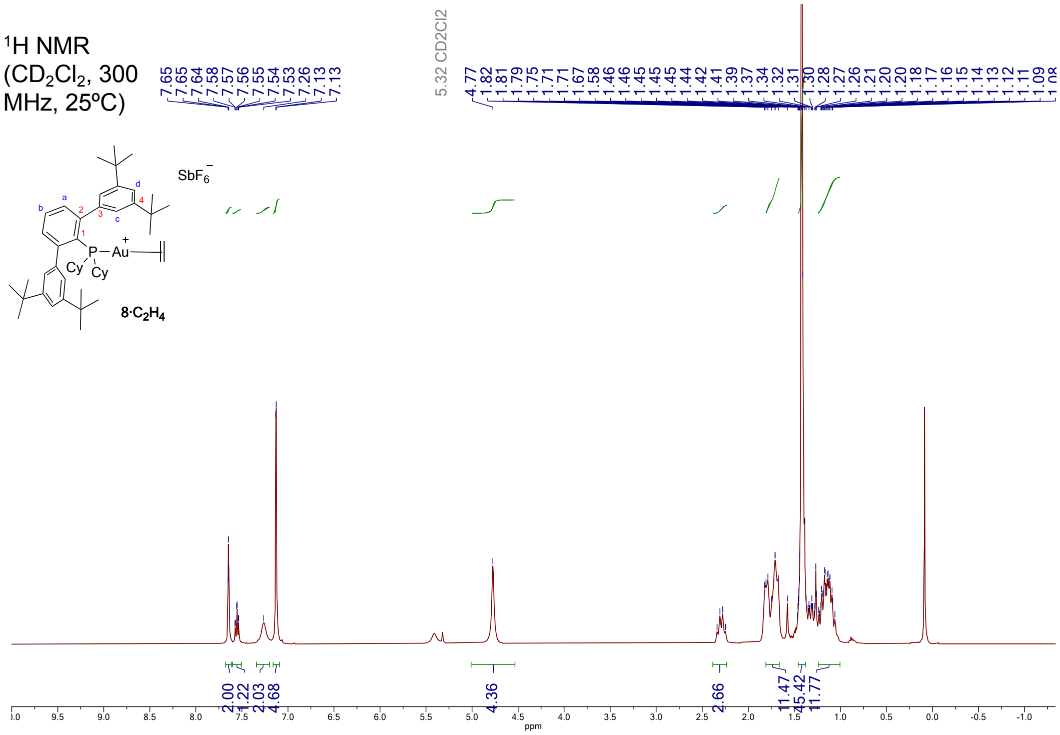

Figure S43. ^{1}H NMR of complex **8·C$_2$H$_4$** in excess of ethylene at 25 ºC.

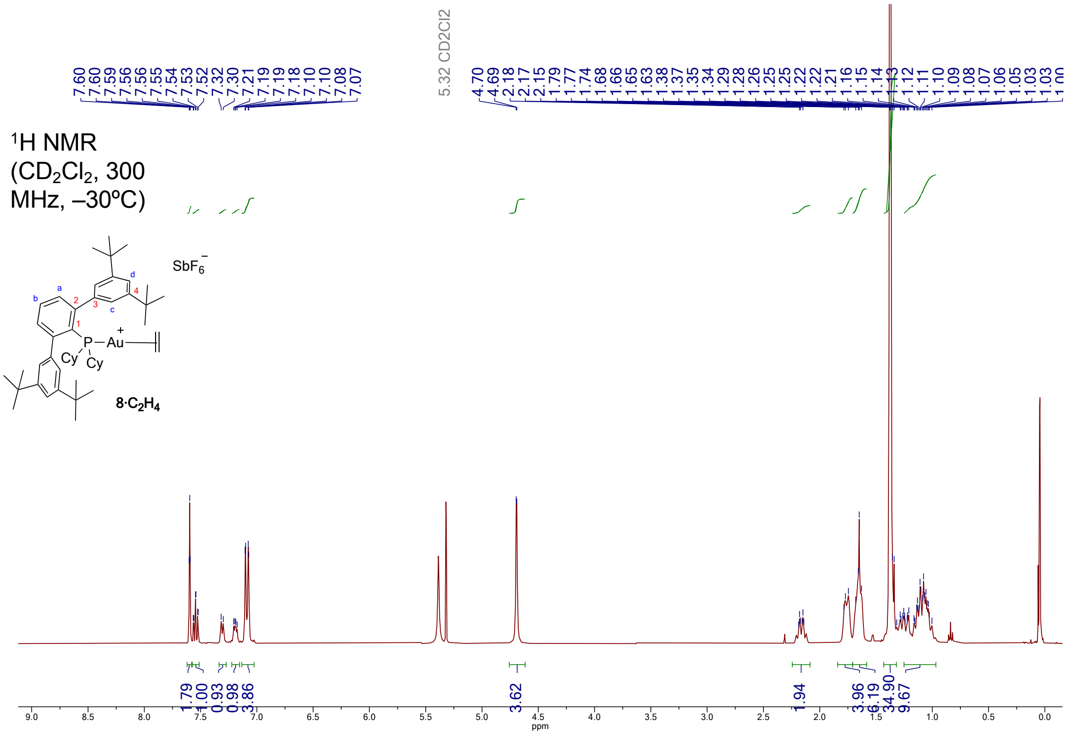

Figure S44. ^{1}H NMR of complex **8·C$_2$H$_4$** in excess of ethylene at –30 ºC.

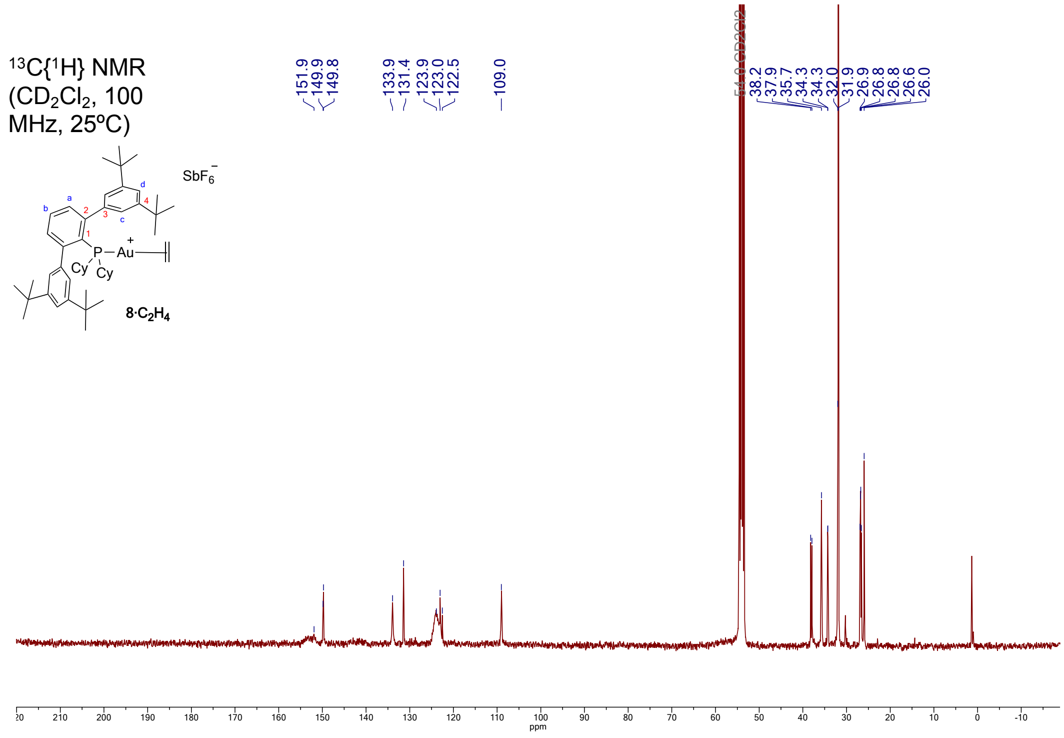

Figure S45. $^{13}C\{^1H\}$ NMR of complex $\mathbf{8{\cdot}C_2H_4}$ in excess of ethylene at 25 ºC.

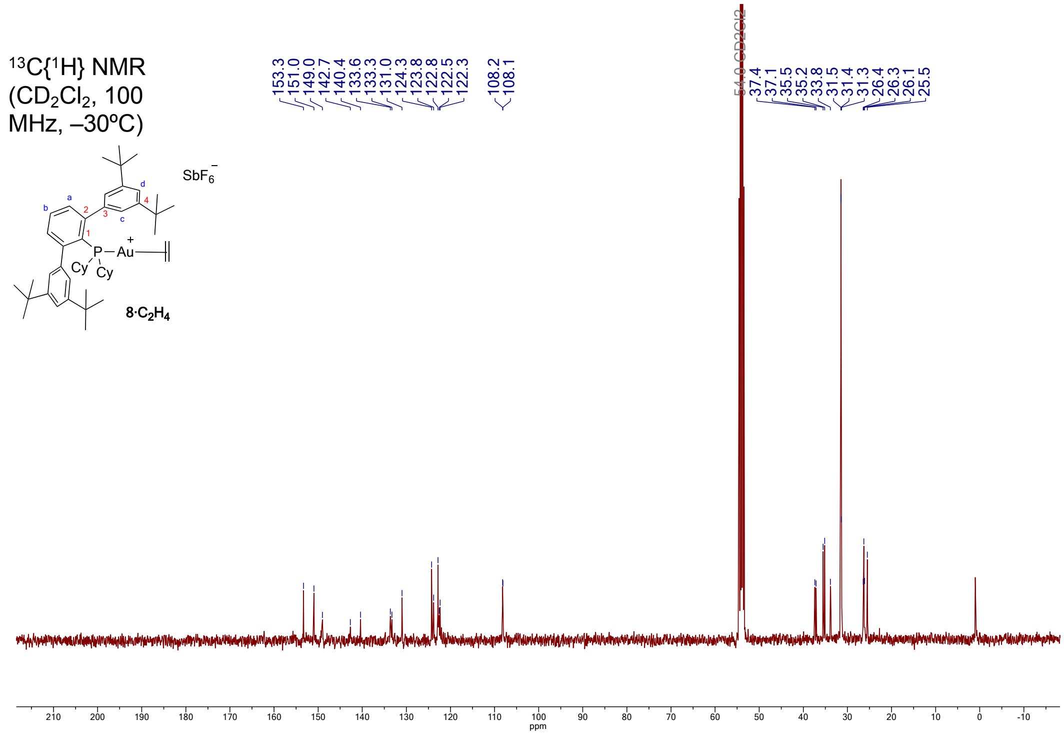

Figure S46. $^{13}C\{^1H\}$ NMR of complex $\mathbf{8{\cdot}C_2H_4}$ in excess of ethylene at –30 ºC.

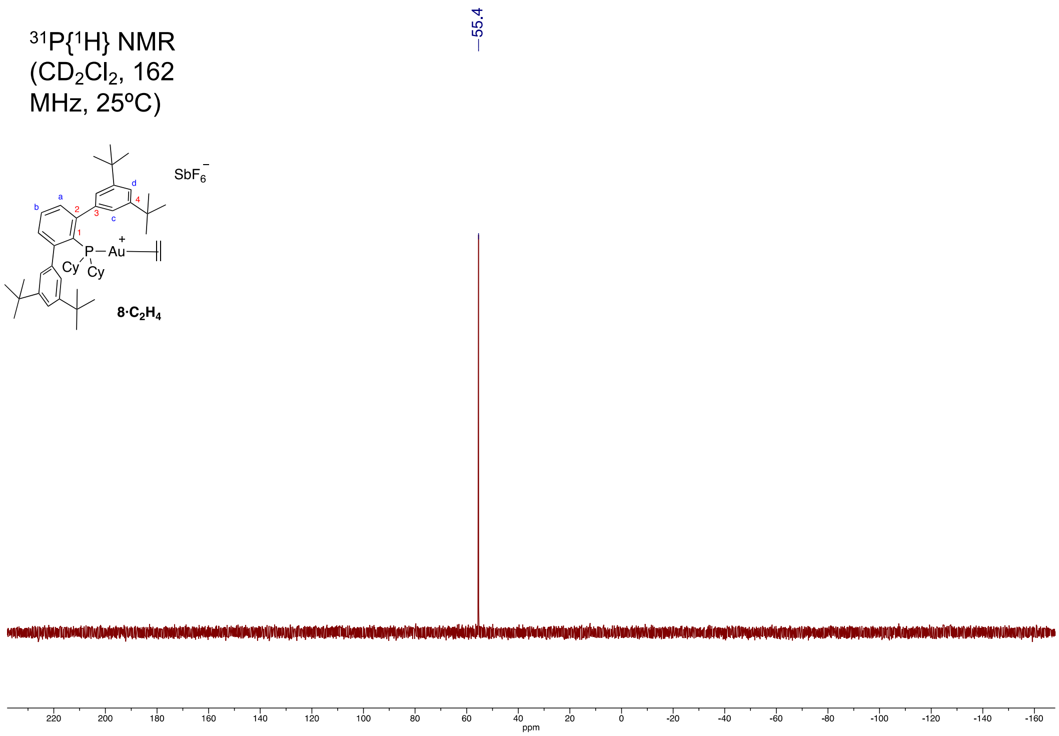

Figure S47. $^{31}P\{^{1}H\}$ NMR of complex **8·C₂H₄**.

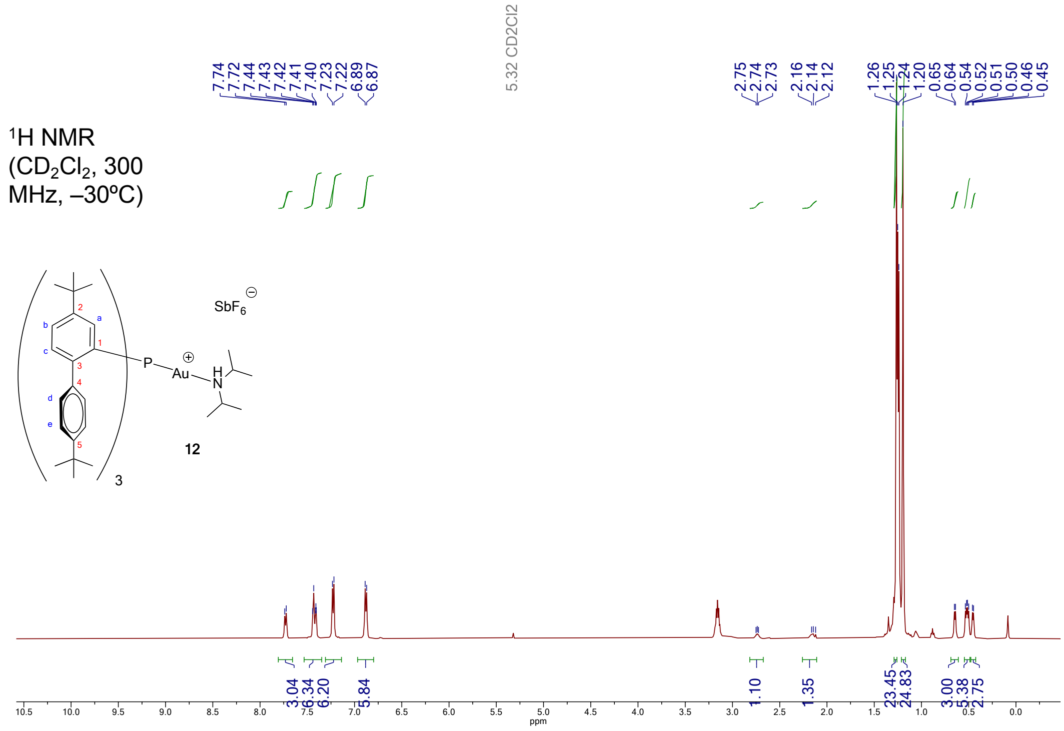

Figure S48. ^{1}H NMR of complex **12**.

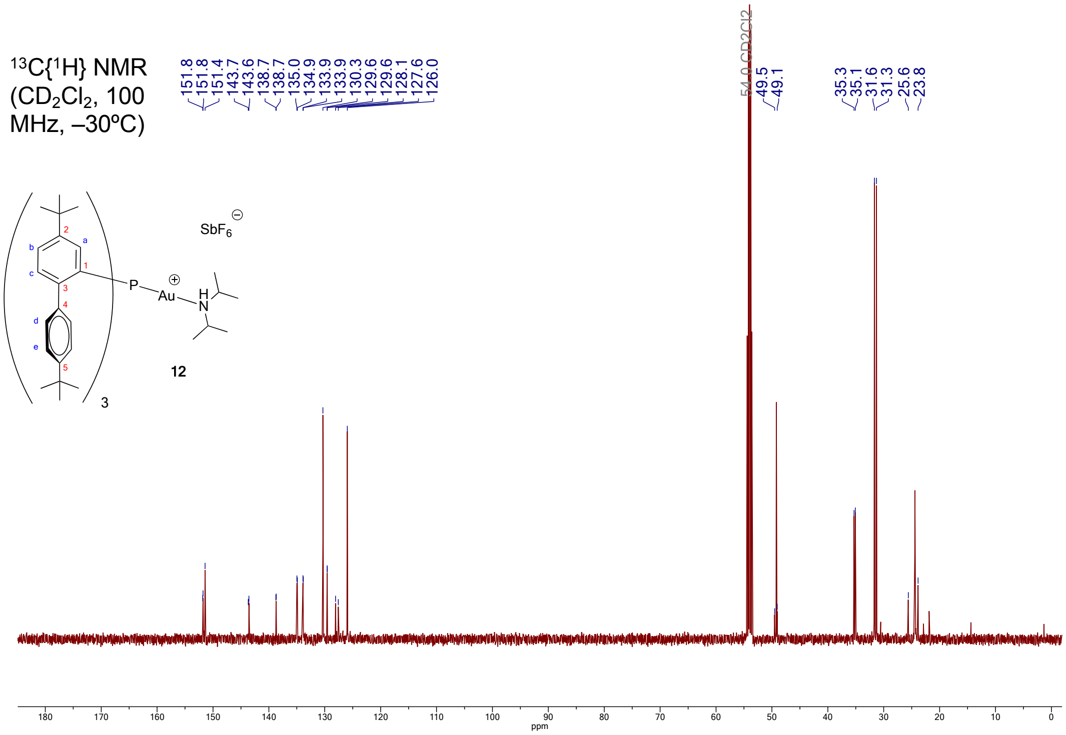

Figure S49. $^{13}C\{^1H\}$ NMR of complex **12**.

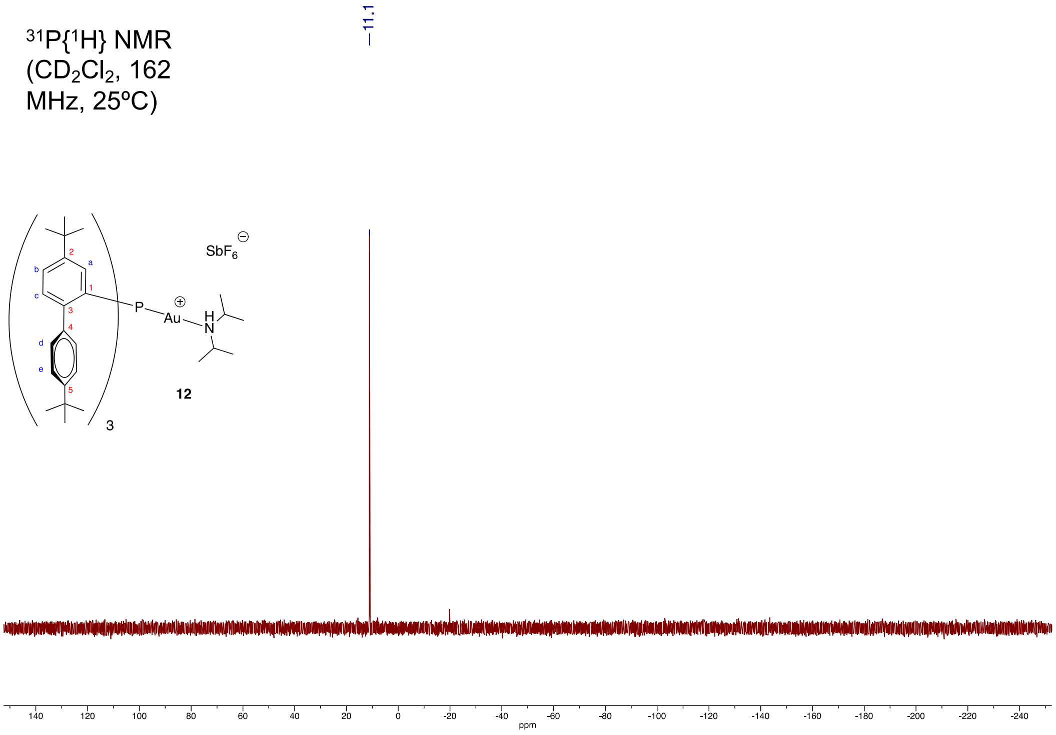

Figure S50. $^{31}P\{^1H\}$ NMR of complex **12**.

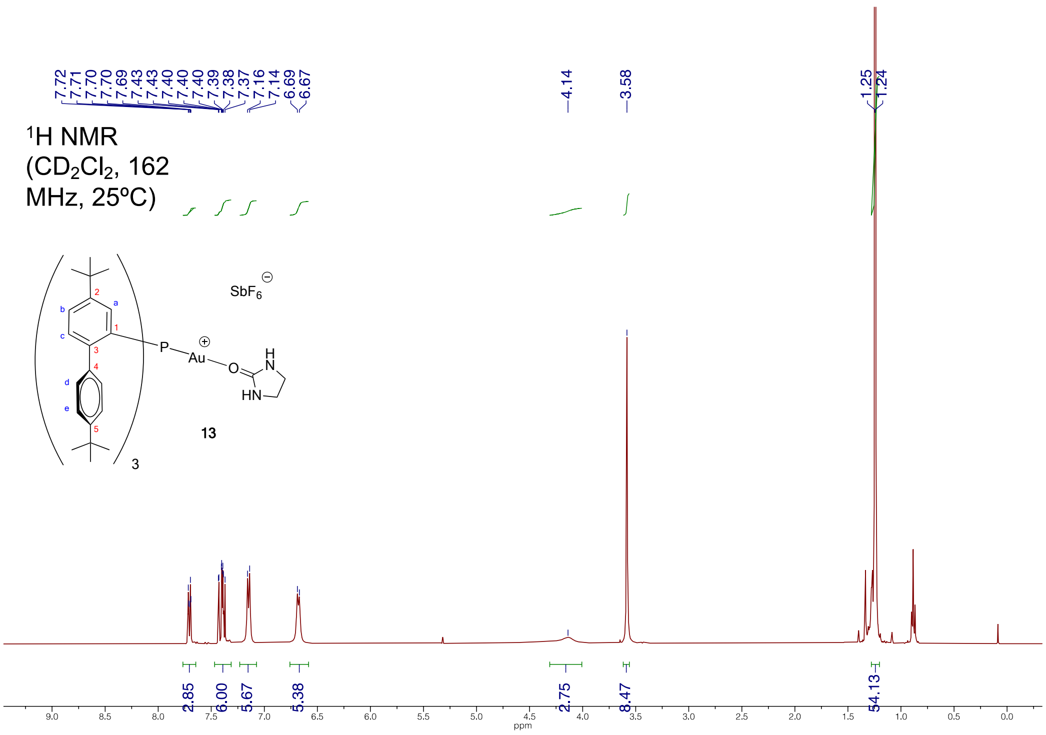

Figure S51. ^{1}H NMR of complex **13**.

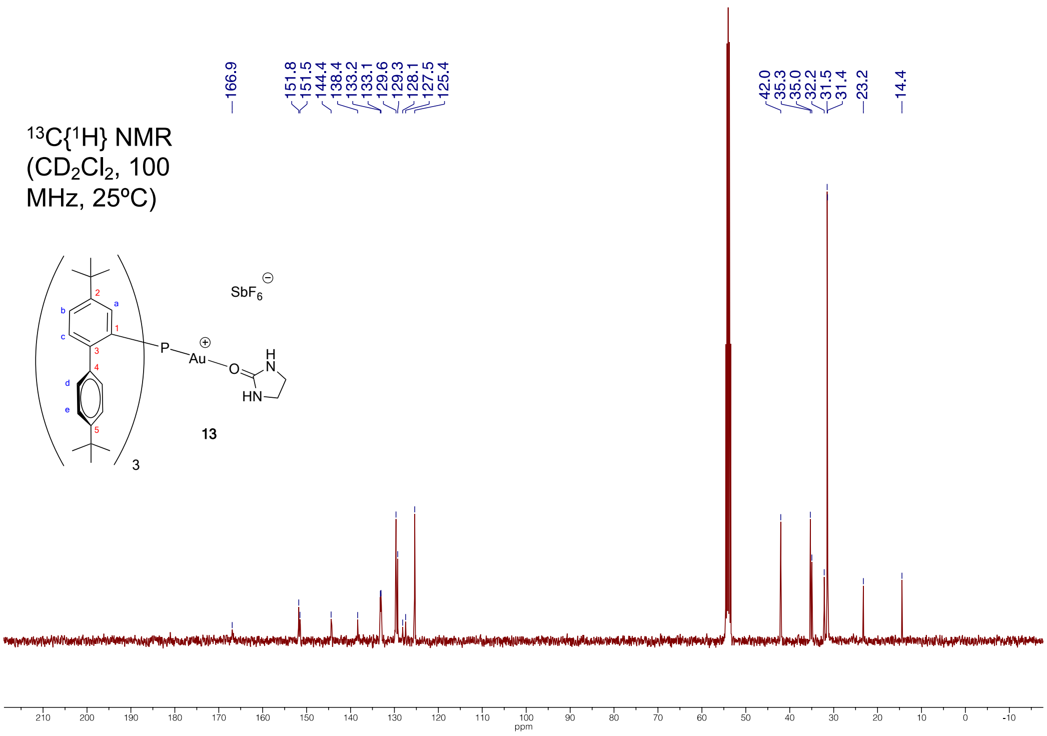

Figure S52. ^{13}C{^{1}H} NMR of complex **13**.

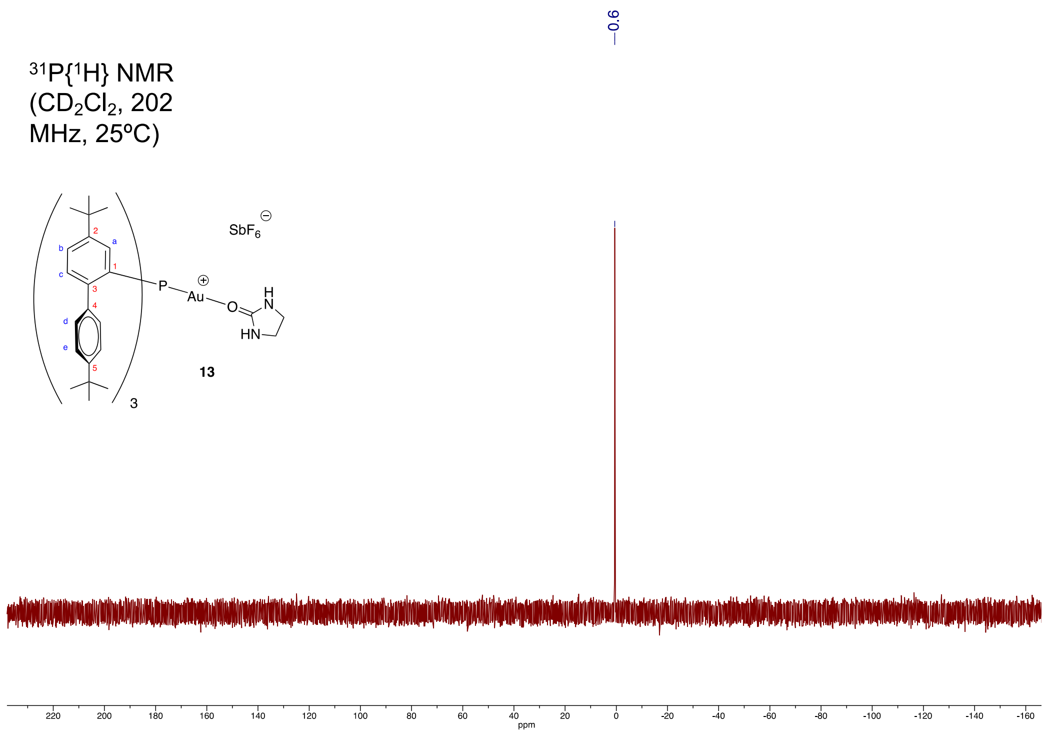

Figure S53. $^{31}P\{^{1}H\}$ NMR of complex **13**.

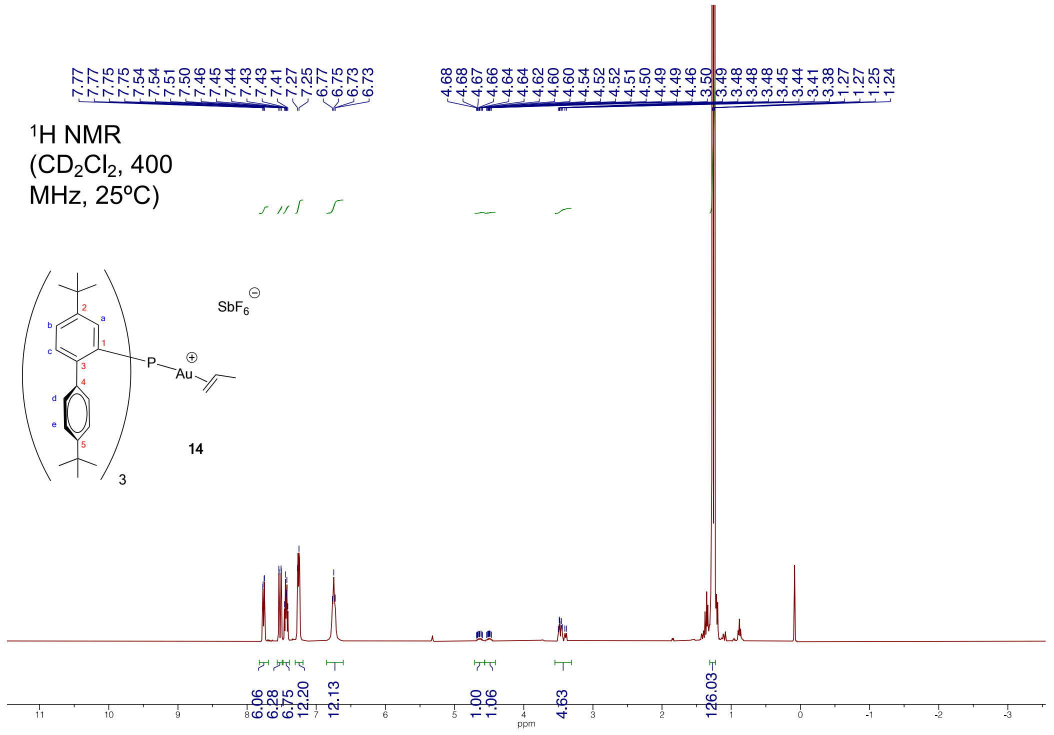

Figure S54. ^{1}H NMR of complex **14**.

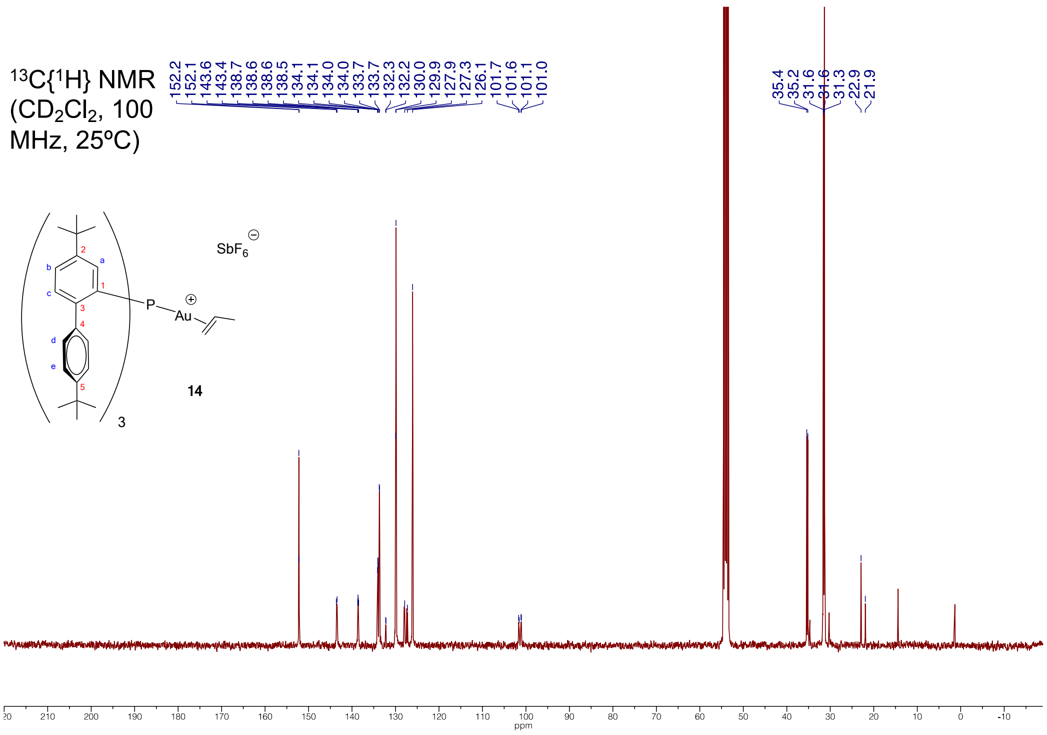

Figure S55. $^{13}C\{^1H\}$ NMR of complex **14**.

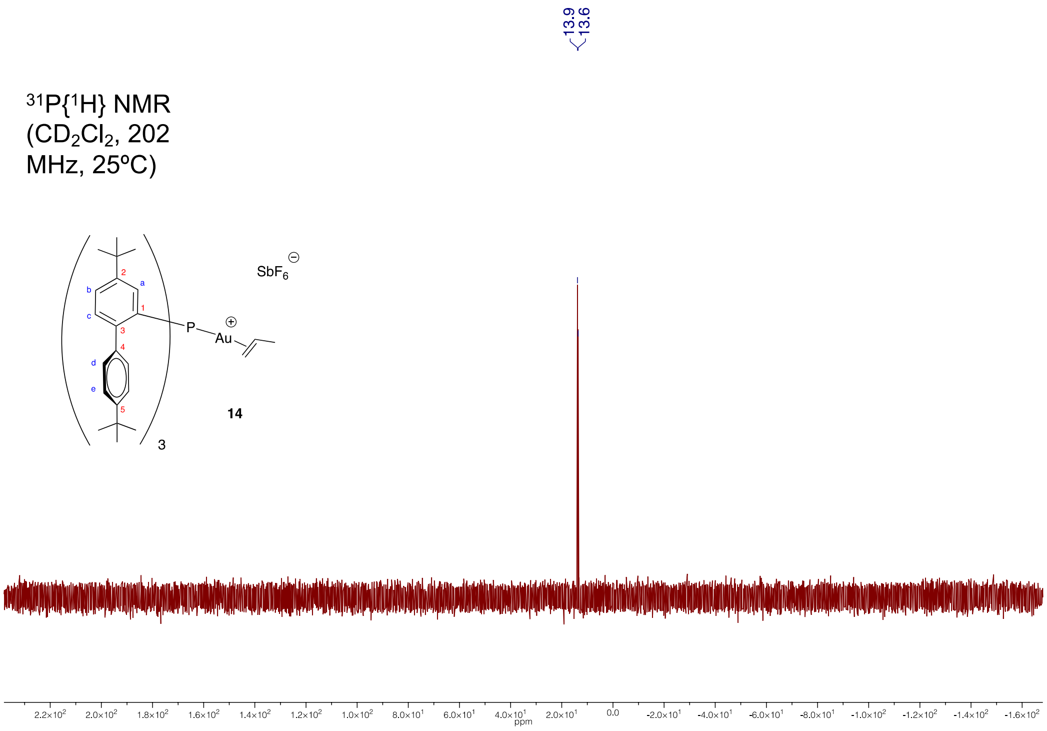

Figure S56. $^{31}P\{^1H\}$ NMR of complex **14**.

Chemical exchange between coordinated and free ethylene was observed in CD_2Cl_2 within the NMR timescale for all ethylene adducts, however, its rate could not be reliably quantified due to the rapid exchange and close proximity of the respective NMR signals, which prevented accurate data analysis. In figures S56–S60 are shown the NOE experiments recorded for complex **3·C₂H₄** in CD_2Cl_2 between 20 and –80 ºC, showing chemical exchange between coordinated and free ethylene.

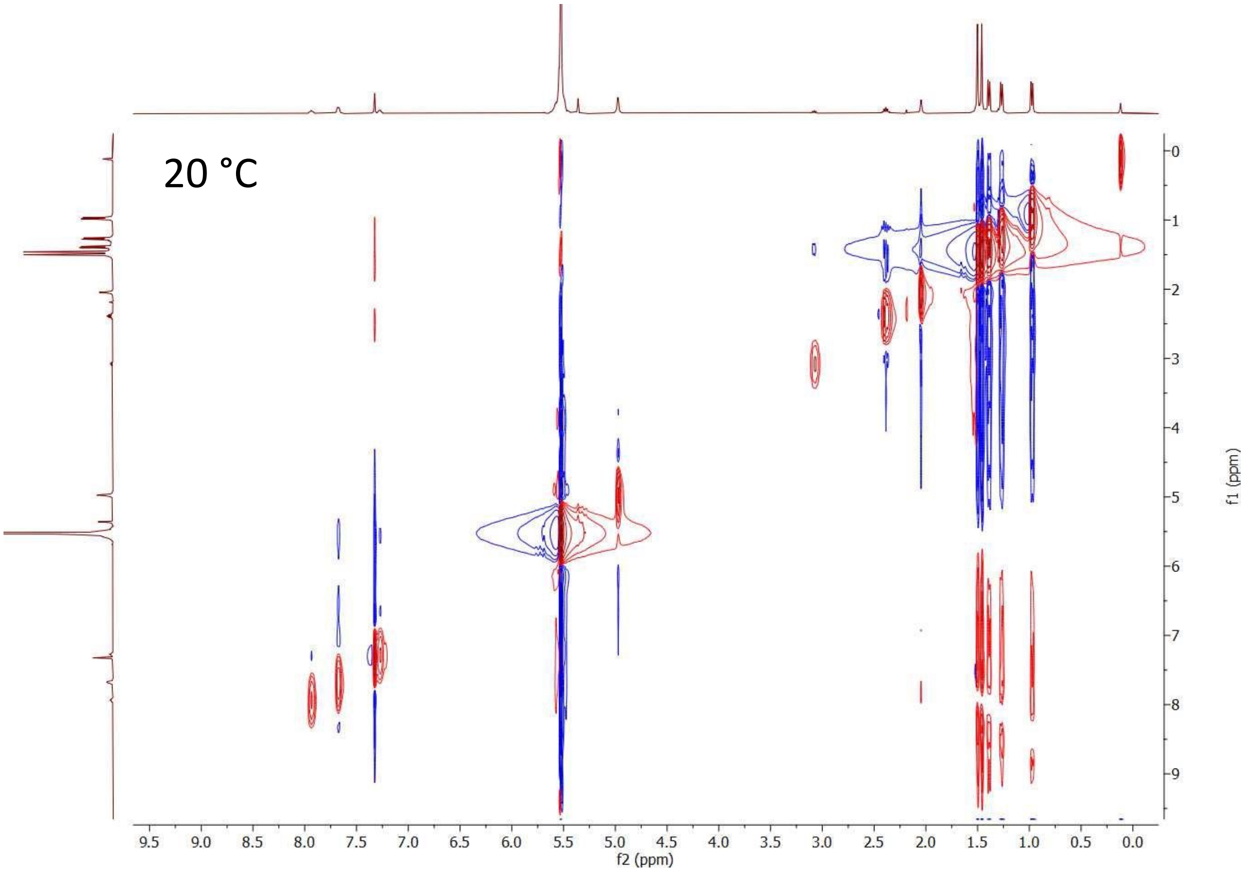

Figure S57. NOESY spectrum of complex **3·C_2H_4** at 20 ºC.

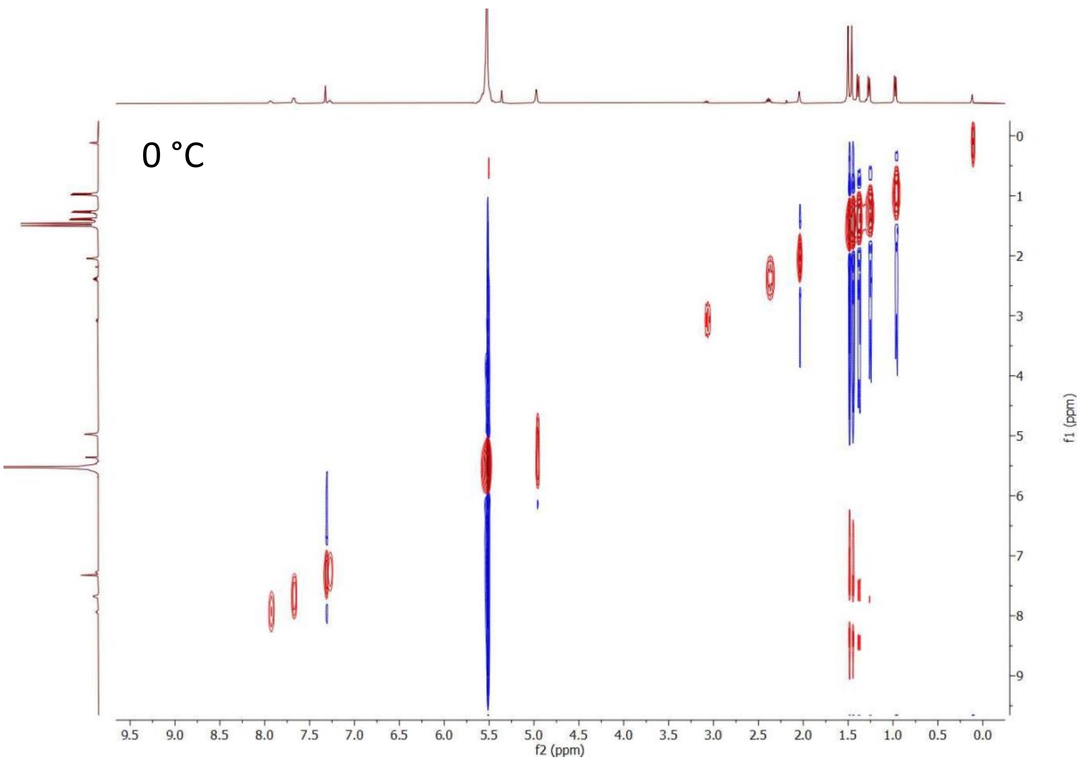

Figure S58. NOESY spectrum of complex $\mathbf{3{\cdot}C_2H_4}$ at 0 ºC.

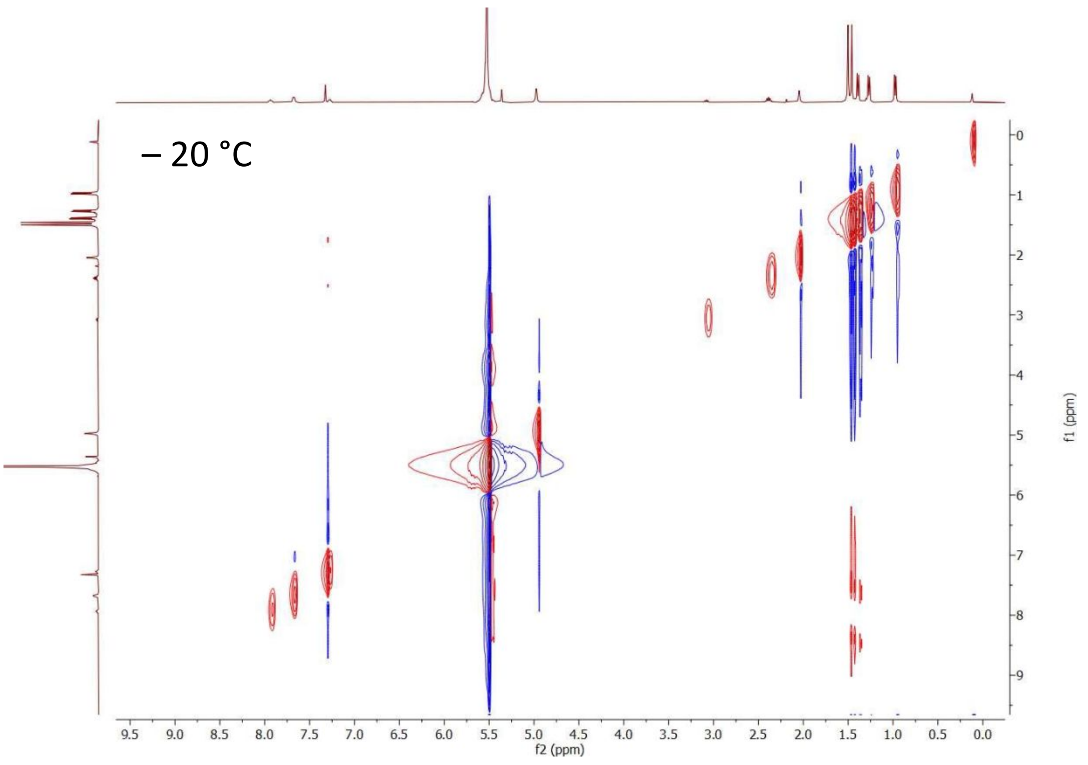

Figure S59. NOESY spectrum of complex $\mathbf{3{\cdot}C_2H_4}$ at –20 ºC.

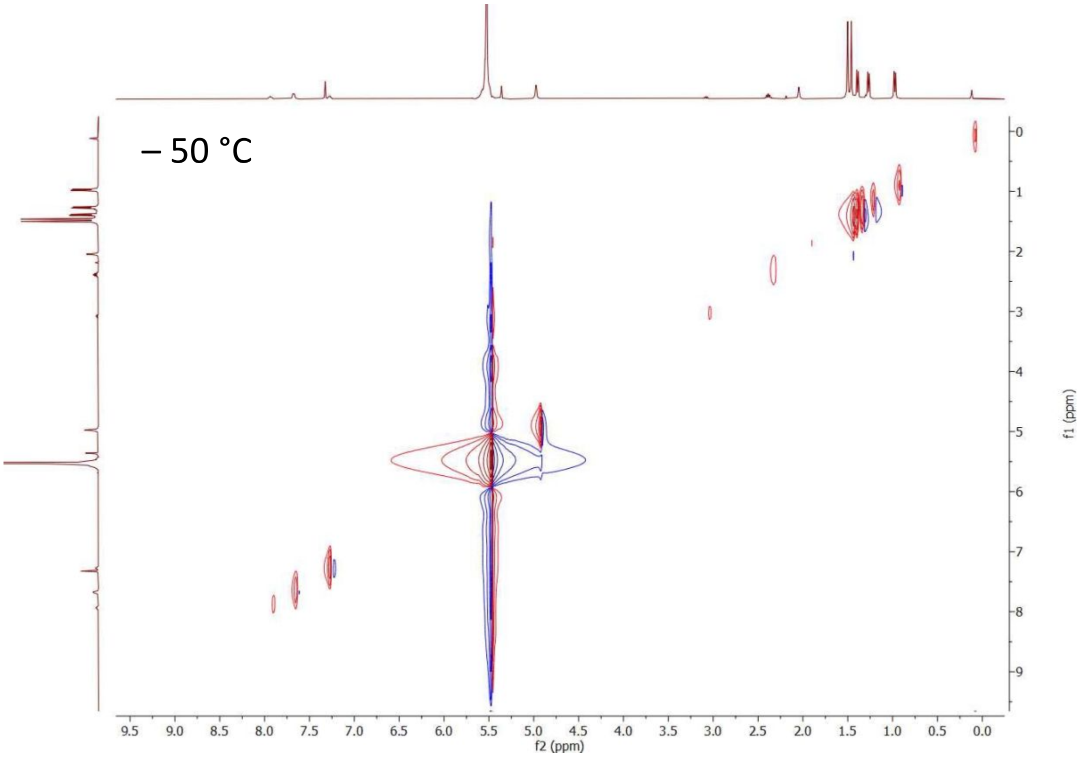

Figure S60. NOESY spectrum of complex **3·C$_2$H$_4$** at –50 ºC.

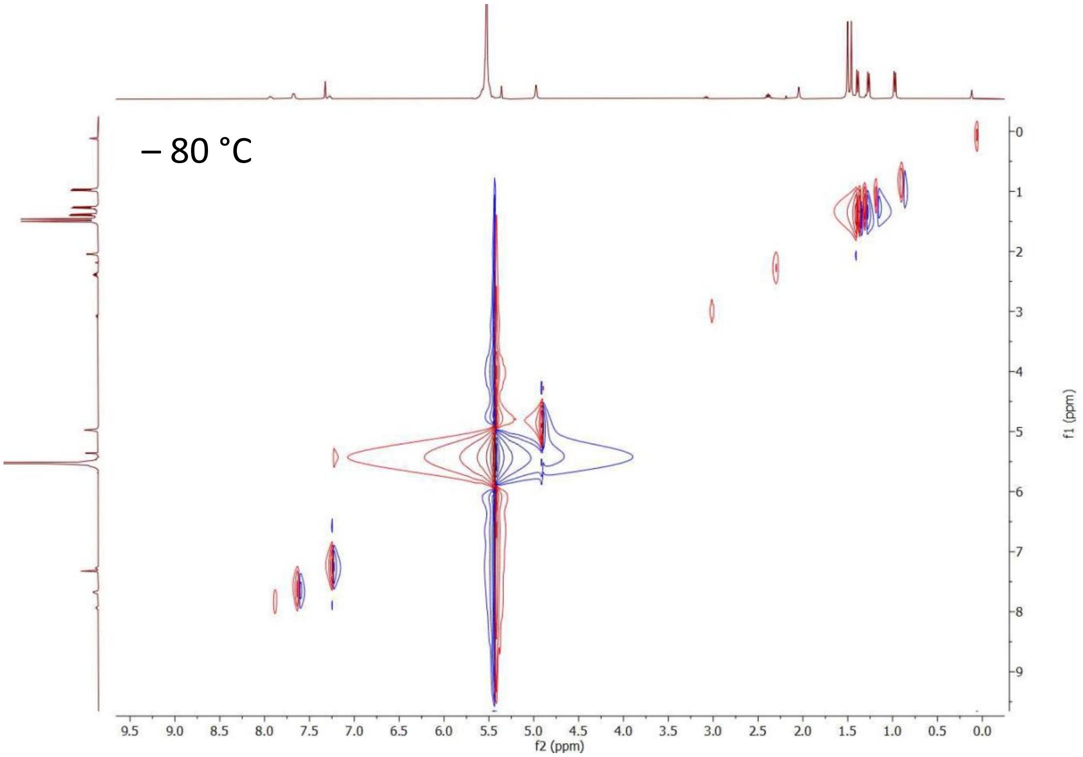

Figure S61. NOESY spectrum of complex **3·C$_2$H$_4$** at –80 ºC.

2. Formation of Au(I)-Ag(I) multimetallic species

Reaction of the gold(I) chloride complexes **3–8** with $AgSbF_6$ in the absence of ethylene atmosphere did not lead to instant precipitation of AgCl, arguing in favor of the presence of silver within the resulting structure. Complexes **3–8** bearing bulky biphenyl and terphenylyl phosphine ligands formed species characterized by broad NMR resonances that we tentatively attribute to gold(I)–silver(I) multimetallic complexes by analogy with our prior studies on compound **1**.[1]

The ^{1}H and $^{31}P\{^{1}H\}$ NMR spectra recorded at 25 °C of the resulting gold(I)–silver(I) multimetallic species derived from the reaction of complex **6** and $AgSbF_6$ is presented as an example:

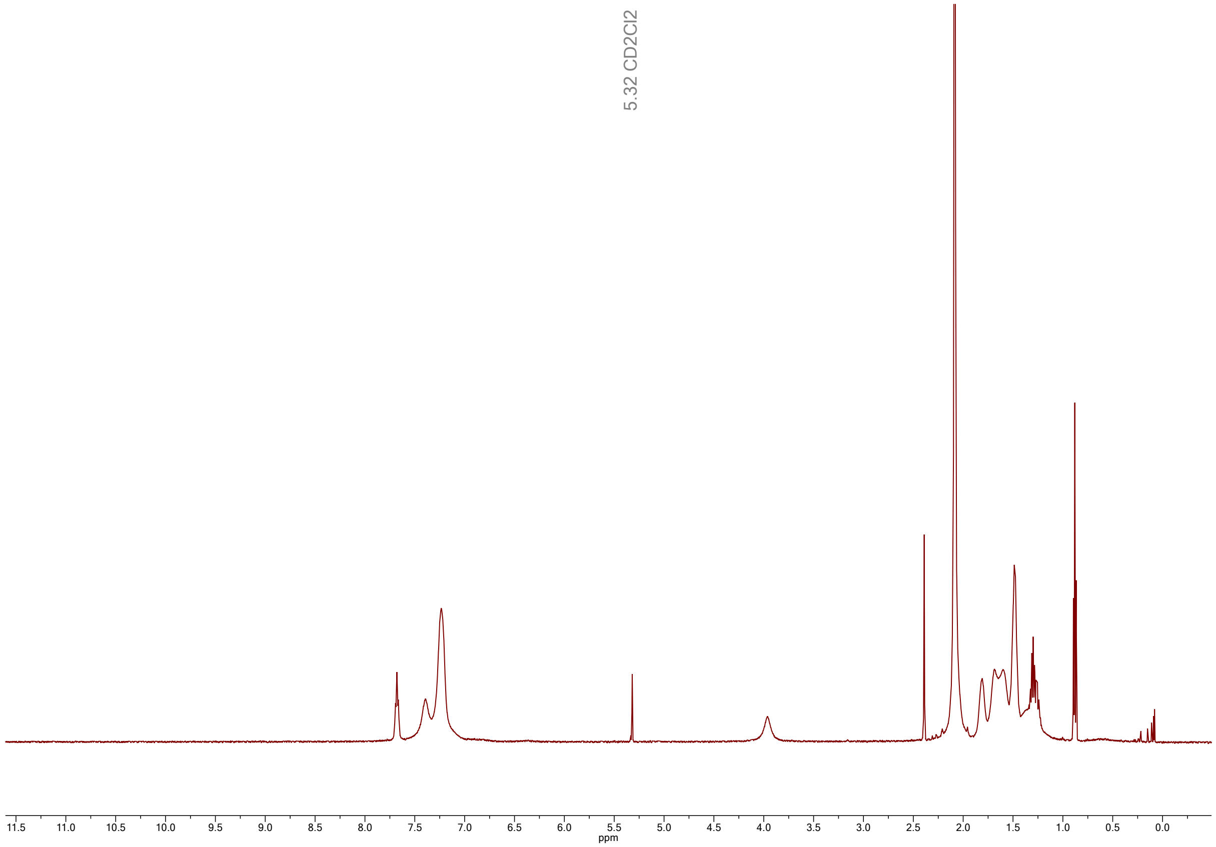

Figure S62. ^{1}H NMR of the Au(I)-Ag(I) multimetallic species derived from complex **6**.

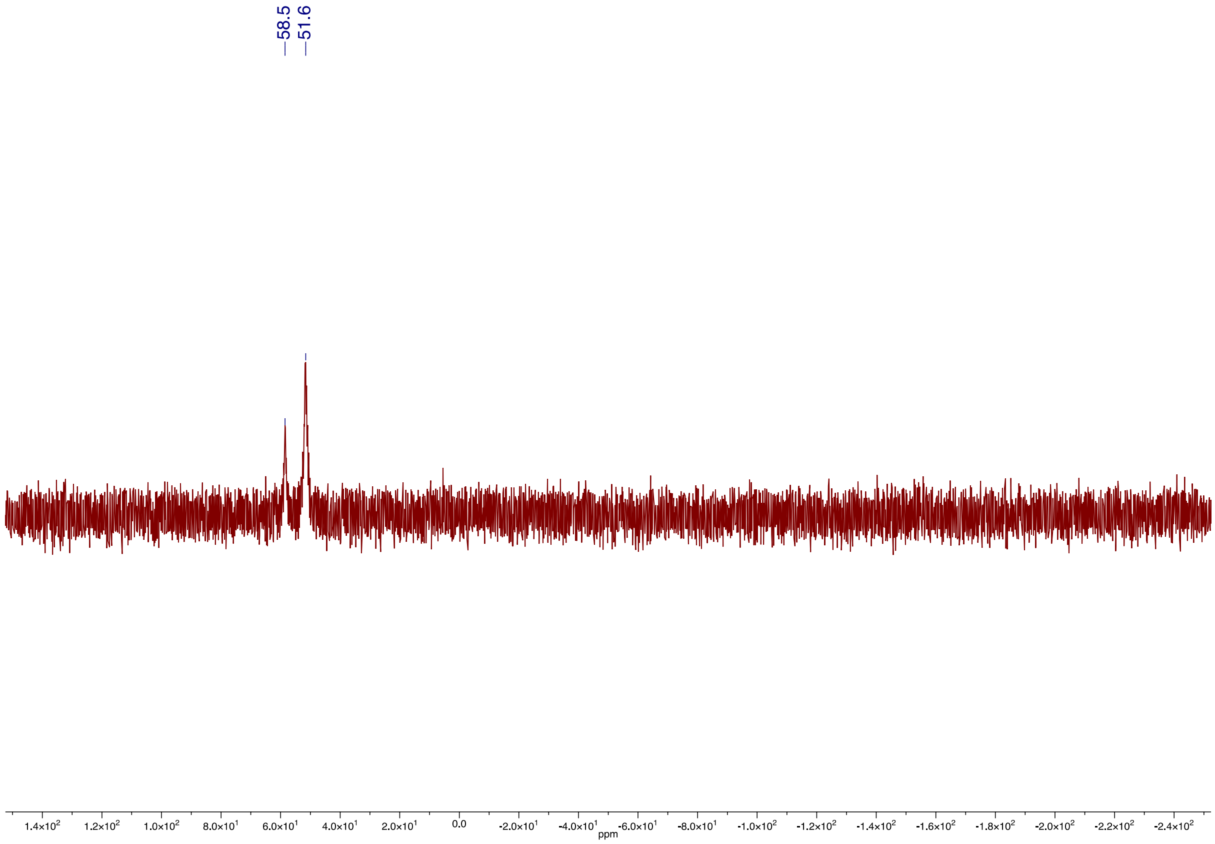

Figure S63. $^{31}P\{^1H\}$ NMR of the Au(I)-Ag(I) multimetallic species derived from complex **6**.

Diffusion-Ordered Spectroscopy (DOSY) was performed to further support the notion of the formation of gold(I)-silver(I) multimetallic species. For instance, ^1H DOSY experimental data revealed a diffusion coefficient for the in situ equimolar reaction between complex **6** and $AgSbF_6$ ($D = 9.13 \cdot 10^{-10}$ m/s^2) that accounts for only half of that for pure **6·C$_2$H$_4$** ($D = 1.75 \cdot 10^{-9}$ m^2/s), indicating a larger structure attributable to a multimetallic species in the former case.

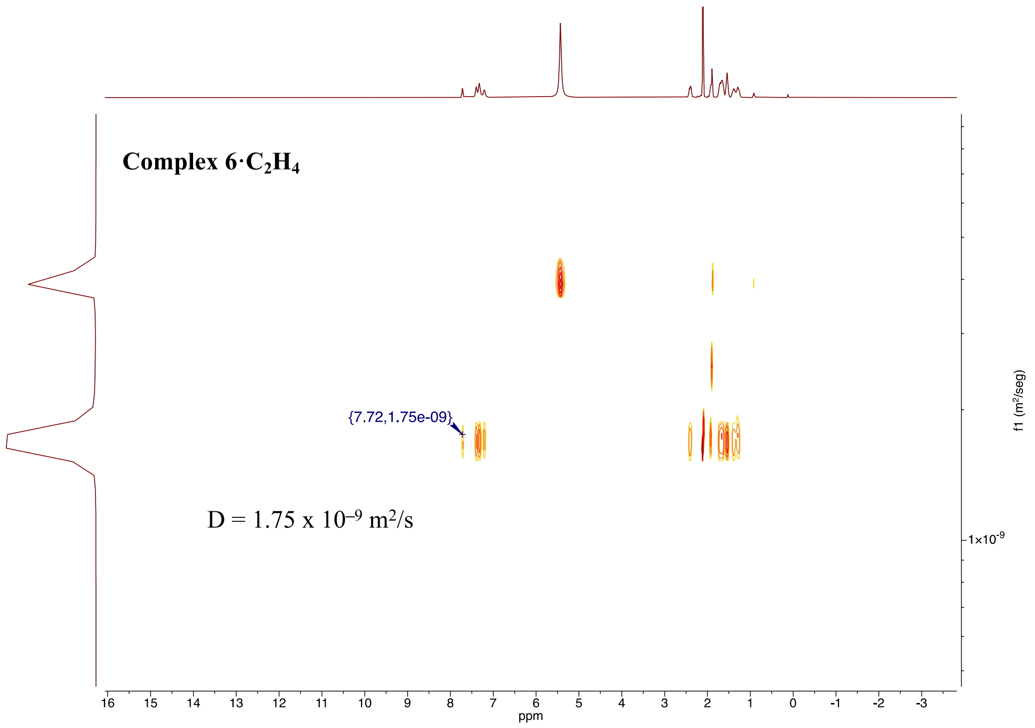

Figure S64. [1]H DOSY experiment for complex **6·C_2H_4** in CD_2Cl_2.

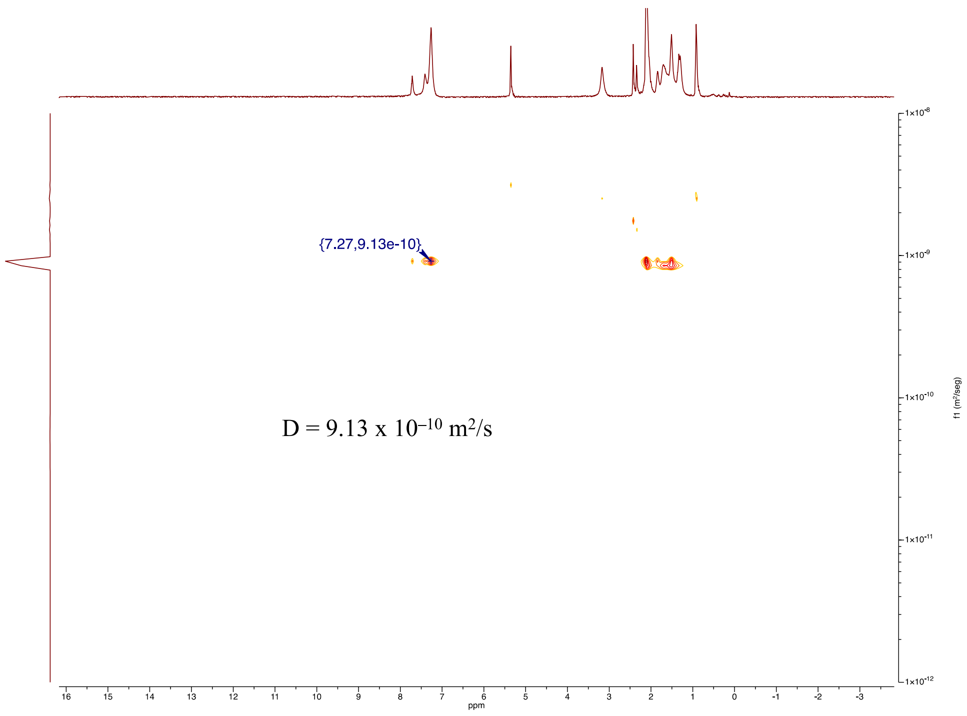

Figure S65. [1]H DOSY experiment for the gold(I)-silver(I) multimetallic species derived from complex **6** in CD_2Cl_2.

3. Formation of Au(I)-amine adducts

Different bulky amines were tested as substrates in the hydroamination of ethylene using gold(I) complexes **1** and **2**, but no conversion was observed. In these cases, new signals were detected in the $^{31}P\{^1H\}$ NMR spectra of the final mixtures that differ from the corresponding gold(I) chloride and gold(I) π-ethylene complexes. To confirm the ethylene displacement by the amines, stoichiometric reactions of complex **1·C₂H₄** and the corresponding amines in CD_2Cl_2 were monitored by $^{31}P\{^1H\}$ NMR spectroscopy showing full displacement in all cases, except for tetramethylpiperidine, which coordinates to gold substituting ethylene but without full conversion under otherwise identical conditions (2 h, 25 ºC).

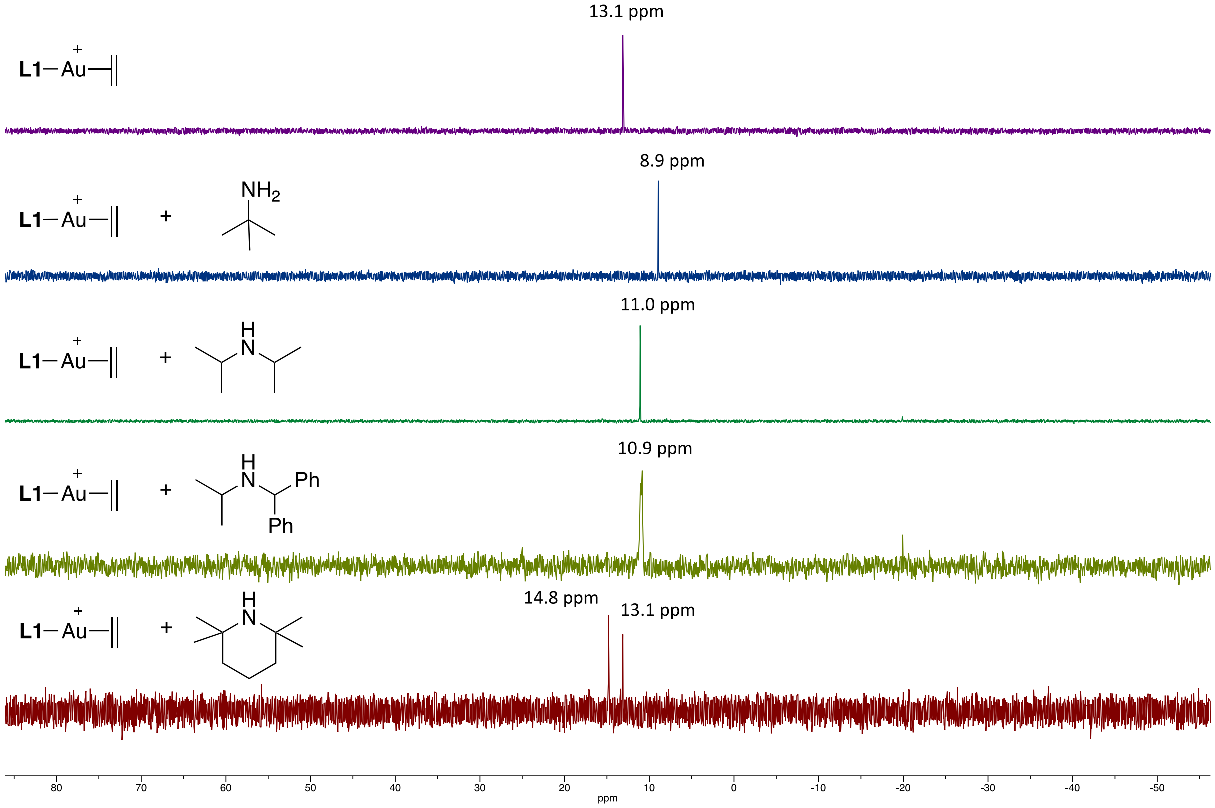

Figure S66. Comparison of the $^{31}P\{^1H\}$ NMR spectra of the Au(I)-amine adducts formed by the displacement of ethylene in complex **1·C$_2$H$_4$** by different amines.

4. Catalytic experiments.

4.1 Additive screening for the gold(I)-catalyzed hydroamination of ethylene

A mixture of imidazolidine-2-one (0.50 mmol), gold chloride complex (0.01 mmol), silver hexafluoroantimoniate (0.01 mmol) and the indicated additive in dioxane was placed in an ampoule with a magnetic stirring bar under nitrogen atmosphere. The ampoule was freeze-pumped to remove the nitrogen gas, filled with ethylene (1 bar) and stirred at 100 °C for 18 h. After this time, the mixture was cooled down to rt, diluted in CH_2Cl_2 (5 mL) and anisole (22 mL, 0.20 mmol) was added as internal standard. The mixture was then filtered through a short pad of celite, the solvents removed under reduced pressure and the sample analyzed by NMR spectroscopy in $CDCl_3$.

Table S1. Additive screeing in the intermolecular hydroamination of ethylene with complex **1** and **3**.[a]

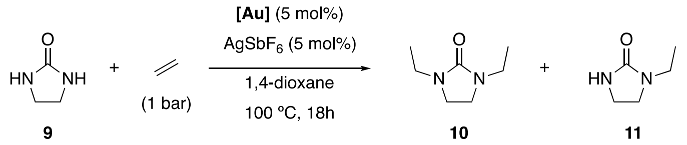

Entry	Catalyst	Additive	Conversion (%)[b]
1	1	-	64
2	3		50
3	1	H_2O (10 mol%)	49
4	1	H_2O (10 equiv)	67
5	3	H_2O (10 mol%)	49
6	3	H_2O (10 equiv)	65
7	1	HOTf (10 mol%)	<5
8	1	HOTf (10 equiv)	<5
9	1	CH_3COOH (10 mol%)	45
10	1	CH_3COOH (10 equiv)	40
11	1	HFIP (10 mol%)	65
12	1	HFIP (10 equiv)	35
13	1	^{t}BuOK (10 mol%)	-
14	1	^{t}BuOK (10 equiv)	-
15	1	Et_3N (10 mol%)	<5
16	1	Et_3N (10 equiv)	-
17	1	DBU (10 mol%)	-
18	1	DBU (10 equiv)	-

[a]Reaction was performed with imidazolidine-2-one (0.50 mmol) under the 1 bar of ethylene pressure, gold catalyst (0.01 mmol) and $AgSbF_6$ (0.01 mmol) as chloride abstractor in 1,4-dioxane (1 mL) at 100 °C for 18 h. [b]Conversion was determined by ^1H NMR spectroscopy with anisole as the internal standard.

4.2 Gold(I)-catalyzed hydroamination of alkenes

General procedure for the gold(I)-catalyzed hydroamination of alkenes. A mixture of amide (0.20 mmol), gold chloride complex (0.01 mmol), silver hexafluoroantimoniate (4 mg, 0.01 mmol) and the indicated alkane (15 eq., 2.00 mmol) in dioxane (1 mL) was placed in an ampoule together with a magnetic stirring bar under nitrogen atmosphere and stirred at 100 °C for 18 h. After this time, the mixture was cooled down to rt, diluted in CH_2Cl_2 (5 mL) and anisole (22 mL, 0.20 mmol) was added as internal standard. The mixture was then filtered through a short pad of celite, the solvents removed under reduced pressure and the sample analyzed by NMR spectroscopy in $CDCl_3$. For ethylene and propene a Fischer Porter tube was used. The tube was freeze-pumped to remove the nitrogen gas, filled with the indicated ethylene or propene pressure and stirred at 100 °C for 18 h. The work-up was analogous to the other alkenes.

Table S2. Intermolecular hydroamination of alkenes with complex **1**.[a]

Entry	Nucleophile	Alkene	Product	Time (h)	Conversion (%)[d]
1	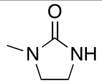	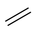 (1 bar)	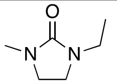	18	>99
2	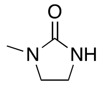	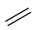 (1 bar)	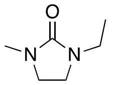	18	>99[b]
3	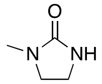	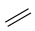 (1 bar)	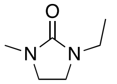	18	>99[c]
4	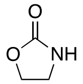	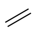 (1 bar)	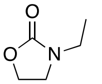	18	10
5	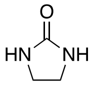	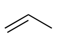 (6 bar)	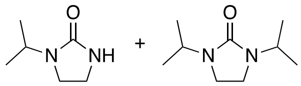	18	>99 (1:3)
6	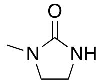	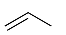 (6 bar)	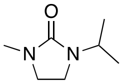	18	>99
7	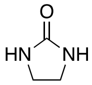	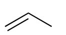 (4 bar)	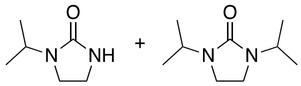	18	76 (2:1)
8	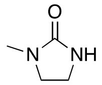	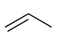 (4 bar)	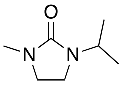	18	>99
9	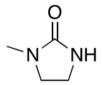	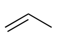 (3 bar)	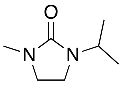	18	>99
10	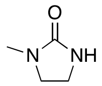	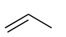 (2 bar)	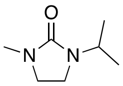	18	87
11	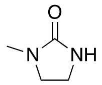	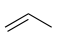 (1 bar)	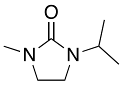	18	59

12	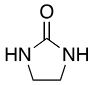	1-octene (15 equiv)	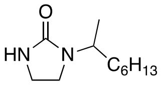	66	66[c]
13	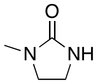	1-octene (15 equiv)	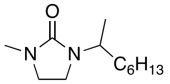	66	60
14	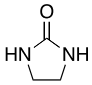	cyclopentene (15 equiv)	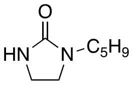	66	<5
15	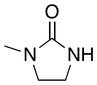	cyclopentene (15 equiv)	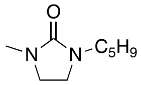	66	19
16	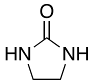	cyclohexene (15 equiv)	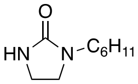	66	<5
17	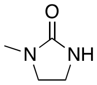	cyclohexene (15 equiv)	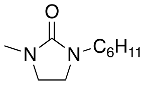	66	30
18	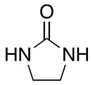	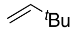 (15 equiv)	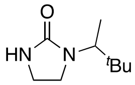	66	-
19	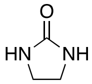	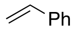 (15 equiv)	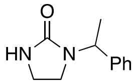	66	-

[a]Reaction was performed with nucleophile (0.20 mmol), 1-alkene, gold catalyst (0.01 mmol) and $AgSbF_6$ (0.01 mmol) as chloride abstractor in 1,4-dioxane (1 mL) at 100 °C. [b]at 80 ºC.[c]at 60 ºC. [d]Conversion was determined by ^{1}H NMR spectroscopy with anisole as the internal standard.

5. Kinetic experiments.

Kinetics of the hydroamination of 1-methyl-imdazolidine-2-one with ethylene at 6 bar.

Complex **13** at the indicated concentration and 1-methyl-imdazolidine-2-one (6 mg, 0.06 mmol) were dissolved in $CDCl_3$ (0.2 mL) in a high-pressure NMR tube. The tube was freeze-pumped to remove the nitrogen gas, filled with 6 bar of ethylene. The reaction was monitored by ^{1}H NMR spectroscopy at 100 °C at different times. As stated in the main text, the reaction follows a second order dependence on the amide as evinced by the representation of the corresponding integrated rate law (Figure 5 in the main text). For comparison, zero and first order representations are depiteced in Figures S66 and S67, respectively. The experiment shown in Figure 5 in the main text was also carried out in a sealed pressure vial taking alicuots for NMR analysis and resulting in a similar kinetic profile. Thus, for experimental convenience, all other kinetic experiments were run in high-pressure NMR tubes. Similarly, we performed the same experiment in the high-pressure NMR tube with intermittent shaking every five minutes, obtaining an almost identical kinetic profile, in agreement with excess ethylene in solution along the reaction coordinate and with the the zero-order dependence of the reaction rate on its concentration.

The activation energy (ΔG) of the process was calculated at 373.15 K (T) from the Arrenhius equation:

$$k = \frac{k_B \cdot T}{h} e^{-\Delta G / RT}$$

where k is the kinetic constant obtained from the second-order kinetic representation (k = 1.43 x 10^{-3} s^{-1}), k_B is the Boltzmann constant (1.38 x 10^{-23} J·K^{-1}), h is the Planck constant (6.63 x 10^{-34} J·s) and R is the gas constant (8.314 J·K^{-1}·mol^{-1}) giving $\Delta G_{373\ K}$ = **26.8 kcal/mol** as the free energy of activation.

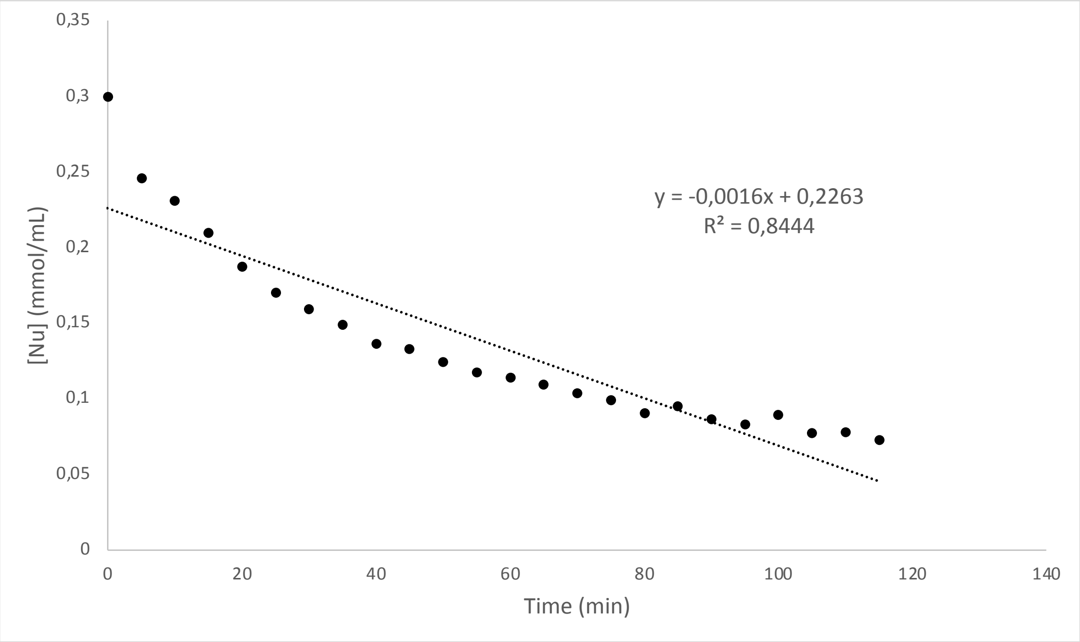

Figure S67. Zero-order kinetic representation of the consumption of 1-methyl-imdazolidin-2-one at 100 °C in $CDCl_3$ under 6 bar of ethylene pressure.

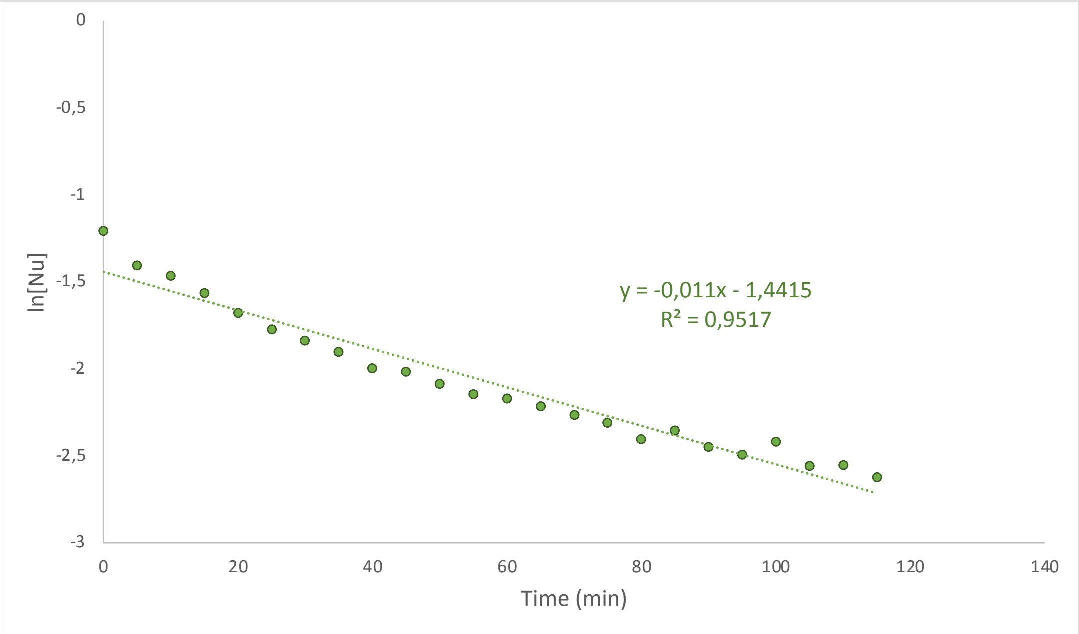

Figure S68. First-order kinetic representation of the consumption of 1-methyl-imdazolidin-2-one at 100 °C in $CDCl_3$ under 6 bar of ethylene pressure.

Table S3. Pseudo-second-order rate constants for the hydroamination of 1-methyl-imdazolidine-2-one **9’** (0.3 M) with ethylene (6 bar) catalyzed by gold complex **13** in $CDCl_3$ at 100 °C.

Entry	[Au] = [**13**] (M)	10^3 k_{obs} (M^{-1} s^{-1})
1	0.0055	0.19 ± 0.01
2	0.011	0.36 ± 0.01
3	0.022	0.86 ± 0.03
4	0.030	1.43 ± 0.03
5	0.045	1.89 ± 0.1

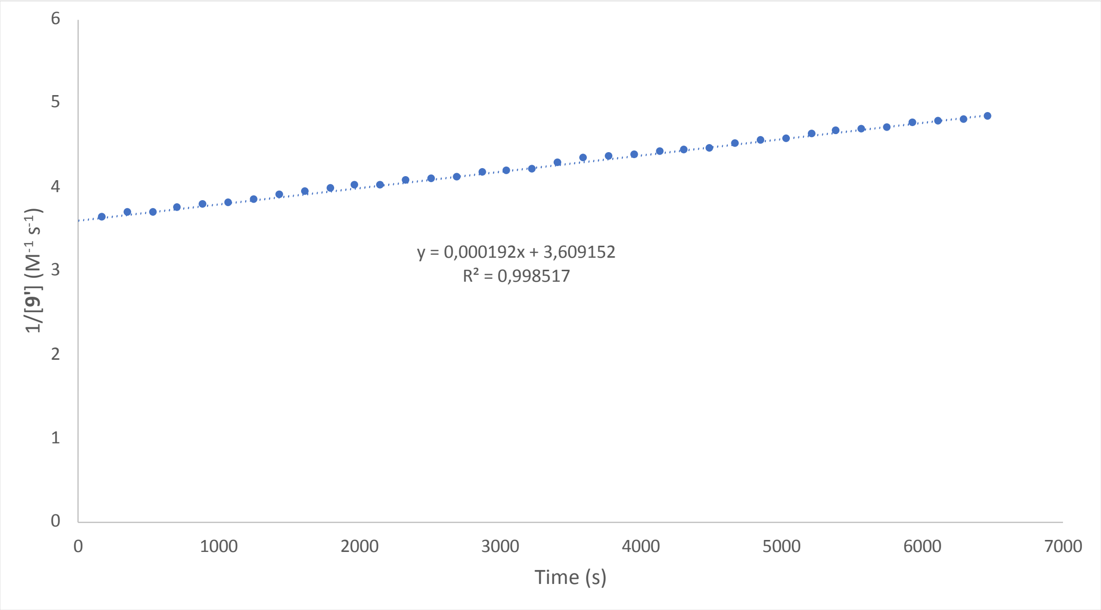

Figure S69. Plot of 1/[**9’**] versus time for the hydroamination of 1-methyl-imdazolidin-2-one 9’ (6 mg, 0.06 mmol, 0.055 M) with ethylene 6 bar caatlyzed by complex **13** (2 mol%, 0.0055 M).

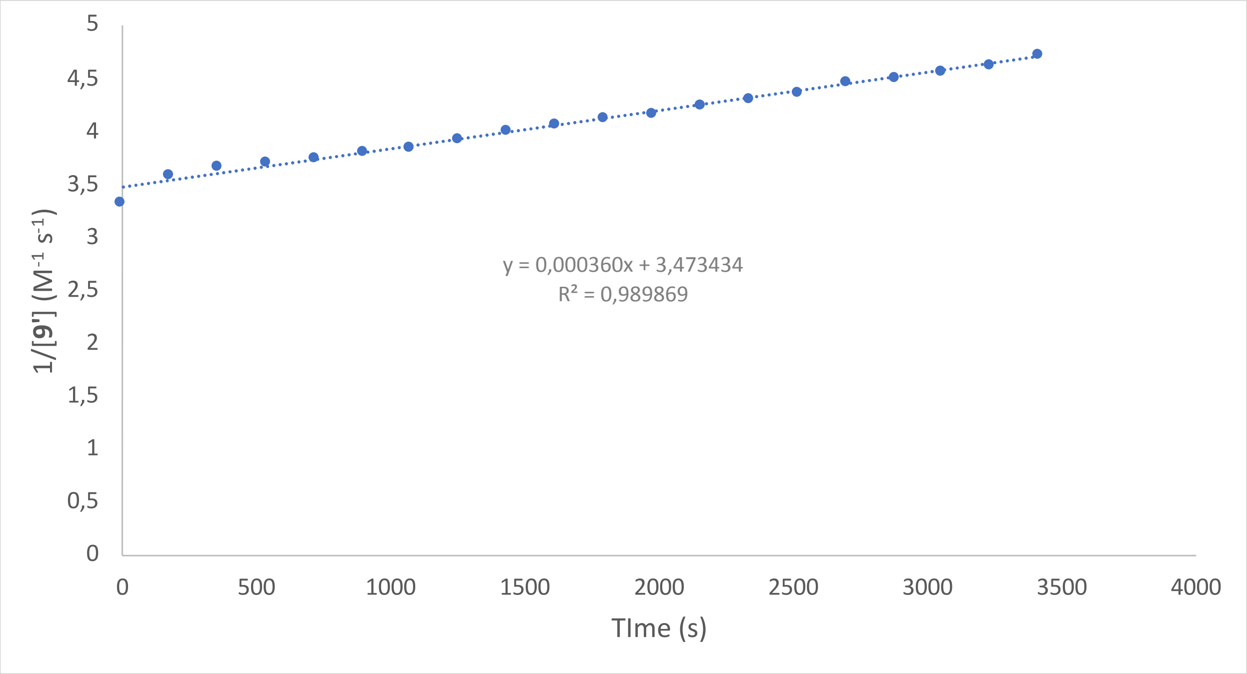

Figure S70. Plot of 1/[**9'**] versus time for the hydroamination of 1-methyl-imdazolidin-2-one 9' (6 mg, 0.06 mmol, 0.055 M) with ethylene 6 bar caatlyzed by complex **13** (4 mol%, 0.011 M).

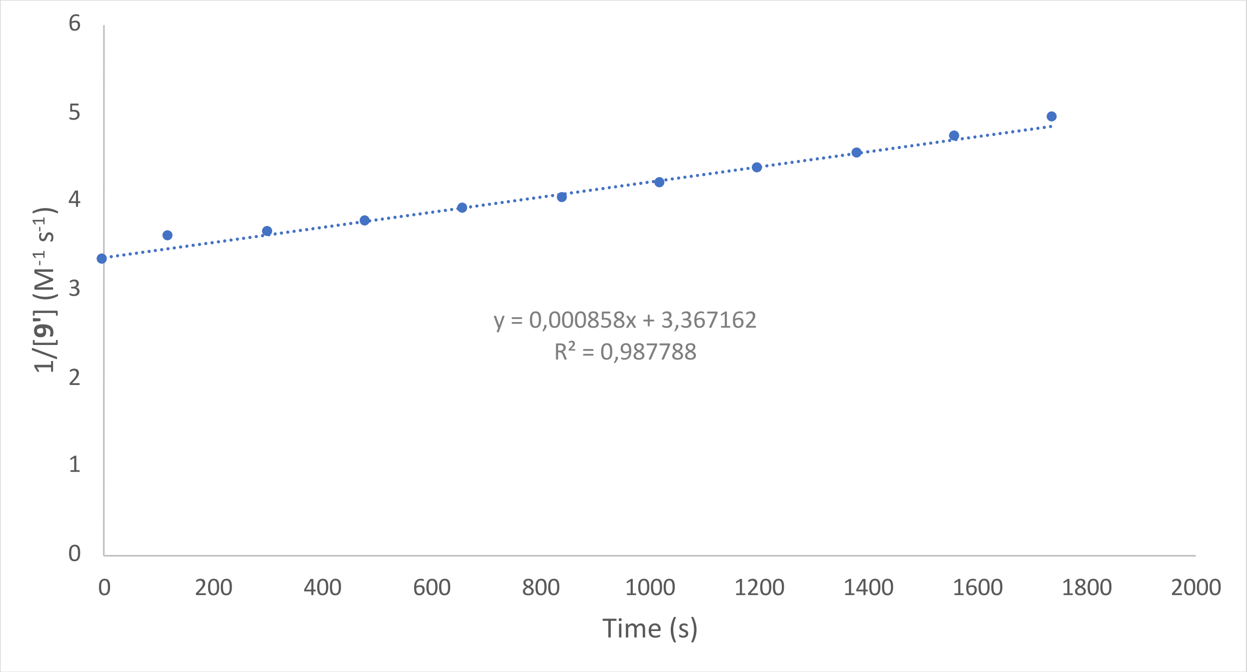

Figure S71. Plot of 1/[**9'**] versus time for the hydroamination of 1-methyl-imdazolidin-2-one 9' (6 mg, 0.06 mmol, 0.055 M) with ethylene 6 bar caatlyzed by complex **13** (8 mol%, 0.022 M).

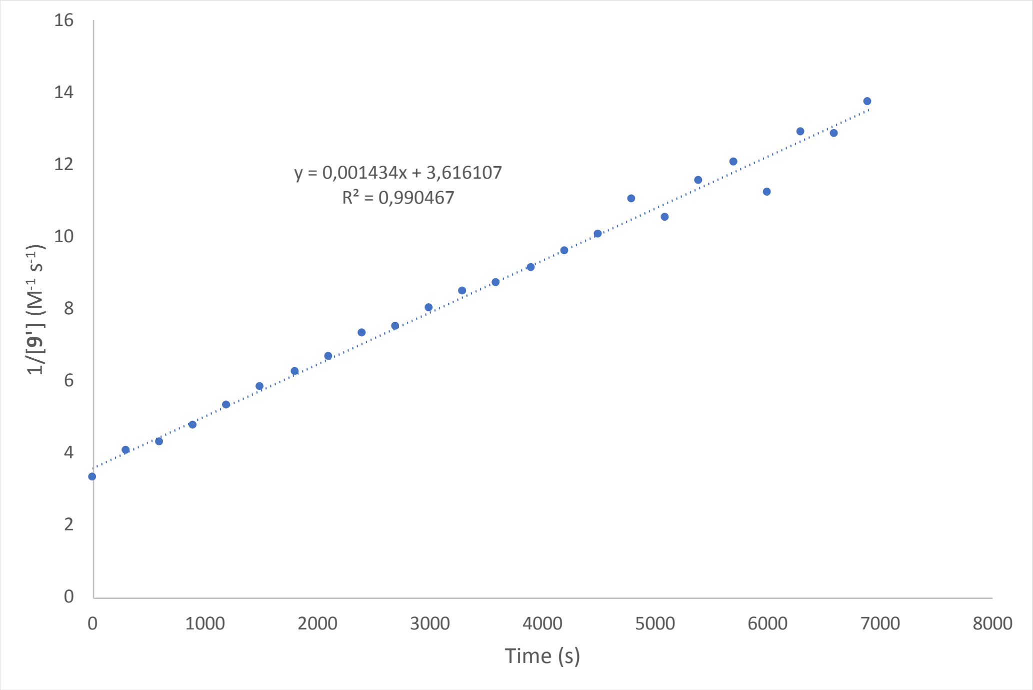

Figure S72. Plot of 1/[**9'**] versus time for the hydroamination of 1-methyl-imdazolidin-2-one 9' (6 mg, 0.06 mmol, 0.3 M) with ethylene 6 bar caatlyzed by complex **13** (10 mol%, 0,030 M).

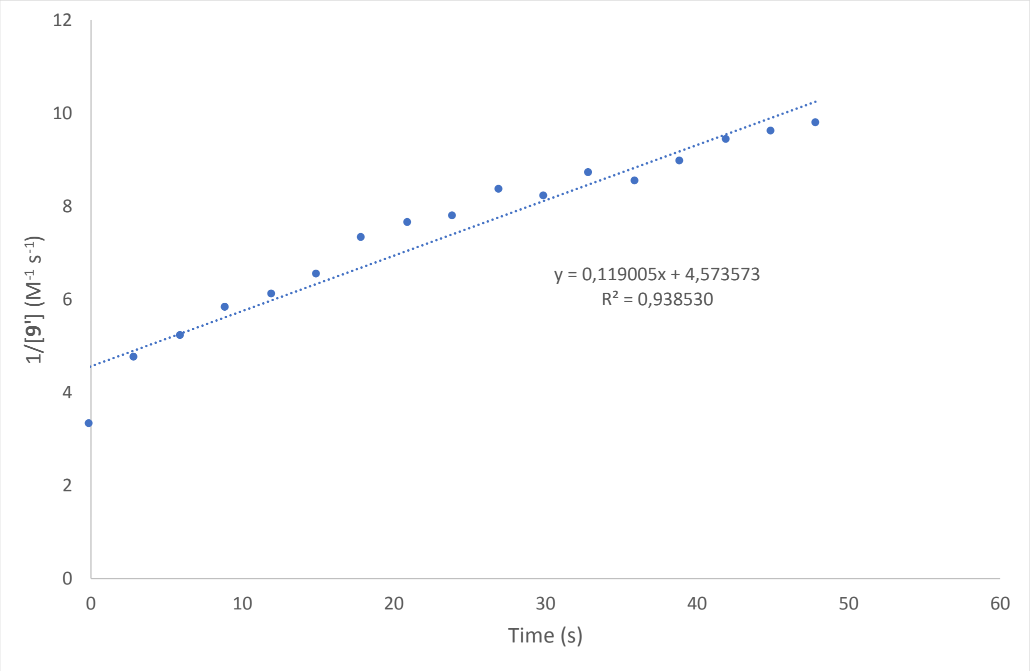

Figure S73. Plot of 1/[**9'**] versus time for the hydroamination of 1-methyl-imdazolidin-2-one 9' (6 mg, 0.06 mmol, 0.3 M) with ethylene 6 bar caatlyzed by complex **13** (15 mol%, 0,045 M).

Kinetics of the hydroamination of 1-methyl-imdazolidine-2-one with ethylene at different pressures.

Complex **13** (0.03 M) and 1-methyl-imdazolidine-2-one **9'** (0.2 M) were dissolved in $CDCl_3$ (0.2 mL) in a high-pressure NMR tube. The tube was freeze-pumped to remove the nitrogen gas, filled with the indicated ethylene pressure. The tube was placed in an oil bath at 60 °C and the reaction was monitored by ^{1}H NMR spectroscopy at different times.

Table S4. Pseudo-second-order rate constants for the hydroamination of 1-methyl-imdazolidine-2-one **9'** (0.3 M) with ethylene (6 bar) catalyzed by gold complex **13** in $CDCl_3$ at 100 °C.

Entry	Ethylene P (bar)	10^4 k_{obs} (M^{-1} S^{-1})
1	5	3.94 ± 0.1
2	6	3.85 ± 0.2
3	7	3.99 ± 0.2
4	8	3.95 ± 0.3

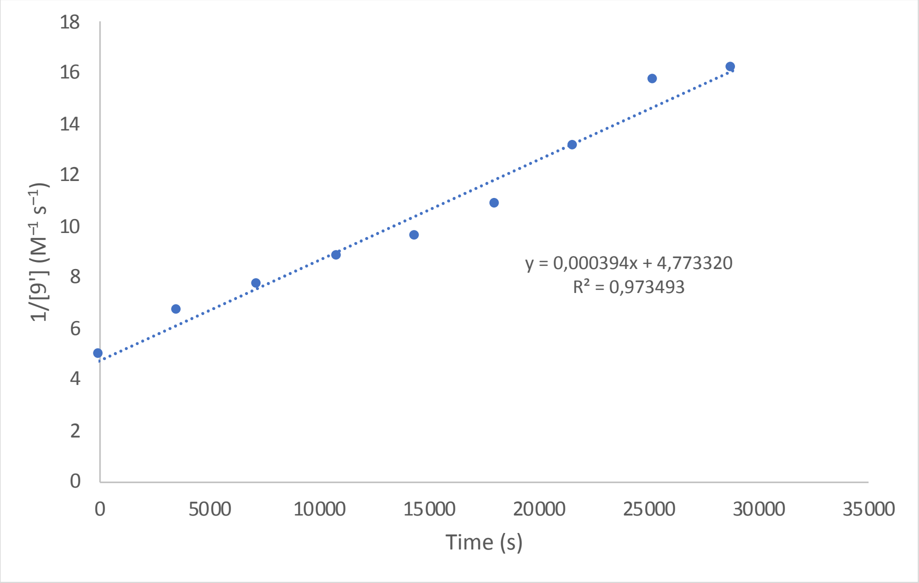

Figure S74. Plot of 1/[**9'**] versus time for the hydroamination of 1-methyl-imdazolidine-2-one **9'** (4 mg, 0.04 mmol, 0.2 M) with ethylene (5 bar) catalyzed by complex **13** (15 mol%, 0.03 M).

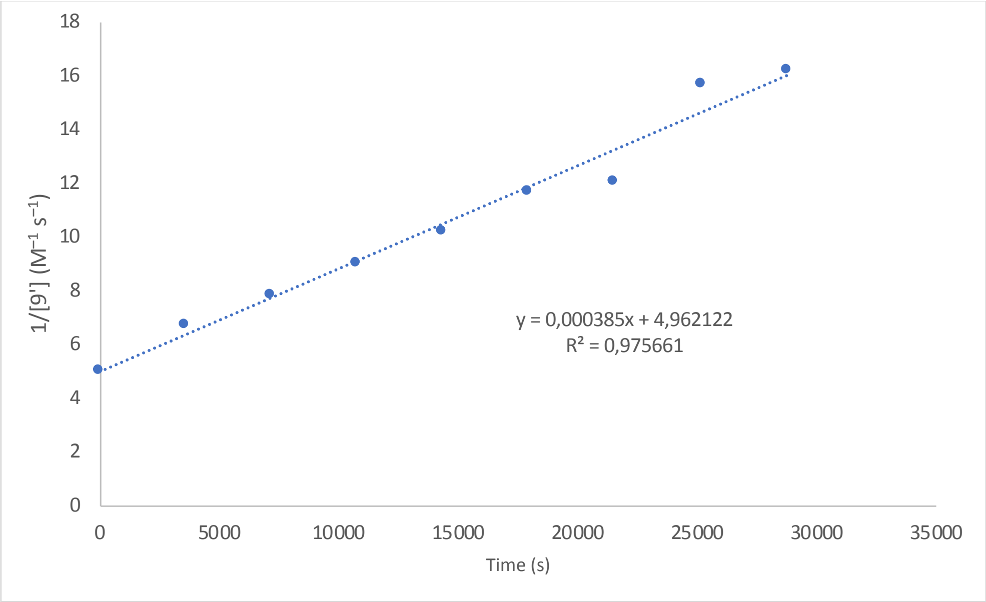

Figure S75. Plot of 1/[**9’**] versus time for the hydroamination of 1-methyl-imdazolidine-2-one **9’** (4 mg, 0.04 mmol, 0.2 M) with ethylene (6 bar) catalyzed by complex **13** (15 mol%, 0.03 M).

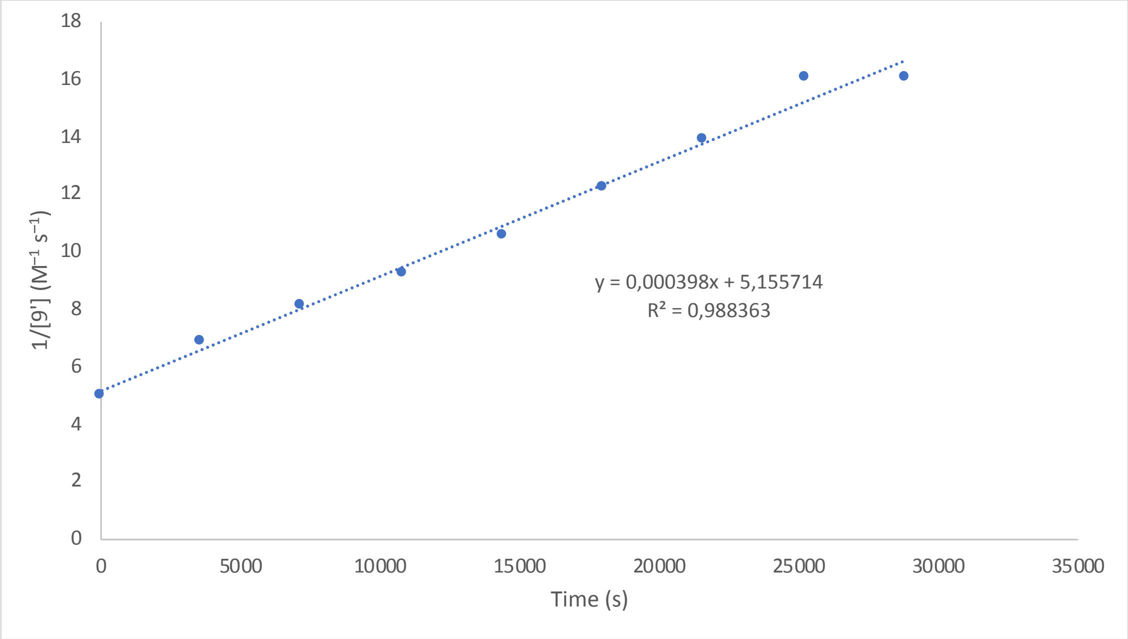

Figure S76. Plot of 1/[**9’**] versus time for the hydroamination of 1-methyl-imdazolidine-2-one **9’** (4 mg, 0.04 mmol, 0.2 M) with ethylene (7 bar) catalyzed by complex **13** (15 mol%, 0.03 M).

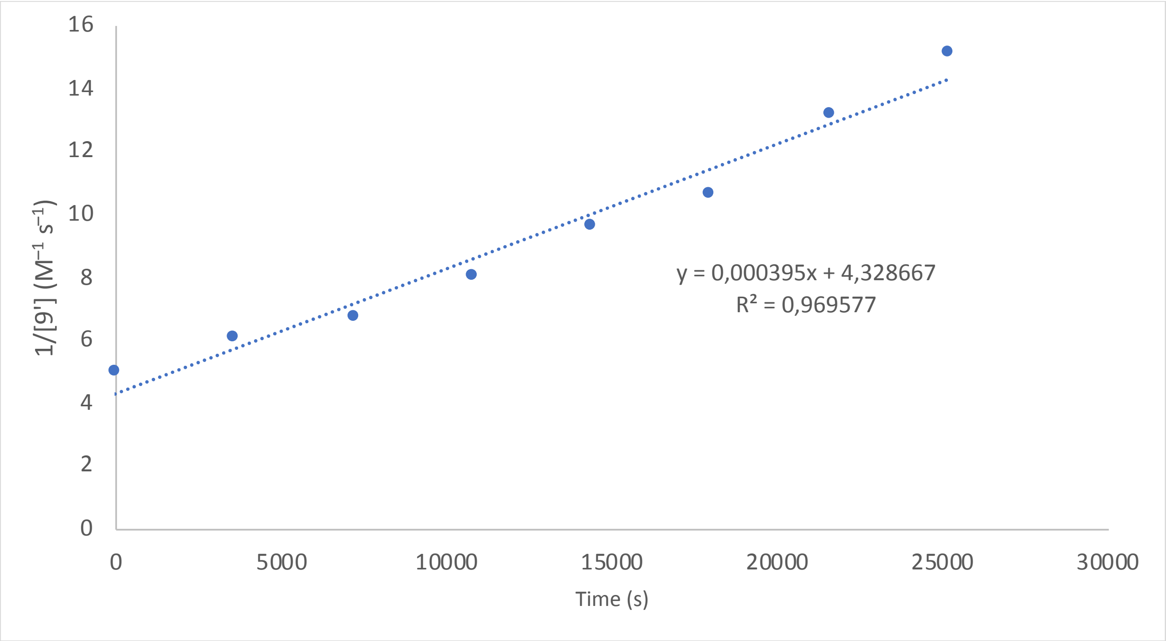

Figure S77. Plot of 1/[**9’**] versus time for the hydroamination of 1-methyl-imdazolidine-2-one **9’** (4 mg, 0.04 mmol, 0.2 M) with ethylene (8 bar) catalyzed by complex **13** (15 mol%, 0.03 M).

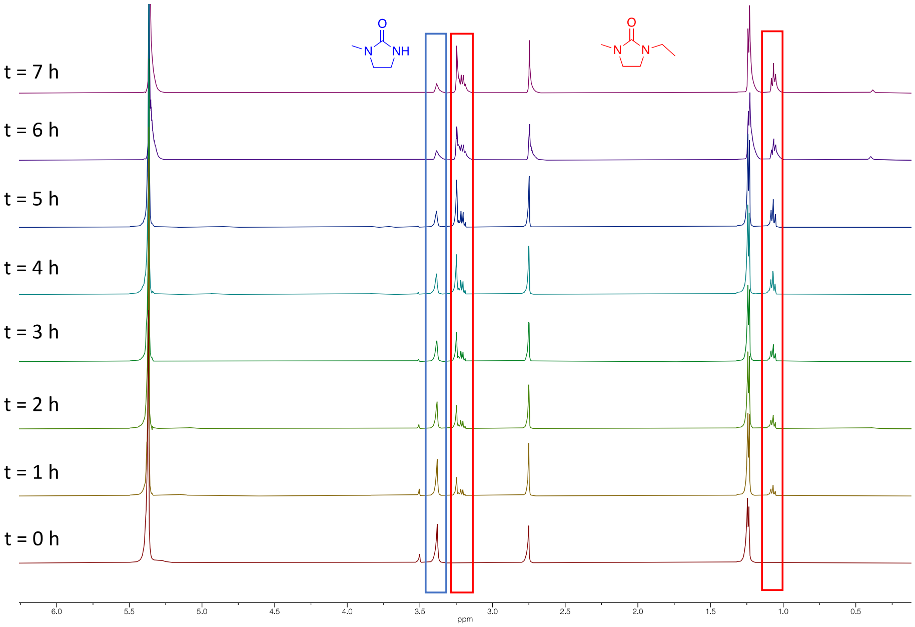

Figure S78. Example of ^{1}H NMR monitoring of the catalytic hydroamination of ethylene by 1-methyl-imdazolidine-2-one by complex **13**. The proton signals of 1-methyl-imdazolidin-2-one are marked in a blue rectangle and the proton signals of the into 1-methyl-2-ethylimidazolidine-2-one product are marked in red rectangles.

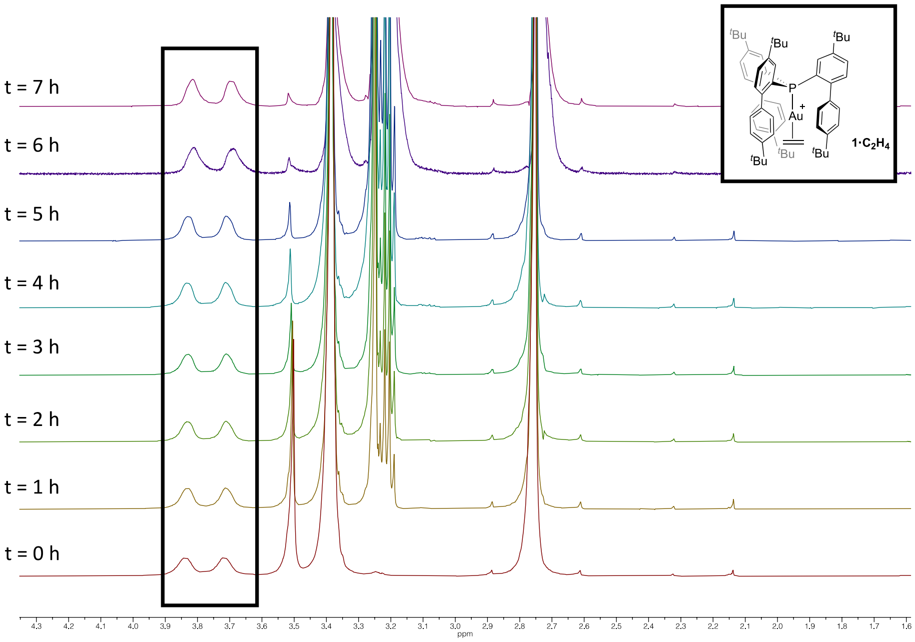

Figure S79. ^{1}H NMR monitoring of the catalytic hydroamination of ethylene by 1-methyl-imdazolidine-2-one by complex **13**. Selected region in which the proton signals of the coordinated ethylene in gold(I)-ethylene complex **1·C$_2$H$_4$** (marked in a black rectangle) are detected.

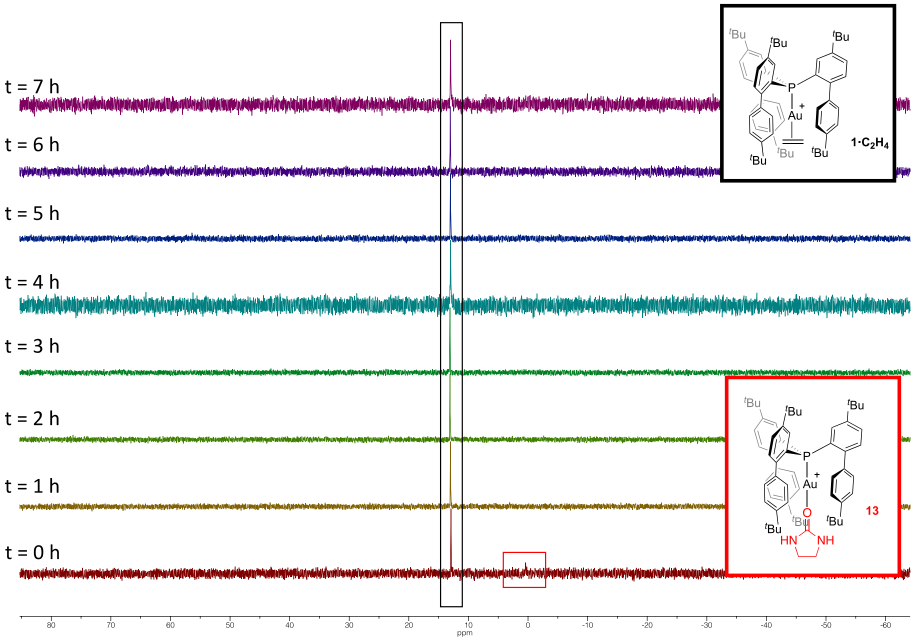

Figure S80. ^{31}P{^{1}H} NMR monitoring of the catalytic hydroamination of ethylene by 1-methyl-imdazolidine-2-one by complex **13**.

Kinetic isotopic effect (KIE)

Kinetic studies were carried out to determine the kinetic isotipic effect (KIE) of the gold-catalyzed hydroamination of ethylene by 1-methyl-imdazolidine-2-one (**9'**) and deuterated 1-methyl-imdazolidine-2-one (**9'**-d_1). Reaction progress was monitored by ^{1}H NMR spectroscopy.

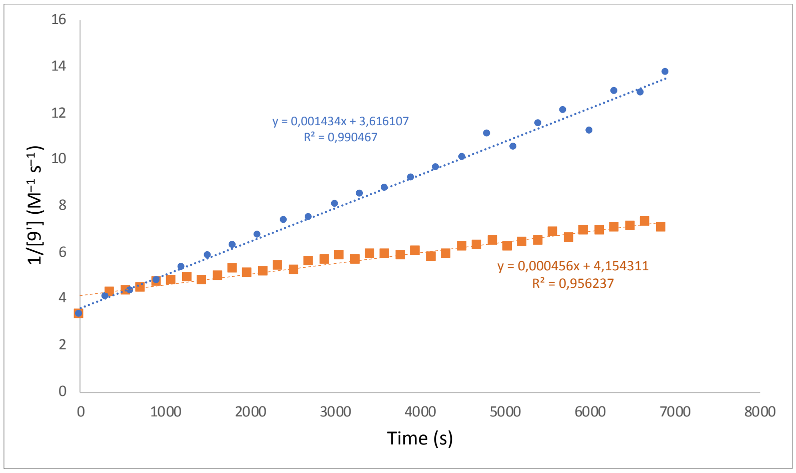

Figure S81. Plot of 1/[**9'**] versus time for the hydroamination and deuteroamination of ethylene (6 bar) with **9'** (blue) and **9'**-d_1 (orange) catalyzed by complex **13** (0.03 M), resulting in $k_H/k_D = 3.14$.

Derivation of differential rate equation for the hydroamination

The mechanism depicted for **Path C** (Figure 4 in the main manuscript) involving the intermolecular protodeauration is represented in a simplified form in Scheme S1. Key assumptions are made in the derivation of the rate equation: (a) prodeauration is irreversible, (b) gold(I)-ethylene complex **A** is the only complex that accumulates under catalytic conditions and (c) the addition of ethylene to regenerate complex **A** from **C** is neglected.

The reaction rate is defined by equation S1. Steady-state treatment of the intermediates [**B**] and [**C**] are depicted in eq S2 and S3. Rearrangement of eq S3 gives eq S4 which defines [**B**] in terms of [**C**]. Inserting the value of [**B**] in eq 2 gives eq S5 and S6, defining [**C**][**NuH**] in terms of [**A**] and [**Nu**]. Inserting eq S6 into the rate equation S1 gives the rate equation S7. Considering that the rate-limiting step is the protodeauration we could assyme that $k_3 <<< k_1$ and k_2 and, in that case, the final rate equation is eq S9, showing second order on nucleophile and first order in catalyst, as observed experimentally.

(eq S1) $\text{rate} = -d[C]/dt = k_3[C][NuH]$

(eq S2) $d[B]/dt = 0 = k_1[A][Nu] + k_{-2}[C][NuH] - k_{-1}[B] - k_2[B][Nu]$

(eq S3) $$d[C]/dt = 0 = k_2[B][Nu] - k_{-2}[C][NuH] - k_3[C][NuH]$$

(eq S4) $$[B] = \frac{k_{-2}[C][NuH] + k_3[C][NuH]}{k_2[Nu]}$$

(eq S5) $$k_1k_2[A][Nu]^2 - k_{-1}\, k_{-2}[C][NuH] - k_{-1}\, k_3[C][NuH] - k_2\, k_3[C][NuH][Nu] = 0$$

(eq S6) $$[C][NuH] = \frac{k_1k_2[A][Nu]^2}{k_{-1}k_{-2} + k_{-1}k_3 + k_2k_3[Nu]}$$

(eq S7) $$\text{rate} = \frac{k_1k_2k_3[A][Nu]^2}{k_{-1}k_{-2} + k_{-1}k_3 + k_2k_3[Nu]}$$

(eq S8) $$\text{rate} = \frac{k_1k_2k_3[A][Nu]^2}{k_{-1}k_{-2}} \qquad \text{when } k_3 <<< k_1 \text{ and } k_2$$

(eq S9) $$\text{rate} = K_1K_2k_3[A][Nu]^2$$

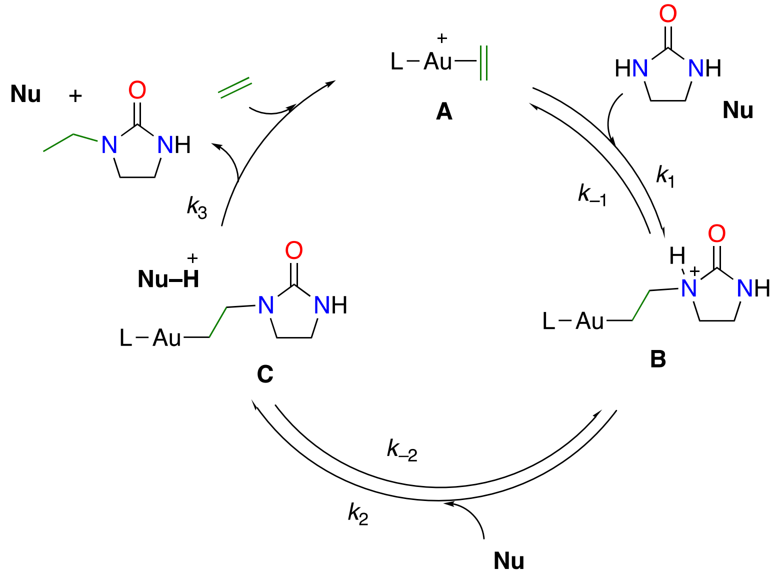

Scheme S1. Simplified mechanism of Path C for the gold(I)-caatlyzed hydroamination reaction.

The mechanism depicted for the alternative **Path B'** (Figure S93) involving the intramolecular protodeauration is represented in a simplified form in Scheme S2. Key assumptions are made in the derivation of the rate equation: (a) prodeauration is irreversible, (b) gold(I)-ethylene complex **A** is the only complex that accumulates under catalytic conditions and (c) the addition of ethylene to regenerate complex **A** from **C** is neglected.

The reaction rate is defined by equation S10. Steady-state treatment of the intermediate [**B**] and [**C**] are depicted in eq S11and S12. Rearrangement of eq S12 gives eq S13 which defines [**B**] in terms of [**C**]. Inserting the value of [**B**] in eq 11 gives eq S14 and S15, defining [**C**] in terms of [**A**] and [**Nu**]. Inserting

eq S15 into the rate equation S10 gives the rate equation S16. This equation does not comply with the determined second-order dependence on the nucleophile. Moreover, if we make the same consideration as above that $k_3 <<< k_1$ and k_2, then the final rate equation is eq S18, showing first order both in nucleophile and catalyst, which again does not fit with our experimental observations. Thus, even if this path seems to be slightly favored by computational studies, we postulate that it is the mechanism represented in Figure 4 the one taking place in our system, since that is consistent with our experimental results both for the order of nucleophile and the overall kinetic barrier.

(eq S10) $\text{rate} = -d[C]/dt = k_3[C]$

(eq S11) $d[B]/dt = 0 = k_1[A][Nu] + k_{-2}[C][Nu] - k_{-1}[B] - k_2[B][Nu]$

(eq S12) $d[C]/dt = 0 = k_2[B][Nu] - k_3[C] - k_{-2}[C][Nu]$

(eq S13) $[B] = \dfrac{k_3[C] + k_{-2}[C][Nu]}{k_2[Nu]}$

(eq S14) $k_1k_2[A][Nu]^2 - k_{-1}\,k_3[C] - k_{-1}\,k_{-2}[C][Nu] - k_2\,k_3[C][Nu] = 0$

(eq S15) $[C] = \dfrac{k_1k_2[A][Nu]^2}{k_{-1}k_3 + k_{-1}k_{-2}[Nu] + k_2k_3[Nu]}$

(eq S16) $\text{rate} = \dfrac{k_1k_2k_3[A][Nu]^2}{k_{-1}k_3 + k_{-1}k_{-2}[Nu] + k_2k_3[Nu]}$

(eq S17) $\text{rate} = \dfrac{k_1k_2k_3[A][Nu]^2}{k_{-1}k_{-2}[Nu]}$ when $k_3 <<< k_1$ and k_2

(eq S18) $\text{rate} = K_1K_2k_3[A][Nu]$

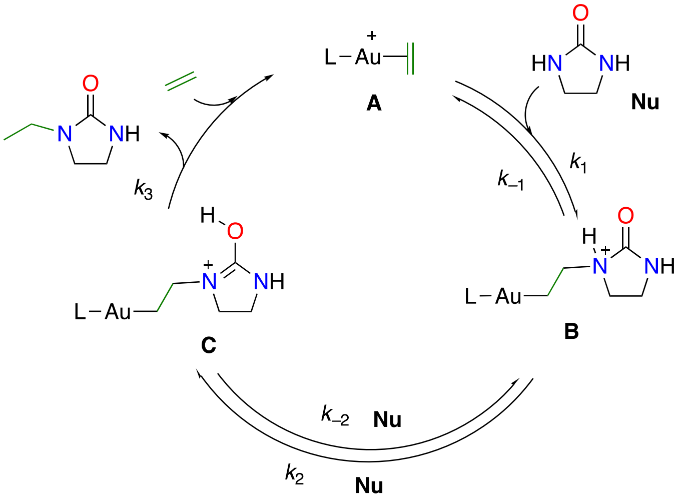

Scheme S2. Simplified mechanism of Path B' for the gold(I)-caatlyzed hydroamination reaction.

6. Crystal structure determinations

Crystallographic details. Low-temperature diffraction data were collected on a D8 Quest APEX-III single crystal diffractometer with a Photon III detector and a IµS 3.0 microfocus X-ray source (**7**, **8**, **3·C_2H_4, 8·C_2H_4, [6]□**, **12**, **13 and 14**) at the Instituto de Investigaciones Químicas, Sevilla. Data were collected by means of ω and φ scans using monochromatic radiation λ(Mo Kα1) = 0.71073 Å. The diffraction images collected were processed and scaled using APEX-III software. Structures **7** and **8** were solved with SHELXT. Using Olex2,[2] the structures of (**3·C_2H_4, 8·C_2H_4, [6]□**, **12**, **13 and 14**) were solved with olex2.solve 1.3.[3] All structures were refined against F^2 on all data by full-matrix least squares with SHELXL.[4] All non-hydrogen atoms were refined anisotropically. Hydrogen atoms were included in the model at geometrically calculated positions and refined using a riding model. The isotropic displacement parameters of all hydrogen atoms were fixed to 1.2 times the U value of the atoms to which they are linked (1.5 times for methyl groups). In four of the seven reported structures we used the program SQUEEZE to compensate for the contribution of disordered solvent molecules and counteranions, which account for 2 pentane (**[6]□**), 12 benzene (**7**), and 2 diclhoromethane molecules (**12**), 1 hexafluoroantimoniate (**13**) in the unit cell.

A summary of the fundamental crystal and refinement data are given in Tables S5-S7. Atomic coordinates, anisotropic displacement parameters and bond lengths and angles can be found in the cif files, which have been deposited in the Cambridge Crystallographic Data Centre with no. 2129167–2129172. These data can be obtained free of charge from The Cambridge Crystallographic Data Centre via www.ccdc.cam.ac.uk/data_request/cif.

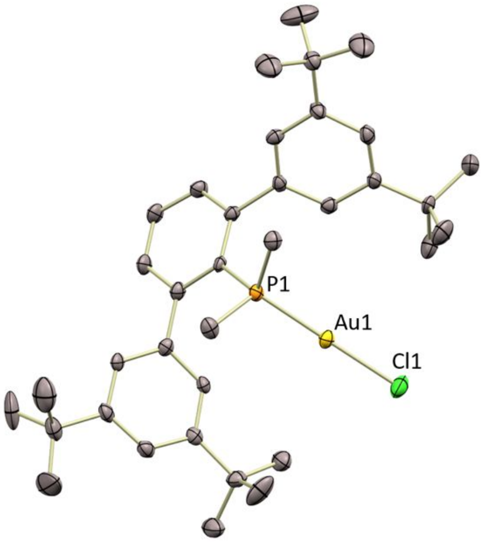

Figure S82. ORTEP diagram of complex **7**. Hydrogen atoms are excluded for clarity. Thermal ellipsoids are set at 50% probability.

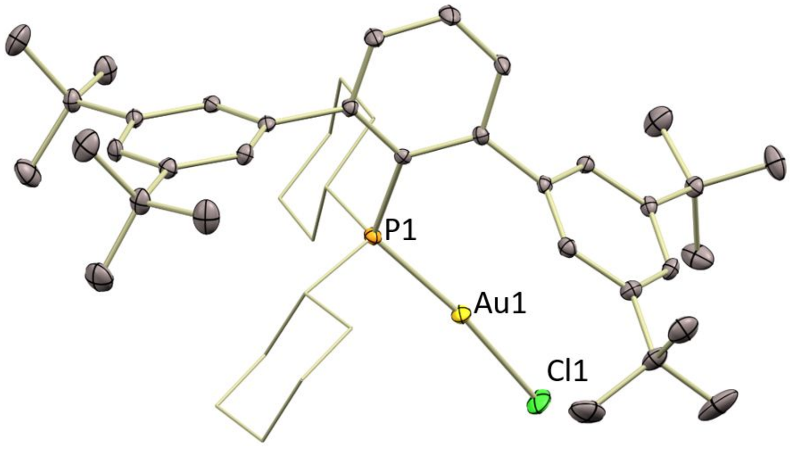

Figure S83. ORTEP diagram of complex **8**. Hydrogen atoms are excluded for clarity while the cyclohexyl groups are representated in wireframe format. Thermal ellipsoids are set at 50% probability.

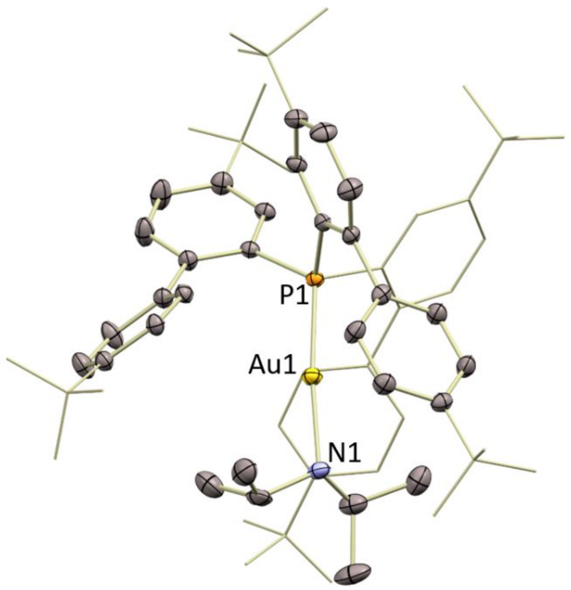

Figure S84. ORTEP diagram of complex **12**. Counteranion and hydrogen atoms are excluded for clarity, while *tert*-butyl groups and one biaryl fragment are representated in wireframe format. Thermal ellipsoids are set at 50% probability.

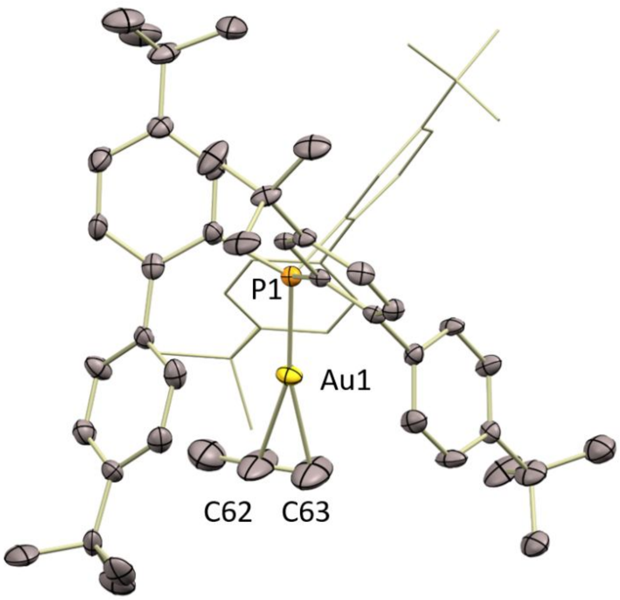

Figure S85. ORTEP diagram of complex **14**. Only one of the two independent molecules present in the asymmetric unir is shown. Counteranion, solvent molecule and hydrogen atoms are excluded for clarity, while *tert*-butyl groups and one biaryl fragment are representated in wireframe format. Thermal ellipsoids are set at 50% probability.

Table S5. Crystal data and structure refinement for compounds **7**, **8** and **3·C_2H_4**.

	**7**	**8**	**3·C_2H_4**
formula	$C_{120}H_{165}Au_3Cl_3P_3$	$C_{46}H_{67}AuClP$	$C_{31}H_{49}AuF_6PSb$
Fw	2397.67	883.38	885.39
cryst.size, mm	0.12 × 0.11 × 0.04	0.20 × 0.17 × 0.10	0.21 × 0.13 × 0.11
crystal system	Monoclinic	Orthorhombic	Triclinic
space group	*C2*/c	$P2_12_12_1$	*P-1*
a, Å	54.233 (8)	12.6694 (2)	8.7636(7)
b, Å	14.371 (2)	18.1472 (4)	13.6876(12)
c, Å	32.268 (5)	18.8940 (4)	28.412(2)
α, deg	90	90	86.489(3)
β, deg	102.216 (9)	90	89.678(3)
γ, deg	90	90	77.831(3)
V, Å^3	24579 (6)	4344 (15)	3325.1(5)
T, K	193	193	100
Z	8	4	4
ρ_{calc}, g cm^{-3}	1.296	1.351	1.769
μ, mm^{-1} (MoKα)	3.718	3.514	5.320
F(000)	9744	1816	1736
absorption corrections	multi-scan, 0.60– 0.74	multi-scan, 0.60 – 0.75	multi-scan, 0.44 – 0.75
θ range, deg	1.291–2525.999	2.156–30.543	2.030–26.000
no. of rflns measd	194519	48475	95123
R_{int}	0.1138	0.0297	0.1226
no. of rflns unique	24156	13243	13023
no. of params / restraints	1201 / 18	454 / 0	745 / 10
R_1 ($I > 2\sigma(I)$) [a]	0.0604	0.0217	0.0576
R_1 (all data)	0.1113	0.0267	0.0832
wR_2 ($I > 2\sigma(I)$)	0.1473	0.0393	0.1175
wR_2 (all data)	0.1790	0.0404	0.1311
Diff.Fourier.peaks min/max, eÅ^{-3}	-2.13 / 2.11	-0.47 / 0.85	-2.644/ 3.576
CCDC number	2129167	2129168	2129172

Table S6. Crystal data and structure refinement for compounds **$8\cdot C_2H_4$**, **[6]□** and **12**.

	**$8\cdot C_2H_4$**	**[6]□**	**12**
formula	$C_{48}H_{71}AuPF_6Sb$ + $2(CH_2Cl_2) + C_3H_7$	$C_{32}H_{39}AuF_6PSb$	$C_{66}H_{90}AuF_6NPSb$
Fw	1324.67	887.32	1361.07
cryst.size, mm	0.18 × 0.15 × 0.12	0.17 × 0.13 × 0.10	0.20 × 0.16 × 0.13
crystal system	Monoclinic	Monoclinic	Triclinic
space group	*C2/c*	*P2₁/n*	*P-1*
a, Å	33.253(3)	11.3949(8)	15.3509(9)
b, Å	13.3849(11)	16.7262(9)	15.4730(9)
c, Å	26.020(2)	18.5670(12)	19.0096(10)
α, deg	90	90	95.2651(17)
β, deg	93.079(3)	104.780(3)	103.1408(17)
γ, deg	90	90	117.9130(18)
V, Å^3	11564.5(17)	3421.7(4)	3781.0(4)
T, K	100	193	193
Z	8	4	2
ρ_{calc}, g cm^{-3}	1.522	1.722	1.196
μ, mm^{-1} (MoKα)	3.266	5.171	2.363
F(000)	5336	1720	1384
absorption corrections	multi-scan, 0.44 – 0.75	multi-scan, 0.57 – 0.75	multi-scan, 0.59 – 0.75
θ range, deg	1.938–25.999	2.213–26.000	1.993–26.745
no. of rflns measd	100015	65592	124627
R_{int}	0.0988	0.1169	0.0946
no. of rflns unique	11354	6718	16058
no. of params / restraints	647/ 67	374/7	739/ 0
R_1 ($I > 2\sigma(I)$) [a]	0.0503	0.0385	0.0366
R_1 (all data)	0.0686	0.0662	0.0574
wR_2 ($I > 2\sigma(I)$)	0.1252	0.0782	0.0815
wR_2 (all data)	0.1425	0.0921	0.0922
Diff.Fourier.peaks min/max, eÅ^{-3}	-2.479/2.185	-1.807/1.242	-2.140/1.471
CCDC number	2129171	2129170	2129169

Table S7. Crystal data and structure refinement for compounds **13** and **14**.

	**13**	**14**
formula	$C_{63}H_{81}AuF_3N_2OPSb_{0.5}$	$C_{63}H_{80}AuP \cdot C_{63}H_{79}AuP \cdot 2(F_6Sb)$
Fw	1228.10	2600.90
cryst.size, mm	0.15 × 0.14 × 0.10	0.17 × 0.07 × 0.05
crystal system	Triclinic	Monoclinic
space group	*P-1*	$P2_1/n$
a, Å	15.5190(7)	13.714 (5)
b, Å	15.6380(8)	51.58 (2)
c, Å	17.4595(10)	18.755 (9)
α, deg	68.369(2)	90
β, deg	80.530(2)	99.200 (18)
γ, deg	65.864(2)	90
V, $Å^3$	3594.0(3)	13096 (10)
T, K	193	193
Z	2	4
ρ_{calc}, g cm^{-3}	1.135	1.319
μ, mm^{-1} (MoKα)	2.363	2.73
F(000)	1255	5252
absorption corrections	multi-scan, 0.49 – 0.75	multi-scan, 0.53 – 0.75
θ range, deg	2.27 – 29.50	1.9 – 27.1
no. of rflns measd	240470	245736
R_{int}	0.0756	0.161
no. of rflns unique	20146	26045
no. of params / restraints	716 / 102	1410/ 198
R_1 ($I > 2\sigma(I)$) [a]	0.0575	0.1108
R_1 (all data)	0.0824	0.1321
wR_2 ($I > 2\sigma(I)$)	0.1475	0.2612
wR_2 (all data)	0.1680	0.2742
Diff.Fourier.peaks min/max, eÅ^{-3}	-2.43/1.29	-2.52/2.50
CCDC number	2152965	2152966

7. Buried volume analysis

The steric description of percent buried volume (%Vbur) has been shown to be a valid measure of the steric properties of monodentate ligands such as phosphines. Comparison of all phosphines used in this study is shown in Figure S86.[5]

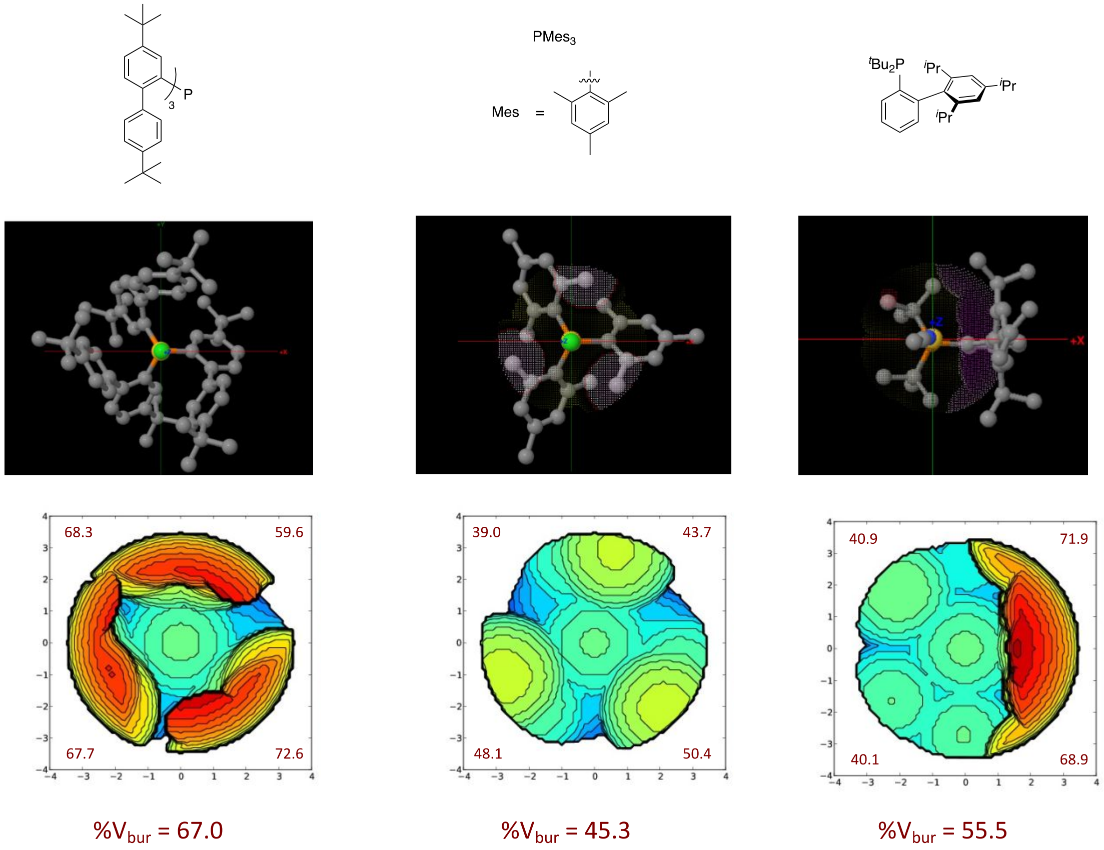

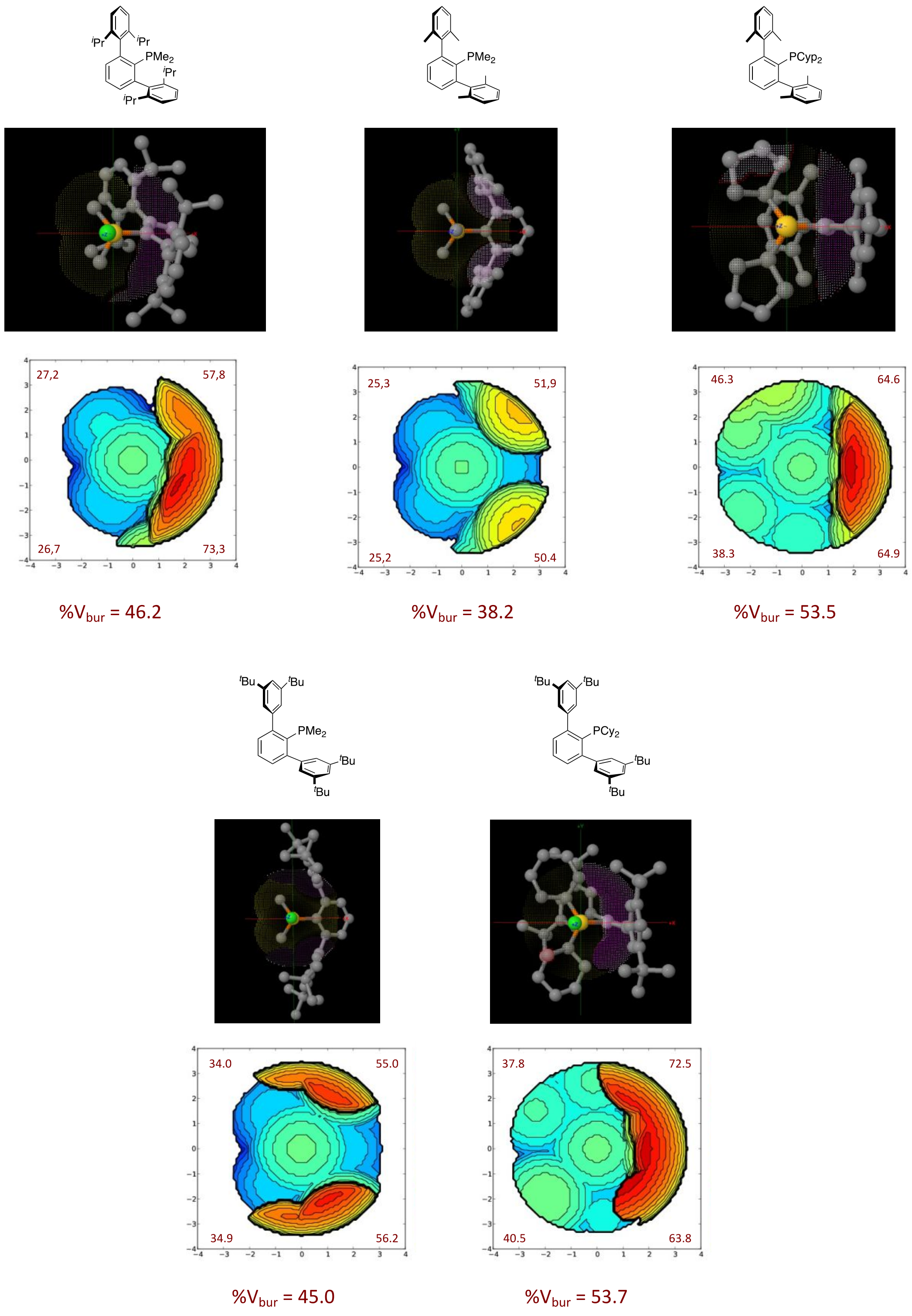

Figure S86. Schematic (a) and 3D representation (b) of the ligands, together with the corresponding steric maps (c) and calculated %Vbur for all the gold(I) chloride complexes in the study. The %Vbur of each quadrant are also indicated in red.

8. Computational details

Calculations were performed at the DFT level with the Gaussian 09 (Revision D.01) program.[6] The hybrid functional PBE0[7] was used throughout the computational study, and dispersion effects were accounted for by using Grimme's D3 parameter set with Becke−Johnson (BJ) damping at the optimization stage.[8] Geometry optimizations were carried out without geometry constraints, using the 6-31G(d,p)[9] basis set to represent the C, H, N, O and P atoms and the Stuttgart/Dresden Effective Core Potential and its associated basis set (SDD)[10] to describe the Au atoms. Bulk solvent effects (dichloromethane) were included at the optimization stage with the SMD continuum model[11]. The stationary points and their nature as minima or saddle points (TS) were characterized by vibrational analysis, which also produced enthalpy (H), entropy (S) and Gibbs energy (G) data at 298.15 K. The minima connected by a given transition state were determined by perturbing the transition states along the TS coordinate and optimizing to the nearest minimum. Free energies were corrected (ΔG_{qh}) to account for errors associated with the harmonic oscillator approximation, temperature (373.15 K) and concentration of all gold(I) species (c = 0.01 M) and imidazolidine-2-one (c = 0.2 M) with the Goodvibes code.[12] Thus, according to Truhlars's quasi-harmonic approximation for vibrational entropy, all vibrational frequencies below 100 cm^{-1} were set to this value.[13]

8.1 Hydroamination of ethylene: alternative mechanistic pathways

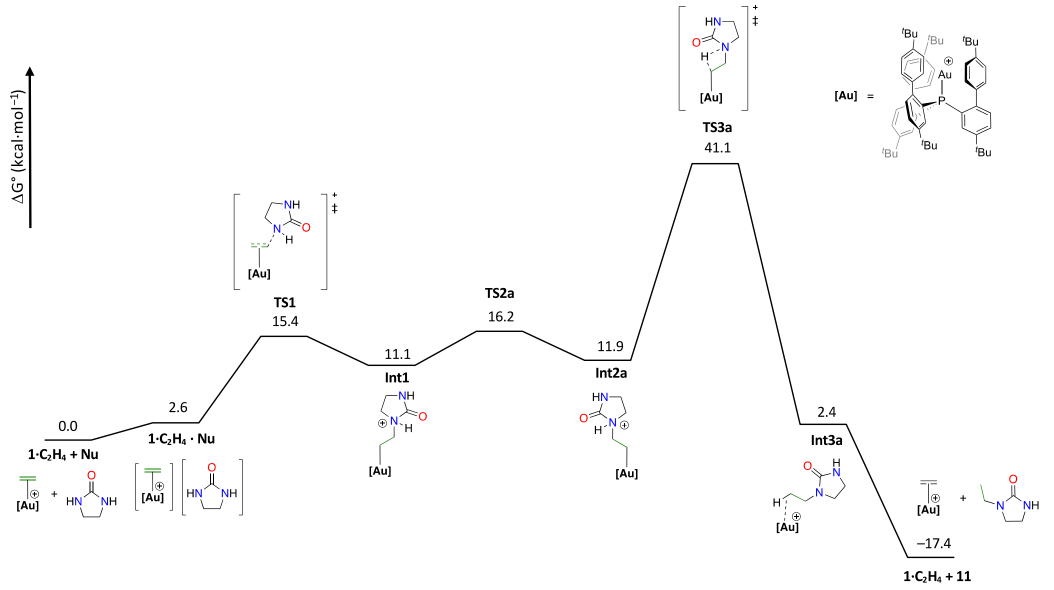

Figure S87. Free energy profile for the Au(I)-catalyzed hydroamination of ethylene with imidazolidine-2-one (path **A**).

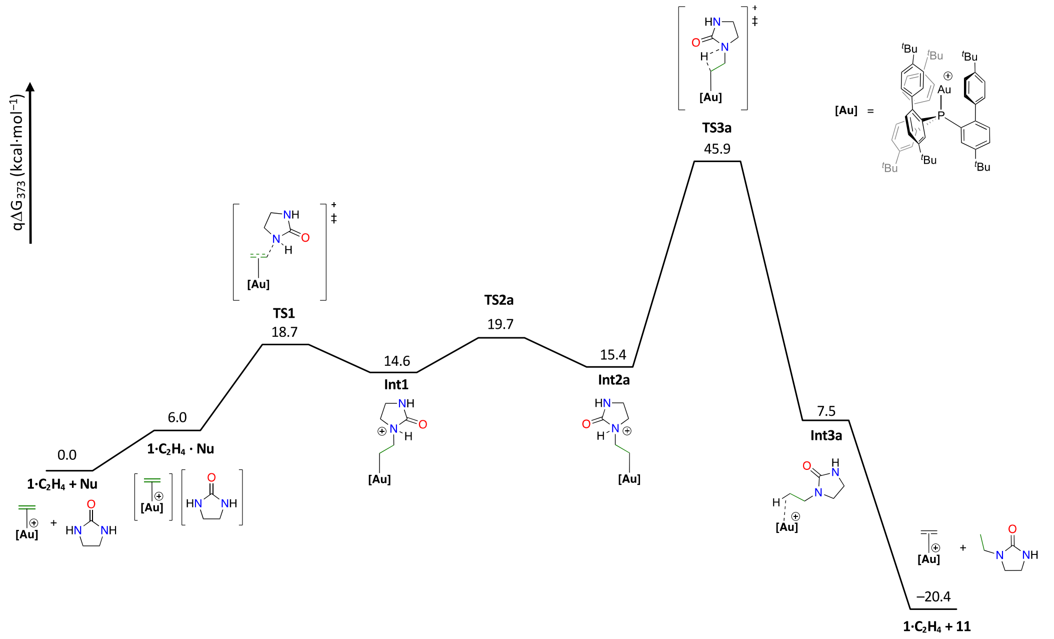

Figure S88. Corrected free energy (ΔG_{qh}) profile for the Au(I)-catalyzed hydroamination of ethylene with imidazolidine-2-one (path **A**).

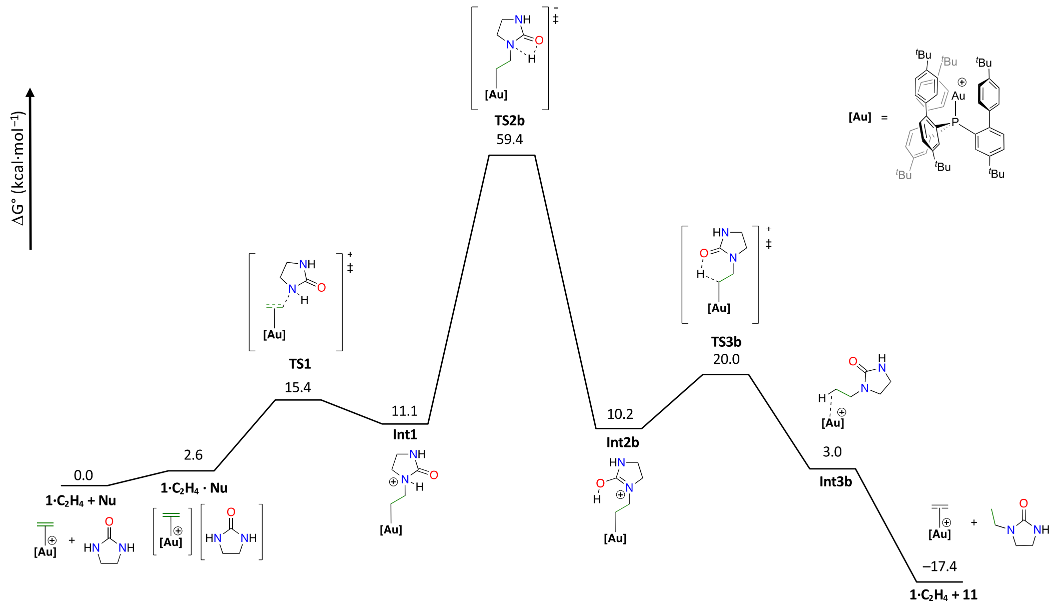

Figure S89. Free energy profile for the Au(I)-catalyzed hydroamination of ethylene with imidazolidine-2-one (path **B**).

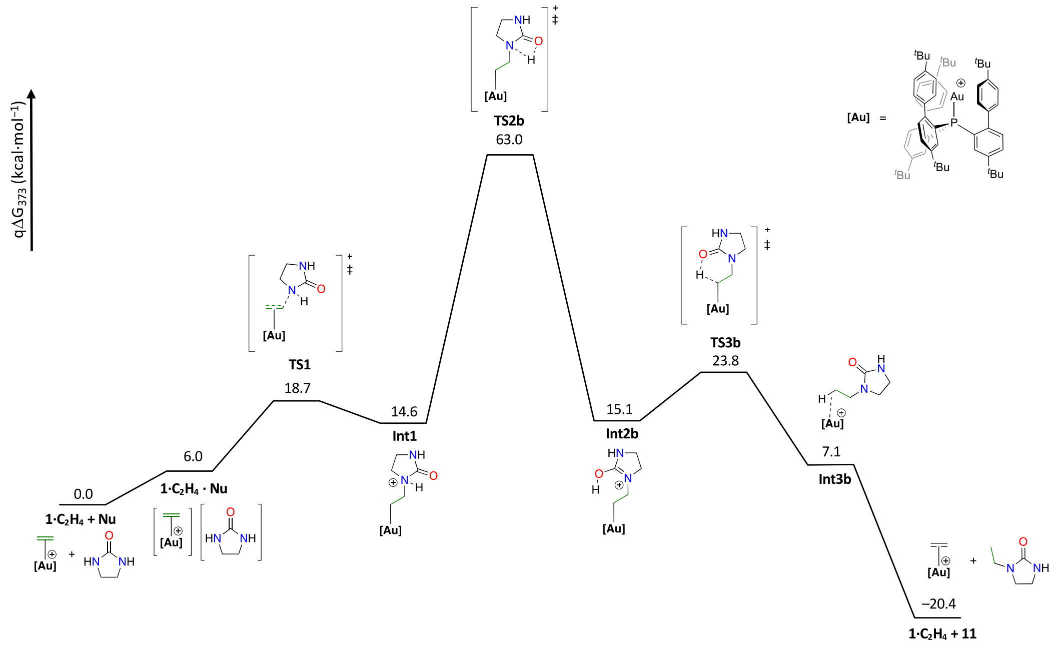

Figure S90. Corrected free energy (ΔG_{qh}) profile for the Au(I)-catalyzed hydroamination of ethylene with imidazolidine-2-one (path **B**).

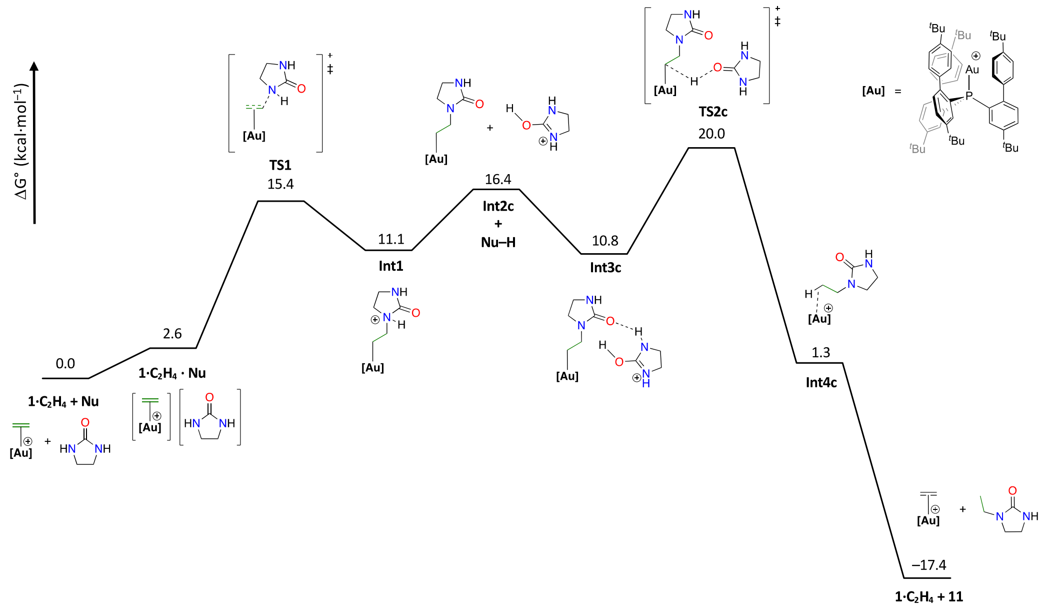

Figure S91. Free energy profile for the Au(I)-catalyzed hydroamination of ethylene with imidazolidine-2-one assisted by a second molecule of imidazolidine-2-one acting as a proton shuttle (path **C**).

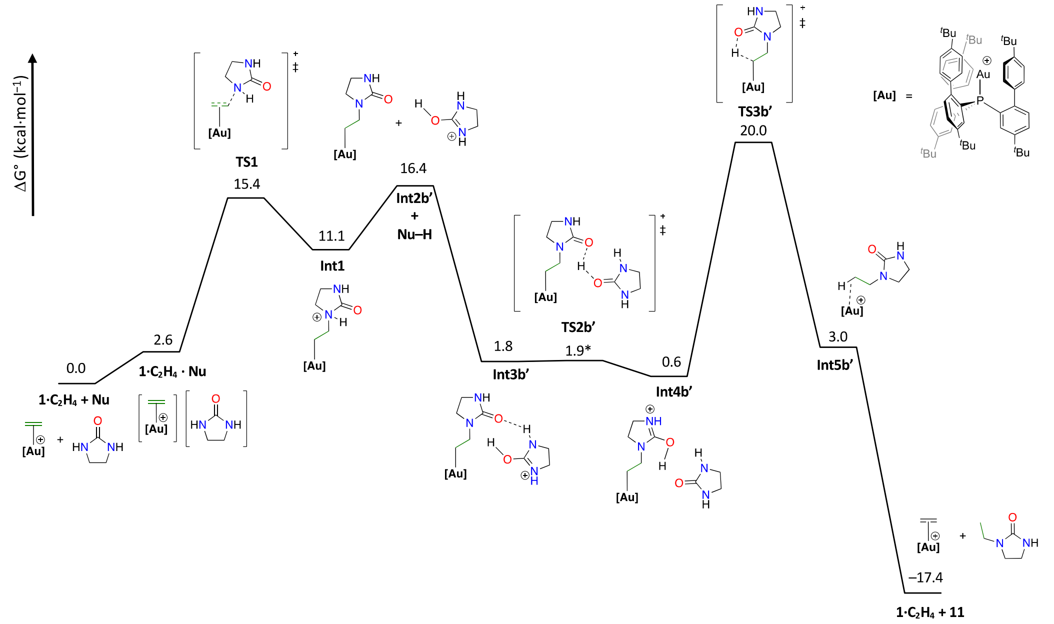

Figure S92. Free energy profile for the Au(I)-catalyzed hydroamination of ethylene with imidazolidine-2-one assisted by a second molecule of imidazolidine-2-one acting as a proton shuttle (path **B'**). *Due to an extremely flat surface, the energy difference relative to the preceding minimum was calculated according to electronic energies rather than free energies.

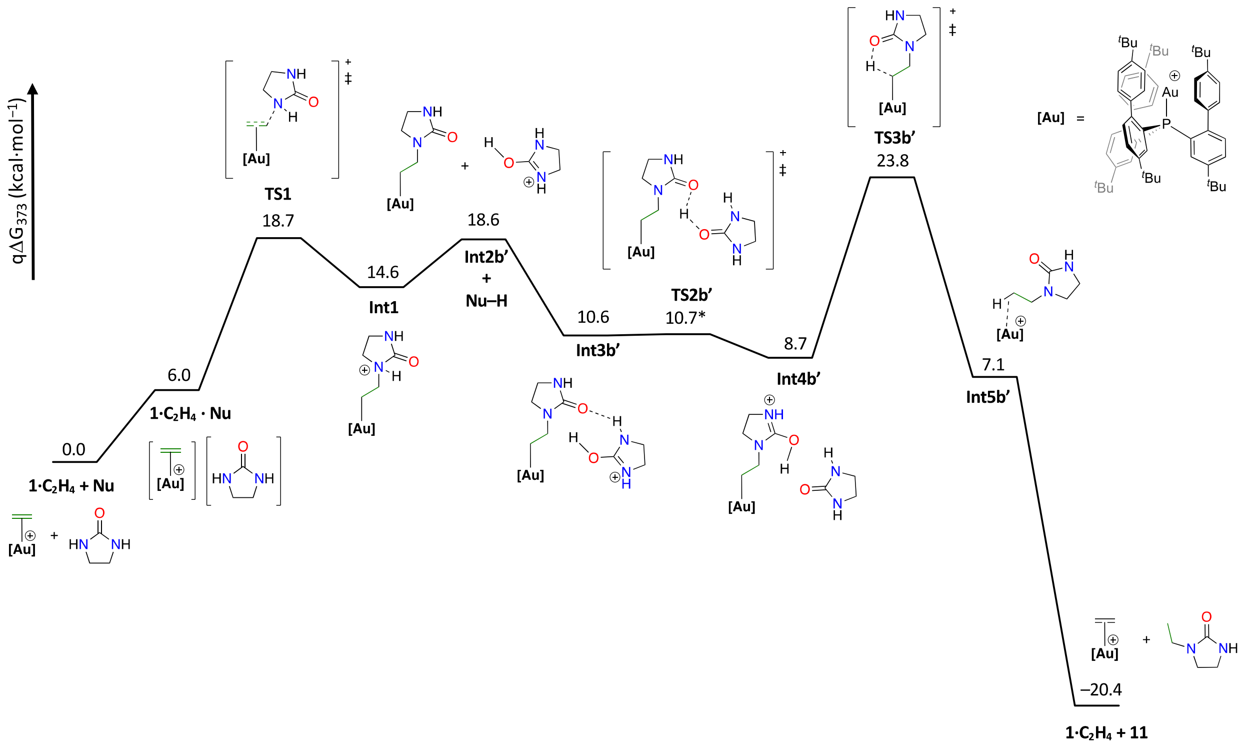

Figure S93. Corrected free energy (ΔG_{qh}) profile for the Au(I)-catalyzed hydroamination of ethylene with imidazolidine-2-one assisted by a second molecule of imidazolidine-2-one acting as a proton shuttle (path **B'**). *Due to an extremely flat surface, the energy difference relative to the preceding minimum was calculated according to electronic energies rather than free energies.

9. References

[1] Navarro, M.; Miranda-Pizarro, J.; Moreno, J. J. Navarro-Gilabart, C.; Fernández, I.; Campos J. A dicoordinate gold(I)–ethylene complex. *Chem. Commun.* **2021**, *57*, 9280-9283.

2 Dolomanov, O. V.; Bourhis, L. J.; Gildea, R. J.; Howard, J.A.K.; Puschmann, H. OLEX2: a complete structure solution, refinement and analysis program. *J. Appl. Cryst.* **2009**, *42*, 339-341.

[3] Bourhis, L. J.; Dolomanov, O. V.; Gildea, R. J.; Howard, J. A. K.; Puschmann, H. The anatomy of a comprehensive constrained, restrained refinement program for the modern computing environment - Olex2 dissected. *Acta Cryst.* **2015**, *A71*, 59-75.

[4] Sheldrick, G. M. A short history of SHELX. *Acta Cryst*. **2008,** *A64*, 112-122.

[5] Falivene, L.; Credendino, R.; Poater, A.; Petta, A.; Serra, L.; Oliva, R.; Scarano, V.; Cavallo. L. SambVca 2. A Web Tool for Analyzing Catalytic Pockets with Topographic Steric Maps. *Organometallics* **2016**, *35*, 2286-2293.

[6] Frisch, M. J.; Trucks, G. W.; Schlegel, H. B.; Scuseria, G. E.; Robb, M. A.; Cheeseman, J. R.; Scalmani, G.; Barone, V.; Mennucci, B; Petersson, G. A.; Nakatsuji, H.; Caricato, M.; Li, X.; Hratchian, H. P.; Izmaylov, A. F.; Bloino, J.; Zheng, G.; Sonnenberg, J. L.; Hada, M.; Ehara, M.; Toyota, K.; Fukuda, R.; Hasegawa, J.; Ishida, M.; Nakajima, T.; Honda, Y.; Kitao, O.; Nakai, H.; Vreven, T.; Montgomery, J. A. J.; Peralta, J. E.; Ogliaro, F.; Bearpark, M.; Heyd, J. J.; Brothers, E.; Kudin, K. N.; Staroverov, V. N.; Kobayashi, R.; Normand, J.; Raghavachari, K.; Rendell, A.; Burant, J. C.; Iyengar, S. S.; Tomasi, J.; Cossi, M.; Rega, N.; Millam, J. M.; Klene, M.; Knox, J. E.; Cross, J. B.; Bakken, V.; Adamo, C.; Jaramillo, J.; Gomperts, R.; Stratmann, R. E.; Yazyev, O.; Austin, A. J.; Cammi, R.; Pomelli, C.; Ochterski, J. W.; Martin, R. L.; Morokuma, K.; Zakrzewski, V. G.; Voth, G. A.; Salvador, P.; Dannenberg, J. J.; Dapprich, S.; Daniels, A. D.; Farkas, O.; Foresman, J. B.; Ortiz, J. V.; Cioslowski, J.; Fox, D. J.; Gaussian 09, Revision D.01, Gaussian, Inc.: Wallingford CT, **2013**.

[7] Perdew, J. P.; Burke, K.; Ernzerhof. M. Generalized Gradient Approximation Made Simple. *Phys. Rev. Lett.* **1996**, *77*, 3865.

[8] Grimme, S.; Antony, J.; Ehrlich, S.; Krieg. H. A consistent and accurate ab initio parametrization of density functional dispersion correction (DFT-D) for the 94 elements H-Pu. *J. Chem. Phys*. **2010**, *132*, 154104.

[9] a) Hehre, W. J.; Ditchfield, R.; Pople. J. A.; Self—Consistent Molecular Orbital Methods. XII. Further Extensions of Gaussian—Type Basis Sets for Use in Molecular Orbital Studies of Organic Molecules. *J. Phys. Chem.* **1972**, *56*, 2257; b) Hariharan, P. C.; Pople. J. A. The influence of polarization functions on molecular orbital hydrogenation energies. *Theor. Chim. Acta.* **1973**, *28*, 213-222; c) Francl, M. M.; Pietro,

W. J.; Hehre, W. J.; Binkley, J. S.; Gordon, M. S.; Defrees, D. J.; Pople. J. A. Self-consistent molecular orbital methods. XXIII. A polarization-type basis set for second-row elements. *J. Chem. Phys.* **1982**, *77*, 3654.

[10] Andrae, D.; Haeussermann, U.; Dolg, M.; Stoll, H.; Preuss. H. Energy-adjustedab initio pseudopotentials for the second and third row transition elements. *Theor. Chim. Acta* **1990**, *77*, 123-141.

[11] Marenich, A. V.; Cramer, C. J.; Truhlar, D. G. Universal Solvation Model Based on Solute Electron Density and on a Continuum Model of the Solvent Defined by the Bulk Dielectric Constant and Atomic Surface Tensions. *J. Phys. Chem. B* **2009**, *113*, 6378-6396.

[12] Funes-Ardoiz, I.; Paton, R. S. GoodVibes v2.0.2. DOI: 10.5281/zenodo.595246.

[13] Ribeiro, R. F.; Marenich, A. V.; Cramer, C. J.; Truhlar. D. G. Use of Solution-Phase Vibrational Frequencies in Continuum Models for the Free Energy of Solvation. *J. Phys. Chem. B* **2011**, *115*, 14556-14562.
